# Changing socioeconomic and geographic gradients in cardiovascular disease risk factors among Indians aged 15–49 years – evidence from nationally representative household surveys

**DOI:** 10.1016/j.lansea.2023.100188

**Published:** 2023-04-14

**Authors:** Sarah Wetzel, Pascal Geldsetzer, Sneha Sarah Mani, Aashish Gupta, Kavita Singh, Mohammed K. Ali, Dorairaj Prabhakaran, Nikhil Tandon, Nikkil Sudharsanan

**Affiliations:** a Heidelberg Institute of Global Health, Heidelberg University, Heidelberg, Germany; b Division of Primary Care and Population Health, Department of Medicine, Stanford University, Stanford, CA, USA; c Chan Zuckerberg Biohub, San Francisco, CA, USA; d Graduate Group in Demography, University of Pennsylvania, PA, USA; e Harvard Center for Population and Development Studies, Harvard T.H. Chan School of Public Health, Harvard University, MA, USA; f Centre for Chronic Conditions and Injuries, Public Health Foundation of India, Gurugram, Haryana, India; g Hubert Department of Global Health, Rollins School of Public Health, Emory University, Atlanta, GA, USA; h Department of Family and Preventive Medicine, School of Medicine, Emory University, Atlanta, GA, USA; i Centre for Chronic Disease Control, India; j Public Health Foundation of India, India; k Department of Endocrinology and Metabolism, All India Institute of Medical Sciences, India; l Behavioral Science for Disease Prevention and Health Care, Technical University of Munich, Munich, Germany

**Keywords:** Cardiovascular diseases, Diabetes, Hypertension, Overweight, Obesity, Tobacco consumption, Smoking, Socioeconomic gradients, Socioeconomic status, Risk factor, India

## Abstract

**Background:**

Cardiovascular diseases (CVDs) are the leading cause of death in most low- and middle-income countries (LMICs). CVDs and their metabolic risk factors have historically been concentrated among urban residents with higher socioeconomic status (SES) in LMICs such as India. However, as India develops, it is unclear whether these socioeconomic and geographic gradients will persist or change. Understanding these social dynamics in CVD risk is essential for mitigating the rising burden of CVDs and to reach those with the greatest needs.

**Methods:**

Using nationally representative data with biomarker measurements from the fourth (2015–16) and fifth (2019–21) Indian National Family and Health Surveys, we investigated trends in the prevalence of four CVD risk factors: smoking (self-reported), unhealthy weight (BMI ≥25 $\frac{kg}{m^{2}}$), diabetes (random plasma glucose concentration ≥200 mg/dL or self-reported diabetes), and hypertension (one of: average systolic blood pressure ≥140 mmHg, average diastolic blood pressure ≥90 mmHg, self-reported past diagnosis, or self-reported current antihypertensive medication use) among adults aged 15–49 years. We first described changes at the national level and then trends stratified by place of residence (urban versus rural), geographic region (northern, northeastern, central, eastern, western, southern), regional level of development (Empowered Action Group member state or not), and two measures of socioeconomic status: level of education (no education, primary incomplete, primary complete, secondary incomplete, secondary complete, higher) and wealth (quintiles).

**Findings:**

Unhealthy weight increased among all social and geographic groups but both the absolute and the relative changes were substantially higher among people with low SES (as measured by education or wealth) and in rural areas. For diabetes and hypertension, the prevalence increased for those from disadvantaged groups while staying constant or even decreasing among the wealthier and more educated. In contrast, smoking consumption declined for all social and geographic groups.

**Interpretation:**

In 2015–16, CVD risk factors were higher among more advantaged subpopulations in India. However, between 2015–16 and 2019–21, the prevalence of these risk factors grew more rapidly for less wealthy and less educated subpopulations and those living in rural areas. These trends have resulted in CVD risk becoming far more widespread throughout the population; CVD can no longer be characterized as a wealthy urban phenomenon.

**Funding:**

This work was supported by the 10.13039/100005156Alexander von Humboldt Foundation (grant received by NS); the 10.13039/100014499Stanford Diabetes Research Center [grant received by PG] and the Chan Zuckerberg Biohub [grant received by PG].

**Research in context**

**Evidence before this study**

We searched PubMed for work published between Jan 1, 1990, and Sep 23, 2022, with variations of the search terms “reversal hypothesis”, “social gradient”, “socioeconomic gradient”, “social difference”, “socioeconomic difference”, “socioeconomic status”, “change”, “trend”, “cardiovascular disease”, “cardiovascular risk factor”, “diabetes”, “hypertension”, “overweight”, “obesity”, “smoking”, “tobacco”, “low-income”, “lower-middle-income”, and “India” in the title or abstract. Existing studies on changes in the socioeconomic patterning of cardiovascular disease (CVD) risk factors mostly compared the size of social gradients in obesity cross-sectionally between countries at different levels of national income or development. These studies generally found higher obesity among higher socioeconomic status (SES) populations but opposite gradients for countries at higher levels of development. However, because these studies use cross-sectional comparisons, whether these patterns reflect the influence of development or other contextual factors associated with countries at different levels of development is unclear. Both for obesity and for other CVD risk factors, we found few nationally-representative studies that traced how gradients within countries changed over time as they developed. Importantly, most of these studies did not focus on India and those that did only considered single risk factors such as obesity using older data. Existing nationally representative research on India is thus predominantly cross-sectional and has only focused on the size of social gradients at single points in time. This work generally finds higher levels of unhealthy weight, diabetes, and hypertension among wealthy and educated subpopulations but lower levels of smoking. Subnational and smaller non-representative studies from India that traced changes in CVD risk factors over time provide some evidence of reversing or flattening gradients but it is unclear whether these patterns hold at regional and national levels.

**Added value of this study**

Using data from the 2015–16 and 2019–2021 National Family and Health Surveys, we provide some of the first nationally representative evidence for trends in the prevalence of major CVD risk factors in India and how these trends have changed across social and geographic groups. Both absolute and relative increases in prevalence were far more pronounced among relatively poor and less educated populations. As a result of these trends, most CVD risk factors became more equal across population groups, or in some cases, became higher among the less compared to more educated.

**Implications of all the available evidence**

Indians with lower SES – as measured by wealth and education – and people living in rural areas experienced considerable adverse trends in unhealthy weight, diabetes, and hypertension between 2015–16 and 2019–21. Our results reveal that CVDs can no longer be considered a problem of the affluent parts of society and suggest that CVD prevention efforts that reach less advantaged subpopulations are urgently needed.

## Introduction

Cardiovascular diseases (CVDs) are the leading worldwide causes of death, accounting for 32% of all deaths globally.^1^^,^^2^ The risk of CVD can be substantially reduced by addressing key behavioral and metabolic risk factors, particularly smoking, hypertension, diabetes, and unhealthy weight.^3^^,^^4^ For this reason, monitoring the prevalence of these key risk factors is essential to identify populations at high risk and develop targeted public health interventions.

Previous studies from India and from other low- and middle-income countries (LMICs) generally find a higher prevalence of hypertension, diabetes, and unhealthy weight among people with higher compared to those with lower socioeconomic status (SES).^5^, ^6^, ^7^, ^8^ For example, based on data from the 2015–16 Indian National Family and Health Survey (NFHS), the prevalence of obesity was 18.6% among individuals in the highest household wealth quintile compared to just 1.7% among those in the lowest wealth quintile. Similarly, the prevalence of hypertension was 16.1% in urban compared to 13.4% in rural areas.^7^ Such evidence of CVD risk as an urban and upper-class phenomenon has resulted in calls to prioritize health conditions like maternal and child health or infectious diseases instead of allocating public resources to CVD care.^9^

In contrast to India and other LMICs, high-income countries and several upper-middle-income countries are typically characterized by a higher prevalence of CVD risk factors among disadvantaged subpopulations.^10^, ^11^, ^12^ This stark contrast has led to the hypothesis that the social pattern of prevalence might reverse as countries develop: while CVD risk is initially more concentrated among higher SES populations, it will become more pronounced among poorer subpopulations in the course of social and economic development.^10^^,^^13^^,^^14^ If true, CVD risk factors in India may also be changing and becoming more widespread throughout the population. Indeed, recent studies have found that some CVD risk factors like hypertension are equally high across all wealth groups in similar LMICs like Indonesia,^14^, ^15^, ^16^, ^17^ and subnational analyses within India also find first signs of reversal in more developed parts of the country.^5^^,^^6^^,^^18^

It is currently unknown how SES differences in CVD risk factors (known as SES gradients) have changed over time in India. This gap is likely because repeated nationally representative data has not been available so far for many major CVD risk factors.^19^ We address this key research gap by taking advantage of recently released nationally representative survey data with biomarker measurements. We begin by providing comprehensive evidence on changes in the prevalence of major behavioral and biological CVD risk factors. After showing trends at the national level, we measure changes separately across socioeconomic and geographic groups. Finally, we examine the implications of these trends for SES and geographic gradients and assess whether India is undergoing a shift of CVD risk towards more disadvantaged segments of the population.

## Methods

### Data

We used data from two consecutive nationally representative cross-sectional household surveys from India, the NFHS-4 and the NFHS-5. Both surveys used a complex two-stage stratified sampling design and a nearly identical sampling strategy. Commissioned by the Ministry of Health and Family Welfare, Government of India and with technical assistance from the Demographic and Health Surveys Program, the International Institute for Population Sciences (IIPS) conducted the two surveys from 2015 to 2016 (NFHS-4) and from 2019 to 2021 (NFHS-5). First, the IIPS selected primary sampling units (PSUs) within strata applying systematic random sampling with probability proportional to size. Villages and Census Enumeration Blocks served as PSUs in rural and urban areas, respectively. Afterwards, the institute chose 22 households from each PSU or – in PSUs with more than 300 households – from 2 out of three segments of a PSU (again through systematic random sampling).^19^^,^^20^

Both the NFHS-4 and the NFHS-5 included questions on self-reported tobacco use (smoking and smokeless variants), current diabetes status (based on the question “Do you currently have diabetes?”), clinical history of hypertension (based on the questions “Were you told on two or more different occasions by a doctor or other health professional that you had hypertension or high blood pressure?” (NFHS-4) and “Has a doctor, nurse or auxiliary nurse midwife told you two or more times that you have hypertension or high blood pressure?” (NFHS-5)), and current intake of prescribed antihypertensive medication (based on the question “Are you now taking prescribed medication to lower your blood pressure?”). Furthermore, trained enumerators also collected anthropometric and biomarker measurements, including height, weight, random blood glucose, and three blood pressure readings with 5-min intervals between measurements. In the NFHS-5, adults aged 15 years and older were eligible for glucose tests, blood pressure measurements, and asked about hypertension diagnoses or treatments while all other mentioned CVD-related items (diabetes status, tobacco use, height, and weight) in the NFHS-5 and all CVD items in the NFHS-4 only covered men aged 15–54 in a representative subsample of 15% of households (state module) and women aged 15–49 years.

The methods to measure biomarkers were consistent across survey rounds and the devices nearly equivalent. Weight, height, glucose, and blood pressure measurements were measured using the Seca 874 digital scale, the Seca 213 stadiometer, the FreeStyle Optium H glucometer (NFHS-4) or the Accu-Chek Performa glucometer (NFHS-5), and an Omron Blood Pressure Monitor, respectively.

### Main outcomes and variable definitions

We analyzed trends in four CVD risk factors: smoking, unhealthy weight, hypertension, and diabetes. We measured smoking as a binary indicator for self-reported use of cigarettes, pipe, cigars, bidis, or Hookah.

We measured unhealthy weight using a binary indicator for overweight or obesity. For our main analysis, we classified participants with a BMI of at least 25 $\frac{kg}{m^{2}}$ as having overweight. In the appendix, we also present results for trends in unhealthy weight using an indicator for obesity only (defined as BMI≥30 $\frac{kg}{m^{2}}$) (Figs. S11-S13 and S37-S39 in the appendix).

We measured diabetes with a binary indicator for self-reported diabetes status or a random plasma glucose concentration ≥200 $\frac{mg}{dL}$. Our diabetes definition does not consider antidiabetic medication, since the NFHS-4 survey instruments did not include this question.

We measured hypertension with a binary indicator if at least one out of four conditions was satisfied: the respondent reported a past diagnosis by a clinician, reported being on an antihypertensive medication, had an average systolic blood pressure greater than or equal to 140 mmHg, or had an average diastolic blood pressure greater than or equal to 90 mmHg. As the first blood pressure measurement generally tends to be higher and might therefore overestimate the prevalence of hypertension,^21^^,^^22^ we calculated average systolic and diastolic blood pressure based on the second and third reading. If one of these two readings was missing, we replaced it by the first. We excluded participants with only a single reading of systolic or diastolic blood pressure. We also excluded individuals with implausible biomarker values: systolic blood pressure below 70 mmHg or above 240 mmHg, diastolic blood pressure below 40 mmHg or above 130 mmHg, or random blood glucose below 40 mg/dL. If any of the variables needed to define an indicator was not available, we set the respective indicator to missing.

### Socioeconomic and geographic subgroups

As part of our analysis, we stratified results by sex (measured by a binary variable for male versus female), age (measured in 5-year age groups), level of education (classified as: no education, primary incomplete, primary complete, secondary incomplete, secondary complete, or higher), household wealth (measured in quintiles of a continuous index based on household asset ownership, housing materials, and water and sanitation access^23^^,^^24^), place of residence (measured by a binary variable for urban versus rural areas), geographic region (grouping northern, northeastern, central, eastern, western, and southern administrative divisions as defined by the NFHS^20^), and regional level of development measured by a binary variable for households living in one of the eight Empowered Action Group (EAG) states (Bihar, Chhattisgarh, Jharkhand, Madhya Pradesh, Orissa, Rajasthan, Uttarakhand, and Uttar Pradesh). In 2001, the Government of India constituted this EAG with the aim of fostering development in India's less developed states.^25^ Except for the location-based indicators, all these variables were based on household or respondent self-reports.

### Statistical methods

We restricted our analysis to individuals aged 15–49 who, regardless of sex and survey round, were eligible for biomarker measurements and asked about their health behavior/status. Furthermore, we excluded pregnant women and participants who replied with “Don't know” when asked about their level of education. To arrive at the final sample for the analysis of each outcome, we dropped observations with missing values in the respective dependent variable (i.e., the sample sizes differed depending on the outcome).

After describing the unweighted samples by survey year, we first report year-specific prevalence levels, absolute changes, and changes in percentage terms relative to the NFHS-4 at the national level for each of the CVD risk factors. We estimated these changes over time based on log binomial regression models with the respective CVD risk factor as the dependent variable and a binary indicator for the NFHS-5 as our main independent variable which captures the overall change in each CVD risk factor over time. We applied Stata's “margins” command to the coefficient of this survey year indicator to obtain changes on both the absolute and relative scales.

We then measured changes in the risk factors across socioeconomic and geographic groups to identify disproportionately affected subpopulations and determine how gradients have changed over time. To do this, we estimated similar regression models as for the national level additionally including one of the grouping variables (to capture the baseline social gradients in the NFHS-4) and an interaction term between the survey year dummy and each social and geographic grouping variable (to measure how the gradients have changed over time). We only included one grouping variable in the regression model at a time. Regression coefficients along with the corresponding confidence intervals are available in the appendix (Table S4-S9).

All steps of our analysis factor in the complex survey design, including the survey weights. However, we performed two adjustments to the original NFHS weights: given the subsampling of men as described above, all outcomes except for the hypertension indicator in the NFHS-5 were only collected for a subset of men aged 15–49 but for all women in this age group. We rescaled the survey weights of men in the datasets for the analysis of diabetes, smoking, and unhealthy weight and the survey weights of male observations from the NFHS-4 in the hypertension dataset to ensure an equal representation of men and women. Secondly, we applied direct age standardization to the survey weights (using the age distribution of the WHO standard population)^26^ such that all our estimates of CVD trends are net of demographic changes as Asian countries are faced with substantial aging and CVD risk factors are positively correlated with age.^27^, ^28^, ^29^ In the appendix (Table S2 and Figs.S2–S43), we also show unadjusted changes based on the Indian age distribution in the respective survey year according to the United Nations World Population Prospects from 2019 (UNWPP).^30^ We used R version 4.1.0 and Stata 17.0 for our analysis.

### Role of the funding sources

The funders of the study had no role in study design, data collection, data analysis, data interpretation, writing of the report, or the decision to submit for publication.

## Results

### Sample characteristics

Taken together, the NFHS-4 and the NFHS-5 collected data from 2,350,441 individuals aged 15–49 (NFHS-4: 866,304, NFHS-5: 1,484,137). After excluding pregnant women, people with unknown level of education, and participants with missing outcome variable, the final samples for the analysis of smoking, unhealthy weight, hypertension, and diabetes were 1,558,135 (NFHS-4: 769,373, NFHS-5: 788,762), 1,539,701 (NFHS-4: 768,881, NFHS-5: 770,820), 1,993,650 (NFHS-4: 757,047, NFHS-5: 1,236,603), and 1,481,070 (NFHS-4: 739,989, NFHS-5: 741,081) individuals, respectively. A flowchart showing the sampling procedure is available in the appendix (Fig. S1).

The unweighted social and demographic characteristics were similar across the different outcome samples and survey rounds (Table 1). Around 88% of all the samples consisted of female respondents, except for the NFHS-5 hypertension sample (53% female), and between 50 and 52% of the samples were above the age of 30. With respect to education, between 17 and 25% of the samples had no formal education and between 12 and 16% completed more than secondary education. The share of respondents from urban households ranged from 25 to 30%. We present the weighted descriptive statistics in Table S1 in the appendix.

**Table 1:** Unweighted descriptive characteristics of the study samples, National Family Health Survey 4 (2015–16) and 5 (2019–21), India.

	Tobacco sample		Overweight sample		Diabetes sample		Hypertension sample	
	NFHS-4 *N = 769,373*	NFHS-5 *N = 788,762*	NFHS-4 *N = 768,881*	NFHS-5 *N = 770,820*	NFHS-4 *N = 739,989*	NFHS-5 *N = 741,081*	NFHS-4 *N = 757,047*	NFHS-5 *N = 1,236,603*
**Sex**								
Male	103,297 (13%)	93,231 (12%)	102,788 (13%)	89,676 (12%)	98,656 (13%)	85,715 (12%)	101,049 (13%)	577,017 (47%)
Female	666,076 (87%)	695,531 (88%)	666,093 (87%)	681,144 (88%)	641,333 (87%)	655,366 (88%)	655,998 (87%)	659,586 (53%)
**Age group**								
15–19 years	140,138 (18%)	136,151 (17%)	139,926 (18%)	131,249 (17%)	134,730 (18%)	127,137 (17%)	137,264 (18%)	211,968 (17%)
20–24 years	125,648 (16%)	121,909 (15%)	126,383 (16%)	119,284 (15%)	121,141 (16%)	114,828 (15%)	124,393 (16%)	190,085 (15%)
25–29 years	121,164 (16%)	123,519 (16%)	121,450 (16%)	121,223 (16%)	116,690 (16%)	116,882 (16%)	119,573 (16%)	192,308 (16%)
30–34 years	107,797 (14%)	110,709 (14%)	107,524 (14%)	108,540 (14%)	103,864 (14%)	104,630 (14%)	105,996 (14%)	174,033 (14%)
35–39 years	102,906 (13%)	109,703 (14%)	102,568 (13%)	107,445 (14%)	98,949 (13%)	103,170 (14%)	101,095 (13%)	172,111 (14%)
40–44 years	88,146 (11%)	91,965 (12%)	87,806 (11%)	90,142 (12%)	84,636 (11%)	85,990 (12%)	86,627 (11%)	145,683 (12%)
45–49 years	83,574 (11%)	94,806 (12%)	83,224 (11%)	92,937 (12%)	79,979 (11%)	88,444 (12%)	82,099 (11%)	150,415 (12%)
**Level of education**								
No education	192,447 (25%)	163,166 (21%)	192,895 (25%)	160,822 (21%)	186,098 (25%)	153,784 (21%)	190,622 (25%)	205,635 (17%)
Incomplete primary	49,529 (6.4%)	45,449 (5.8%)	49,555 (6.4%)	44,845 (5.8%)	47,379 (6.4%)	42,804 (5.8%)	48,732 (6.4%)	69,725 (5.6%)
Complete primary	52,918 (6.9%)	52,543 (6.7%)	53,205 (6.9%)	51,560 (6.7%)	51,136 (6.9%)	49,444 (6.7%)	52,595 (6.9%)	79,572 (6.4%)
Incomplete secondary	312,489 (41%)	331,035 (42%)	312,251 (41%)	323,582 (42%)	300,274 (41%)	311,628 (42%)	306,889 (41%)	545,798 (44%)
Complete secondary	69,671 (9.1%)	83,246 (11%)	69,620 (9.1%)	81,009 (11%)	67,073 (9.1%)	78,250 (11%)	68,385 (9.0%)	142,861 (12%)
Higher than secondary	92,319 (12%)	113,323 (14%)	91,355 (12%)	109,002 (14%)	88,029 (12%)	105,171 (14%)	89,824 (12%)	193,012 (16%)
**Wealth quintile**								
Poorest	142,309 (18%)	161,000 (20%)	143,283 (19%)	158,867 (21%)	137,470 (19%)	151,735 (20%)	141,458 (19%)	254,390 (21%)
Poorer	163,112 (21%)	174,522 (22%)	163,967 (21%)	172,049 (22%)	157,066 (21%)	165,078 (22%)	161,436 (21%)	274,763 (22%)
Middle	162,850 (21%)	165,645 (21%)	163,093 (21%)	162,821 (21%)	156,851 (21%)	156,856 (21%)	160,251 (21%)	262,114 (21%)
Richer	154,148 (20%)	152,987 (19%)	153,554 (20%)	149,184 (19%)	148,182 (20%)	144,072 (19%)	151,046 (20%)	239,889 (19%)
Richest	146,954 (19%)	134,608 (17%)	144,984 (19%)	127,899 (17%)	140,420 (19%)	123,340 (17%)	142,856 (19%)	205,447 (17%)
**Place of residence**								
Urban	229,285 (30%)	198,099 (25%)	226,286 (29%)	189,467 (25%)	218,102 (29%)	182,199 (25%)	222,783 (29%)	306,476 (25%)
Rural	540,088 (70%)	590,663 (75%)	542,595 (71%)	581,353 (75%)	521,887 (71%)	558,882 (75%)	534,264 (71%)	930,127 (75%)

### Trends at the national level

From 2015–16 to 2019–21, the largest absolute increase in prevalence occurred for unhealthy weight (overweight: 3.4 percentage points, 95% CI: 3.0, 3.8; obesity: 1.2 percentage points, 95% CI 1.0, 1.3) followed by diabetes (0.2 percentage points, 95% CI 0.1, 0.3) (Table 2). While the absolute changes appear small, these increasing trends corresponded to pronounced relative changes when compared to the baseline NFHS-4 values: overweight increased by 17% (95% CI 14.7, 19.3) from a baseline prevalence of 20% (95% CI 19.7; 20.3), obesity by 28.4% (95% CI 23.7, 33.2) from a baseline prevalence of 4.1% (95% CI 4.0, 4.2), and diabetes by 7.4% (95% CI 2.1, 13.0) from a baseline prevalence of 2.8% (95% CI 2.7, 2.9).

**Table 2:** Levels, absolute and relative changes in 4 CVD risk factors at the national level in India based on the National Family Health Survey 4 (2015–16) and 5 (2019–21).

Outcome	NFHS-4	NFHS-5	Absolute change (percentage points)	Relative change (%)
Smoking	12.6 [12.3; 12.9]	9.4 [9.1; 9.7]	−3.2 [−3.7; −2.8]	−25.7 [−28.7; −22.5]
Overweight	20.0 [19.7; 20.3]	23.4 [23.1; 23.6]	3.4 [3; 3.8]	17.0 [14.7; 19.3]
Diabetes	2.8 [2.7; 2.9]	3.0 [2.9; 3.1]	0.2 [0.1; 0.3]	7.4 [2.1; 13]
Hypertension	17.8 [17.5; 18.1]	16.7 [16.5; 16.8]	−1.1 [−1.5; −0.8]	−6.4 [−8.2; −4.6]

Over the period, hypertension slightly decreased by 1.1 percentage points nationally (95% CI −1.5, −0.8) while smoking dropped by 3.2 percentage points (95% CI −3.7, −2.8). The latter downward trend was mostly attributable to a lower consumption of smoked tobacco variants other than cigarettes (Table S3). In relative terms, these changes corresponded to a decline of 6.4% (95% CI −8.2, −4.6) in hypertension and a decline of 25.7% (95% CI −28.7, −22.5] in the share of the population that smokes.

### Changing socioeconomic gradients

**Fig. 1:**
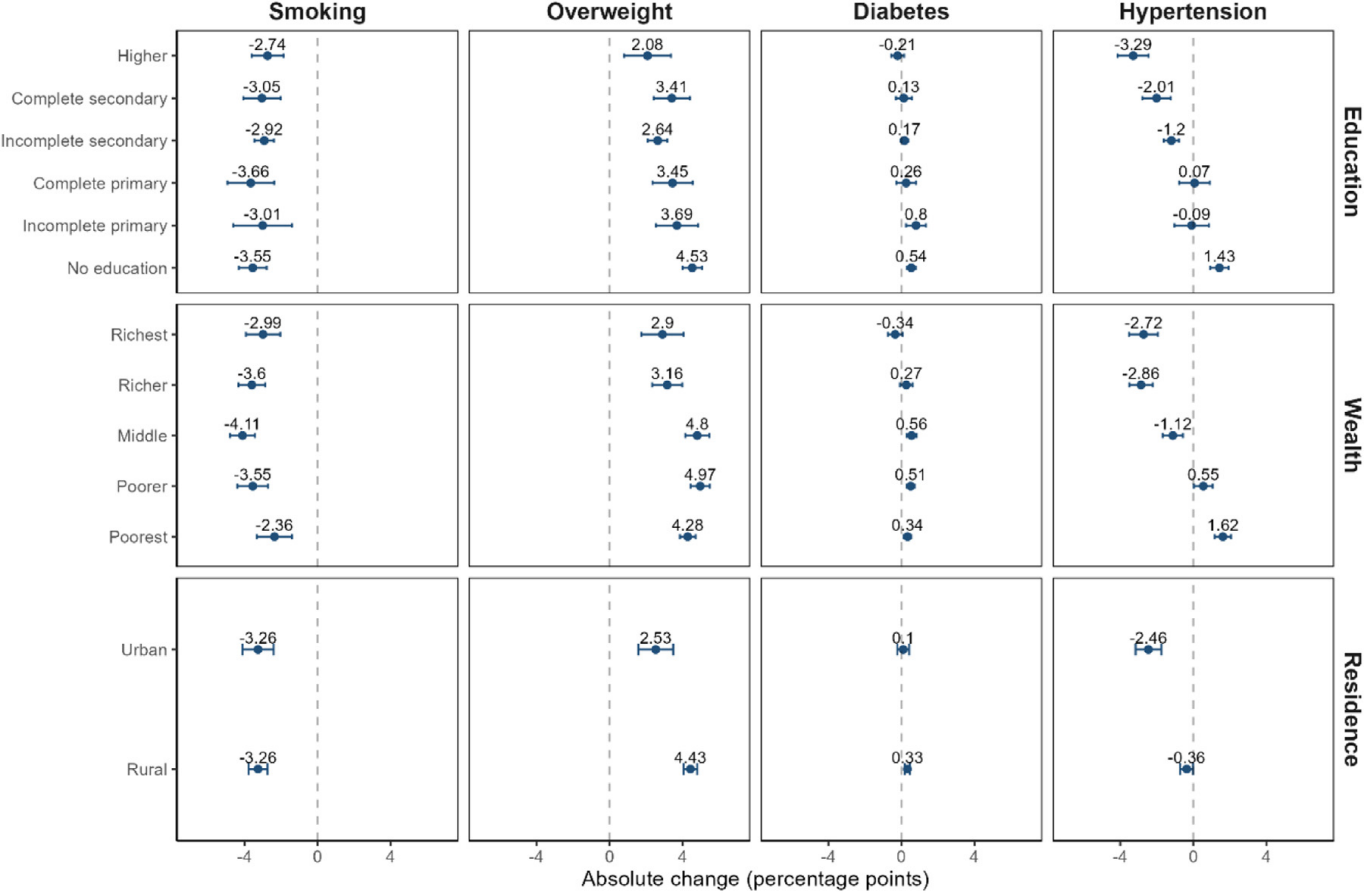
**Age-standardized absolute change in each CVD risk factor among adults aged 15–49 by wealth quintile, level of education, and place of residence, National Family Health Survey 4 (2015–16) and 5 (2019–21), India**.

**Fig. 2:**
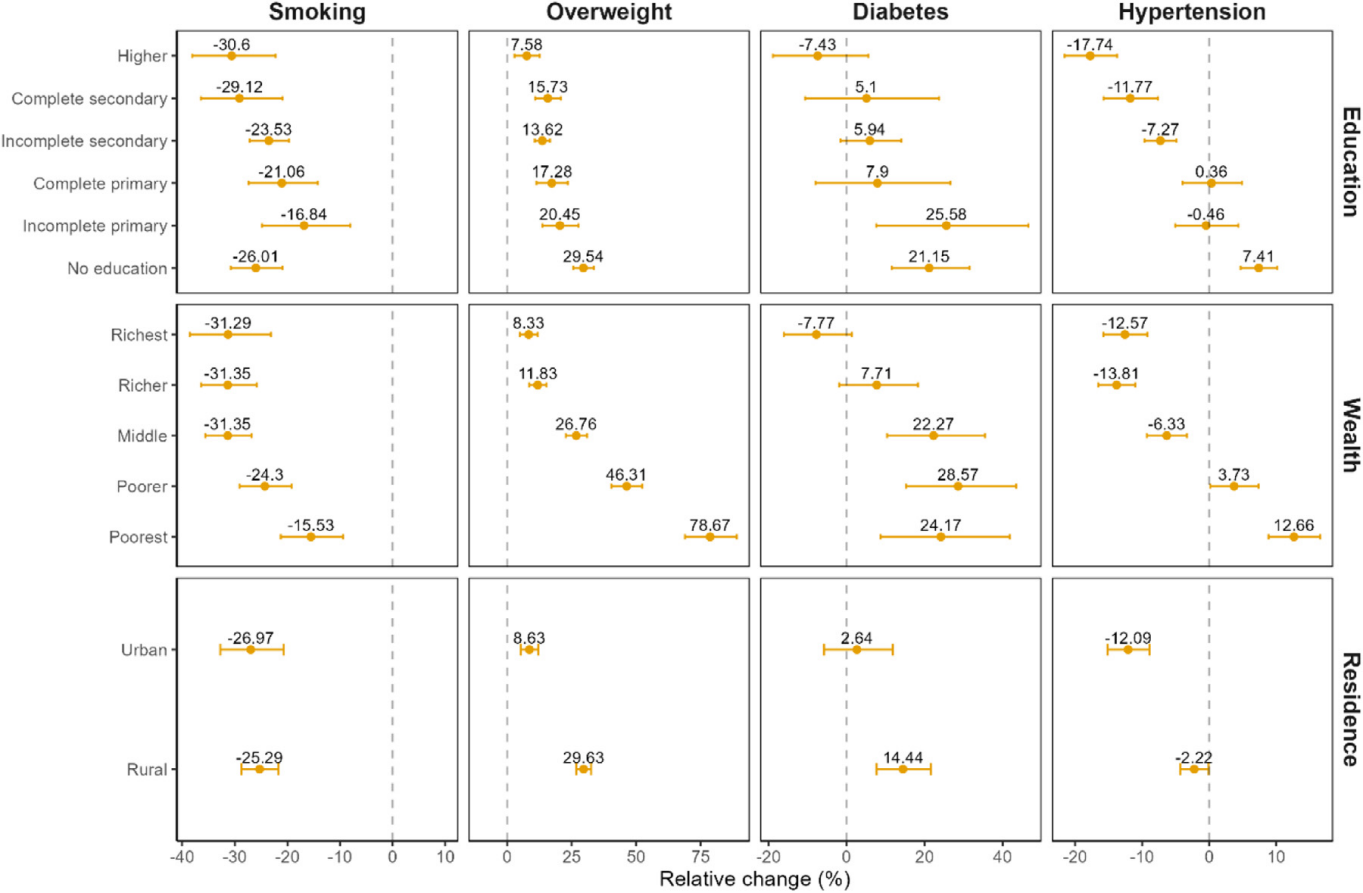
**Age-standardized relative change in each CVD risk factor among adults aged 15–49 by wealth quintile, level of education, and place of residence, National Family Health Survey 4 (2015–16) and 5 (2019–21), India**.

In 2015–16, the prevalence of both overweight and obesity was higher among wealthier and educated NFHS-4 participants (Figs. S51 and S52 in the appendix). However, the magnitude of the change in overweight between 2015–16 and 2019–21 was substantially higher among people with low SES compared to people with high SES (Figs. 1 and 2): Overweight increased by 4.5 percentage points (95% CI 4.0, 5.1) among Indians without education compared to just 2.1 percentage points (95% CI 0.8, 3.4) among the most educated. This corresponded to a 29.5% (95% CI 25.7, 33.6) increase among the uneducated compared to a just 7.6% (95% CI 2.8, 12.6) increase among those with the highest level of education. Similarly, the poorest 20% were faced with a stark rise of 4.3 percentage points (95% CI 3.9, 4.7) in overweight while the richest 20% of the population experienced a more modest change of 2.9 percentage points (95% CI 1.8, 4.1). In relative terms, these upward trends were equivalent to increases of 78.7% (95% CI 68.9, 89.0) in the first and 8.3% (95% CI 4.9, 11.9) in the fifth quintile.

For diabetes, the size of the SES gradient in 2015–16 varied across the two measures of SES (Fig. S61 in the appendix). Across wealth, the prevalence of diabetes was 1.4% (95% CI 1.3, 1.5) among those in the poorest wealth quintile compared to 4.4% (95% CI 4.1, 4.7) in the highest wealth quintile. In contrast, the prevalence of diabetes in 2015–16 was similar across all education groups. Despite these baseline differences, we find that the increase in the prevalence of diabetes between 2015–16 and 2019–21 was larger for disadvantaged groups across both measures of SES (Figs. 1 and 2). The prevalence of diabetes increased by 0.3 percentage points (95% CI 0.1, 0.5; relative increase of 24.2%) for those in the bottom wealth quintile compared to a 0.3 percentage points decrease (95% CI −0.7, 0.1; relative decrease of 7.8%) for those in the top wealth quintile. Among those without any education, diabetes rose by 0.5 percentage points (95% CI 0.3, 0.8; relative increase of 21.2%), whereas prevalence among people with more than secondary education remained virtually unchanged (−0.2 percentage points, 95% CI −0.6, 0.1). These changes led to a substantial flattening in the wealth gradients and a complete reversal in education gradients for diabetes between 2015–16 and 2019–21.

Like diabetes, we find that individuals with lower levels of education and wealth experienced a much larger change in the prevalence of hypertension between 2015–16 and 2019–21 (Figs. 1 and 2). Hypertension increased by 1.4 percentage points (95% CI 0.9, 1.9; relative change of 7.4%) for those without education but decreased by 3.3 percentage points (95% CI −4.1, −2.4; relative decrease of 12.6%) for those with the highest level of education. Likewise, hypertension rose by 1.6 percentage points (95% CI 1.2, 2.1; relative increase of 12.7%) in the poorest wealth quintile while it declined by 2.7 percentage points (95% CI −3.5, −1.9; 12.6% relative decline) in the top wealth quintile. Due to these changes over time, SES gradients in hypertension measured by education have fully reversed and the prevalence of hypertension is now higher among those with lower compared to higher SES. As of 2019–21, the prevalence of hypertension is 15.2%, (95% CI 14.9, 15.6) among the most educated compared to 20.7%, (95% CI 20.4, 21.0) among those without education.

Both the baseline gradients and trends over time in smoking differed substantially from the other CVD risk factors. In 2015–16, the prevalence was greater among those in the lowest (15.2%, 95% CI 14.6, 15.9) compared to the highest (9.5%, 95% CI 8.8, 10.3) wealth quintiles, and, among those with no education (13.6%, 95% CI 13.1, 14.2) compared to those with more than secondary education (9.0%, 95% CI 8.3, 9.7) (Fig. S44 in the appendix). Between 2015–16 and 2019–21, the absolute decline was greater for those with lower SES; however, because of the higher initial prevalence, this absolute decline corresponded to a smaller relative change compared to those with higher SES (Figs. 1 and 2). For example, tobacco use declined by 6.7 percentage points (95% CI −7.6, −5.8) for those with no education compared to 4.0 percentage points (95% CI −5.0, −3.0) among those with the highest level of education. However, this corresponded to a 26.0% decrease (95% CI −30.8, −20.9) for the uneducated compared to a 30.6% decrease (95% CI −38.1, −22.2) for the most educated group.

These shifts in the burden of unhealthy weight, hypertension, and diabetes to people with low SES affected both sexes, although the magnitude of the gradient changes tended to be higher among women (Figs S84–S87 in the appendix). Furthermore, our results are similar when we consider the prevalence of elevated blood glucose and blood pressure alone (Figs. S66, S67, S73, and S74 in the appendix).

### Changing urban-rural gradients

Between the NFHS 4 and 5, rural areas were adversely affected by trends in CVD risk factors compared with urban areas. In 2015–16, the prevalences of overweight, diabetes, and hypertension were 14.3, 1.5, and 3.9 percentage points higher among urban residents, respectively (Figs. S51, S61, and S71 in the appendix). However, rural areas experienced a faster increase in the prevalence of these risk factors between 2015–16 and 2019–21 (Figs. 1 and 2): overweight increased by 4.4 percentage points (95% CI 4.1, 4.8; relative change 29.6%) in rural compared to 2.5 percentage points (95% CI 1.6, 3.5; relative change 8.6%) in urban areas; diabetes increased by 0.3 percentage points (95% CI 0.2, 0.5; relative change 14.4%) in rural compared to 0.1 percentage points (95% CI −0.2, 0.4; relative change 2.6%) in urban areas; hypertension decreased by 0.4 percentage points (95% CI −0.7, 0; relative change −2.2%) in rural compared to a −2.5 percentage points decline (95% CI −3.2, --1.8; relative change −12.1%) in urban areas. Rural and urban areas experienced an equivalent absolute decline in smoking but the higher prevalence among rural people in 2015–16 (urban: 12.1%; rural: 12.9%) resulted in more pronounced relative changes in urbanized areas (urban: relative change −27.0%; rural: relative change −25.3%) (Fig. S44 in the appendix, Figs. 1 and 2).

### Changing regional gradients

**Fig. 3:**
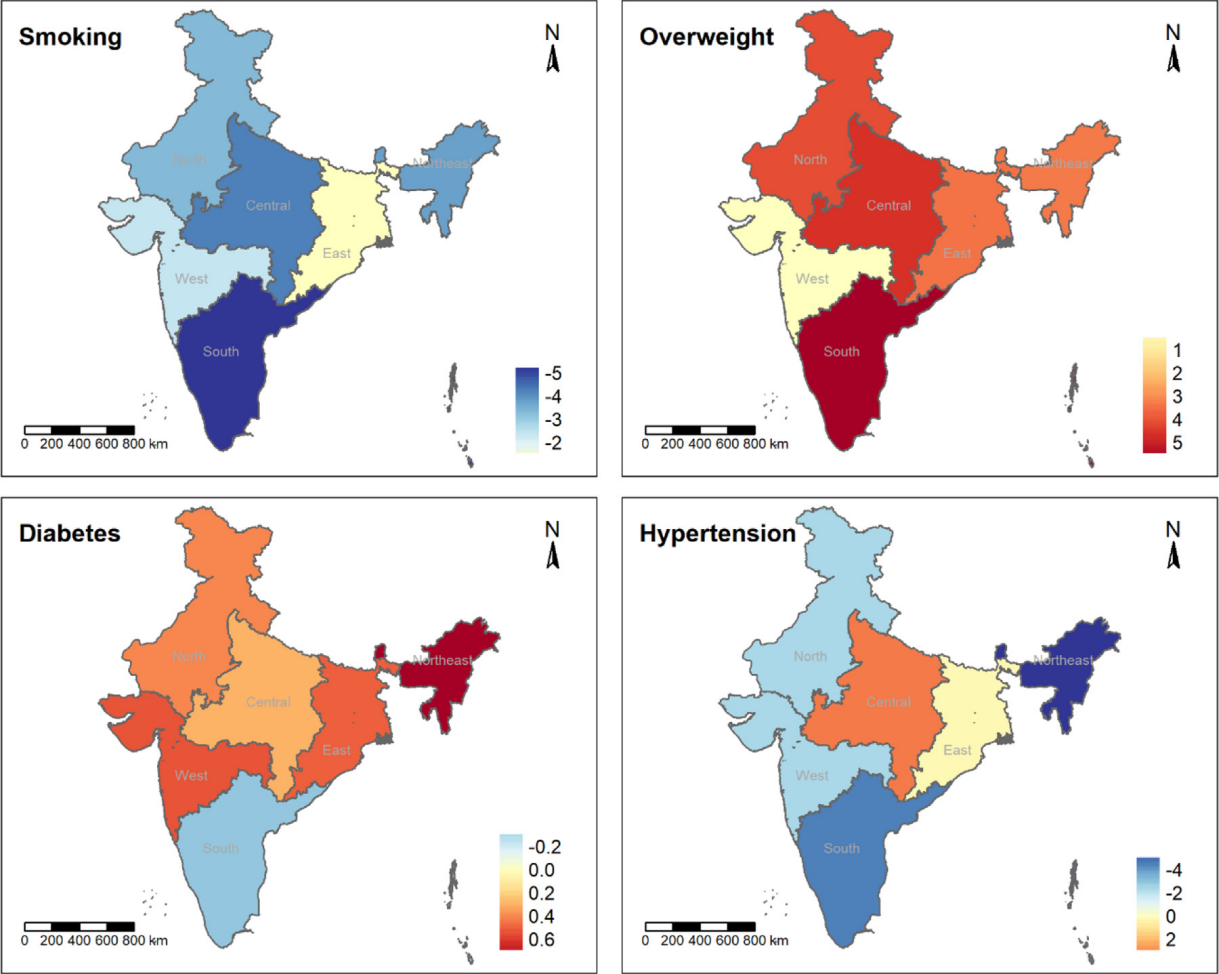
**Age-standardized absolute changes (percentage points) in the prevalence of CVD risk factors across Indian regions, National Family Health Survey 4 (2015–16) and 5 (2019–21), India**.

The prevalences of unhealthy weight, diabetes, and hypertension were highest in south India in both 2015–16 and 2019–21 (Fig. S78 in the appendix). While the disproportionate overweight burden in the south compared to the other regions became even more pronounced (absolute change in the south: 5.7 percentage points, 95% CI 4.8, 6.7), the geographic disparities in diabetes and hypertension across regions decreased (Fig. 3 and Fig. S79 in the appendix). Interestingly, the hypertension gradient between the central region and the south decreased from nearly ten to just 1.5 percentage points in 2019–21 (central: 17.1%, 95% CI 16.8, 17.0; south: 18.5%, 95% CI 18.2, 18.4).

In contrast to the other CVD risk factors, smoking was and, as of 2019–21, remains highest in the northeastern region (2015–16: 19.7%, 95% CI 18.6, 20.9; 2019–21: 16.0%, 95% CI 14.9, 16.9; Fig. S78 and S79 in the appendix). All regions experienced decreasing smoking trends (Fig. 3 and Fig. S79 in the appendix). However, the strongest absolute and relative decline occurred in the south (−5.3 percentage points, 95% CI −6.2, −4.3; relative change −41.1%) where the share of smoking people was initially already relatively low (only the Western part of the country was less affected).

Adults living in EAG member states had substantially lower CVD risk factor levels in 2015–16 (Fig. S81 in the appendix). For example, overweight was more than 10 percentage points lower in the EAG region (EAG: 13.8, 95% CI 13.5, 14.0; non-EAG: 24.5%, 95% CI 24.0, 25.0). Over time, hypertension rose in EAG states (2.6 percentage points, 95% CI 2.2, 2.9) but declined in non-EAG states (−3.5 percentage points, 95% CI −4.0, −2.9, Figs. S82 and S83 in the appendix). Similarly, EAG members were more affected by increasing overweight, particularly in relative terms (EAG: 27.1%, 95% CI 23.9, 30.3; non-EAG: 14.6%, 95% CI 11.7, 17.5). The magnitude of the decrease in smoking and the increase in diabetes were similar in both groups of states.

## Discussion

We found that people with lower SES measured by education or wealth and those living in rural areas experienced stronger adverse trends in unhealthy weight, hypertension, and diabetes between 2015–16 and 2019–21. While these risk factors were initially higher among higher SES and urban populations, these trends over time resulted in a substantial flatting or even reversal of SES gradients to the disadvantage of relatively poor and less educated subpopulations. For instance, the prevalence of overweight surged from around 5% to 10% among adults in the poorest wealth quintile (a relative increase of nearly 80%) compared to a change of less than 3 percentage points (relative change 8%) among adults in the richest wealth quintile. Importantly, these findings are not just the result of changes in risk factor diagnosis but rather due to secular changes in risk. The patterns we observe for unhealthy weight are based on measured height and weight and although our definitions of diabetes and hypertension consider diagnosis, we find similar trends when just focusing on measured blood glucose and blood pressure. Our results reveal that CVDs can no longer be considered a problem of the affluent parts of society and suggest that CVD prevention efforts that reach less advantaged subpopulations are needed. This need is especially pressing as lower SES groups form the majority of India's massive population yet have much higher difficulty accessing care and treatment.^31^, ^32^, ^33^

Our results are consistent with prior subnational studies from India, which identified early signs of reversing socioeconomic gradients in more developed settings of the country. Both urban subpopulations and more affluent states showed a higher prevalence of CVD risk factors among people with lower SES while opposite gradients prevailed elsewhere.^5^^,^^6^^,^^18^^,^^34^^,^^35^ Our study combined with the existing literature demonstrates that changes in the social patterning of CVD risk are taking place throughout the country and at the national level.

The changing socioeconomic and geographic gradients we observe are consistent with the “reversal hypothesis” that as countries develop, CVD risk goes from being disproportionate among the rich to disproportionate among the poor.^10^^,^^14^^,^^36^ To what extent gradients have already reversed, however, differed depending on the applied definition of SES. Measuring SES by the level of education, we find that gradients have already reversed to a large extent: unhealthy weight is the only CVD risk factor that remained higher among more educated individuals in 2019–21, while the other risk factors showed higher levels among those in the lowest education groups. In contrast, when using wealth as an indicator of SES, gradient reversal was still in the early stages: as of 2019–21, the prevalence of diabetes, hypertension, and unhealthy weight was still higher in the more affluent parts of the population, although these groups became more equal in terms of prevalence over time. These findings are in line with systematic reviews reporting a sensitivity of results to the SES definition, including the level of development at which reversal occurs.^11^^,^^37^

As reported in other studies,^38^ geographic disparities regarding the prevalence of CVD risk factors are substantial in India. While previously less affected rural areas are increasingly catching up with urbanized centers, the trends across regions and depending on EAG membership only partly indicate a geographic convergence and this tendency was mainly limited to diabetes and hypertension.

As a response to alarming predictions regarding the expected future CVD burden and a corresponding threat to sustainable development, the Indian government in 2012 adopted a national action plan to prevent and control non-communicable diseases (NCD). This plan defined 9 intermediate targets for 2020 and 10 long-term targets for 2025, including a reduction of hypertension (2020: 10%, 2025: 25%), tobacco use (2020: 15%, 2025: 30%), and premature mortality from NCDs (2020: 10%, 2025: 25%) and no further increase in diabetes or obesity.^39^ Based on our findings, India not only met but exceeded the mid-term target to reduce tobacco use by 15% until 2020 (Table S3 and Figs. S48–S50 in the appendix). The sustained decline since 2009^40^ might indicate the potential effectiveness of strong preventive efforts and serve as a promising example for other CVD risk factors: over the last decades, India introduced several laws and prevention programs to curb the disease burden caused by tobacco consumption (e.g., Cigarettes and Other Tobacco Products Act from 2003 and the National Tobacco Control Programme).^41^ In spite of these promising trends in tobacco consumption, our results also show that changes at the national level can mask substantial adverse trends in certain subpopulations. For instance, the absence of a national change in hypertension hides the pronounced rise among low SES people. This calls for policy targets which pay attention to potentially highly vulnerable groups that make up a considerable part of the large Indian population.

Our study has some important limitations. First, only adults aged 15–49 years were eligible for the assessment of CVD risk factors in both survey rounds. As CVDs and most of their metabolic risk factors are positively correlated with age, our estimates are not nationally representative for adults aged 18 years and over and most likely underestimate the true burden, at least in terms of the year-specific levels, and are best interpreted as being relevant for younger to middle-aged adults. However, since these people will age over time, similar gradient changes in the elderly can be expected. Secondly, antidiabetic medication was not part of the NFHS-4 survey instruments so that we could not classify people based on their treatment status. This might result in a lower diabetes prevalence but we think the difference would be marginal (most people who receive treatment will also report to have been diagnosed with diabetes and the share that actually receives treatment is low in India^42^) and that a substantial effect on trends over time is unlikely. Third, the NFHSs only measured random blood glucose and on a single occasion. While this is not the most precise diagnostic test for diabetes,^43^ misclassification is unlikely to differ across survey rounds or to be substantially correlated with SES. We also find that our results are essentially unchanged when classifying the diabetes status of participants who fasted at the time of the survey (self-reported fasting for more than 8 h) based on the threshold ≥126 $\frac{mg}{dL}$ instead of ≥200 $\frac{mg}{dL}$ (Figs. S62–S64 in the appendix). Fourth, South Asians tend to develop CVDs at lower values of established risk factors compared with other ethnicities, particularly BMI.^44^, ^45^, ^46^, ^47^, ^48^, ^49^ Accordingly, the traditional overweight and obesity cutoffs do not identify all people at risk. While some studies aimed to establish Asian-specific BMI values that are risk-equivalent to the conventional obesity cutoff used for Caucasian populations, the WHO expert consultation on appropriate BMI cutoff points for Asian people concluded that these thresholds might not equally apply to all CVDs and highlighted substantial risk differences across Asian subpopulations at a certain BMI.^50^ In the absence of uncontroversial thresholds, we used the traditional overweight and obesity cutoffs in our main analysis. Nonetheless, we conducted a sensitivity analysis with two alternative ethnicity-specific cutoff points for unhealthy weight associated with i) elevated (BMI≥23 $\frac{kg}{m^{2}}$) or ii) high risk (BMI≥27.5 $\frac{kg}{m^{2}}$) which the WHO expert consultation proposed as potential trigger points for public health action. In addition to higher levels, using these definitions resulted in patterns comparable to overweight and obesity but further increased the magnitude of most gradient changes (Table S3 and Figs. S55–60 in the appendix). Fifth, the estimated changes in diabetes and hypertension may partially be driven by differential self-reporting of disease status (e.g., due to improved health literacy) or differential access to screenings across survey waves (e.g., due to the introduction of Ayushman Bharat).^51^^,^^52^ However, our sensitivity analysis with alternative diabetes and hypertension indicators solely based on biomarker measurements shows that our main conclusions regarding gradient changes are robust and that secular disease trends are likely underlying the shift of the disease burden. Sixth, about 0.6% of our sample indicated to use tobacco but did not further specify the type. We considered these survey participants as non-smokers since it was not possible to determine whether these people use smoked or smokeless variants of tobacco. Finally, as NFHS-5 fieldwork was partly conducted during the COVID-19 pandemic, lockdowns might have contributed to the estimated trends, particularly the changes in overweight and tobacco consumption. However, the disproportionate increases among disadvantaged populations are also apparent in the subsample of states with pre-pandemic NFHS-5 fieldwork (Figs. S88–S90 in the appendix) which suggests that our findings are unlikely to be driven by the pandemic.

Using nationally representative data from two survey rounds with reliable biomarker measurements, our analysis provides a comprehensive picture of considerable changes in the social patterning of all major CVD risk factors. Due to disproportionate absolute and relative increases in the prevalence of unhealthy weight, hypertension, and diabetes among Indians with low SES and in rural areas since 2015–16, the socioeconomic and geographic gradients in the country substantially changed to the detriment of less-educated, relatively poor, and rural subpopulations; CVD risk factors are now far more widespread throughout the population. To prevent a severe double burden of infectious diseases and NCDs among vulnerable subpopulations and to achieve a sustainable reduction in CVD mortality, CVD prevention programs that ensure to reach marginalized population groups and disproportionately affected regions will be essential.

## Contributors

PG and NS conceived of the study idea. SW, NS, and PG developed the methodological approach. Supervised by NS and PG, SW conducted the data analysis and wrote the initial manuscript. Both SW and NS directly accessed and verified the reported data. PG and NS acquired the necessary funding. All authors discussed the interpretation of results, contributed to revising the manuscript, and accept responsibility to submit for publication. NS and PG are the joint senior authors of this study.

## Data sharing statement

The data used for the analysis is publicly accessible (https://dhsprogram.com/).

## Editor note

The Lancet Group takes a neutral position with respect to territorial claims in published maps and institutional affiliations.

## Declaration of interests

Outside the scope of the submitted manuscripts, Mohammed K. Ali received personal advisory board fees from Eli Lilly and Bayer and Emory University received a grant from Merck & Co. The other authors declare no conflict of interest.
