# Supplementary material for: Changing socioeconomic and geographic gradients in cardiovascular disease risk factors among Indians aged 15–49 years – evidence from nationally representative household surveys
Source: Lancet Reg Health Southeast Asia. 2023 Apr 14;12:100188. doi: 10.1016/j.lansea.2023.100188 (PMC10305936; doi:10.1016/j.lansea.2023.100188)
Supplement: Supplementary material [file mmc1.pdf]

Supplementary material

Changing socioeconomic and geographic gradients in CVD risk factors among Indians aged 15-49 years – Evidence from nationally representative household surveys

Sarah Wetzel[1], Pascal Geldsetzer[2,3*,] Sneha Sarah Mani[4], Aashish Gupta[5], Kavita Singh[1,6], Mohammed K. Ali[7,8], Dorairaj Prabhakaran[9,10], Nikhil Tandon[11], Nikkil Sudharsanan[1,12*]

[1] Heidelberg Institute of Global Health, Heidelberg University, Heidelberg, Germany

[2] Division of Primary Care and Population Health, Department of Medicine, Stanford University, Stanford, CA, USA

[3] Chan Zuckerberg Biohub, San Francisco, CA, USA

[4] Graduate Group in Demography, University of Pennsylvania, PA, USA

[5] Harvard Center for Population and Development Studies, Harvard T.H. Chan School of Public Health, Harvard University, MA, USA

[6] Centre for Chronic Conditions and Injuries, Public Health Foundation of India, Gurugram, Haryana, India

[7] Hubert Department of Global Health, Rollins School of Public Health, Emory University, Atlanta, GA, USA

[8] Department of Family and Preventive Medicine, School of Medicine, Emory University, Atlanta, GA, USA

[9] Executive Director, Centre for Chronic Disease Control, India

[10] Distinguished Professor of Public Health, Public Health Foundation of India, India

[11] Head of the department of Endocrinology and Metabolism, All India Institute of Medical Sciences, India

[12] Professorship of Behavioral Science for Disease Prevention and Health Care, Technical University of Munich, Munich, Germany

* Joint senior authors

<u>Corresponding author:</u>

Name:	Sarah Wetzel, MA
Address:	Heidelberg Institute of Global Health, Heidelberg University<br>Im Neuenheimer Feld 130.3<br>69120 Heidelberg, Germany
Email:	sarah.wetzel@uni-heidelberg.de
Tel.:	+49 6221 56-5344
Fax.:	+49 6221 56-5948
ORCID:	0000-0002-7376-1770

Contents

Sampling procedure 4
Definitions 5
Age-standardized and weighted sample characteristics 6
Trends at the national level 7
Estimates using the time-specific age-distribution 7
Age-standardized estimates 7
Results stratified by socioeconomic group or place of residence 8
Estimates using the time-specific age-distribution 8
Smoking 8
Cigarette smoking 9
Tobacco consumption 10
Overweight 11
Obesity 12
BMI ≥ 23 kg/m^2 13
BMI ≥ 27.5 kg/m^2 15
Diabetes 17
Diabetes (alternative definition) 18
High blood glucose 19
Self-reported diabetes 20
Hypertension 21
High blood pressure 22
Told to have high blood pressure 23
Age-standardized 24
Smoking 24
Cigarette smoking 25
Tobacco consumption 26
Overweight 27
Obesity 28
BMI ≥ 23 kg/m^2 29
BMI ≥ 27.5 kg/m^2 31
Diabetes 33
Diabetes (alternative definition) 34
High blood glucose 35
Self-reported diabetes 36
Hypertension 37
High blood pressure 38
Told to have high blood pressure 39
Results stratified by region 40
NFHS-4 40
Absolute changes 41
Relative changes 42

Results stratified by regional level of development 43
NFHS-4 43
Absolute changes 44
Relative changes 45
Results stratified by sex and socioeconomic status or place of residence 46
Women 46
Men 47
Results within the subsample of states with pre-pandemic fieldwork 48
Regression coefficients 50
Models to estimate national trends 50
Models to estimate group-specific trends 50
Level of education 50
Wealth quintile 51
Place of residence 51
Regions 52
EAG membership 52
References 53

Sampling procedure

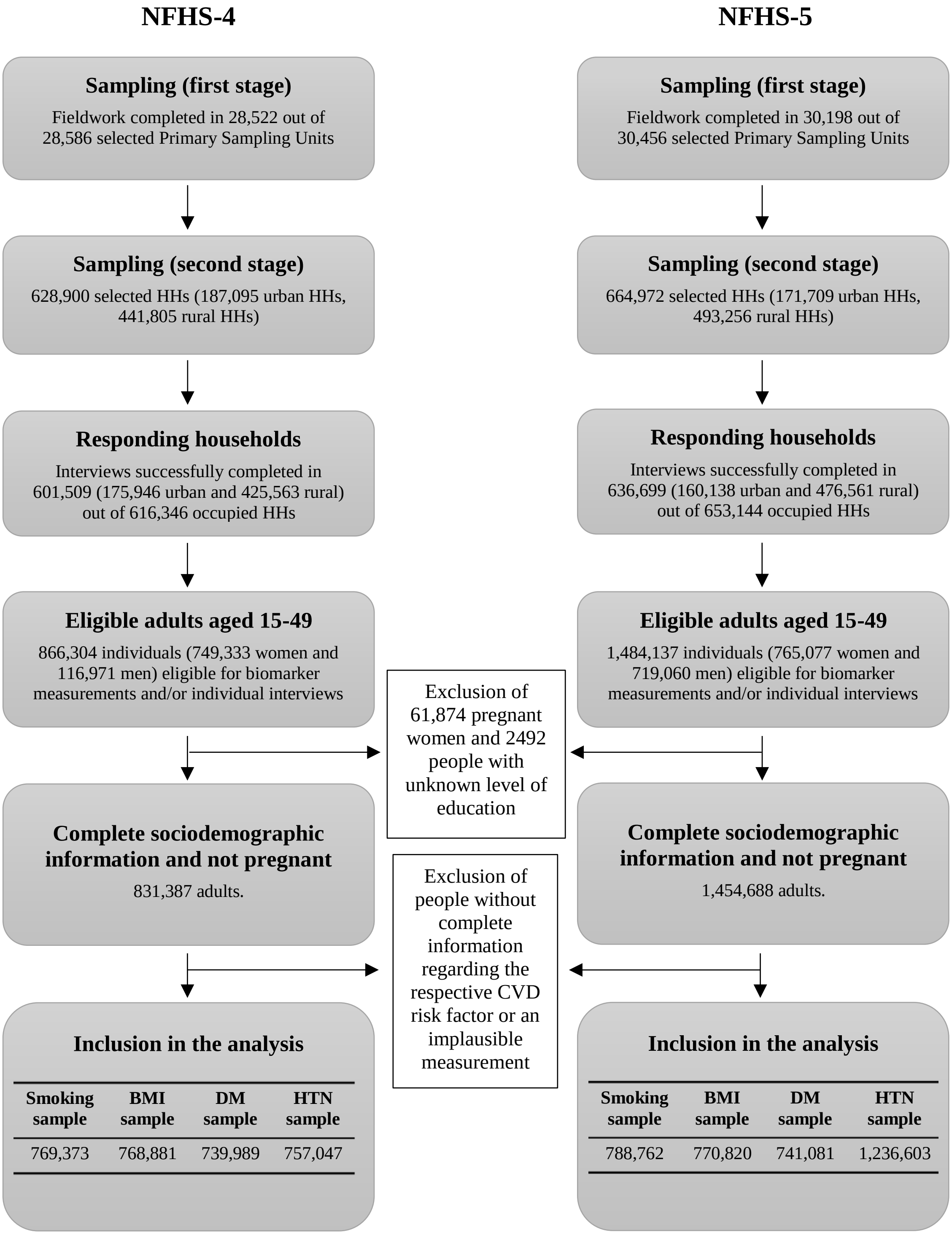

Figure S1. Flowchart of the sampling procedure
HH=household, BMI=body mass index, DM=diabetes, HTN=hypertension. In the NFHS-5, adults aged 15 years and older were eligible for glucose tests as well as for blood pressure measurements and asked about hypertension diagnoses or treatments while all other mentioned CVD-related items only covered men aged 15-54 in a representative subsample of 15% of households (state module) and women aged 15-49 years.

Definitions

the estimated changes in diabetes and hypertension may partially be driven by differential self-reporting of disease status and changing screening behavior or access to care. In addition, the share of the population that had been diagnosed with hypertension followed a strong and potentially unrealistic downward trend in a few states which might indicate potential data quality issues with regard to this variable. To demonstrate the robustness of our findings and to differentiate the contribution of secular disease trends, trends in self-reporting, and the combined effect of the two, we present results for three different hypertension indicators below: i) an indicator for an average systolic blood pressure greater than or equal to 140mmHg or an average diastolic blood pressure greater than or equal to 90mmHg, ii) an indicator for being told to have high blood pressure by a clinician on two different occasions, and iii) the combined indicator defined as previously (high blood pressure, told to have high blood pressure, or current medication). Similarly, we show trends for four different diabetes indicators: i) an indicator for a random plasma glucose concentration $\geq 200\frac{mg}{dL}$, ii) an indicator for self-reported diabetes status, iii) in indicator for self-reported diabetes status, a random venous plasma glucose concentration $\geq 126\frac{mg}{dL}$ if fasting [not eating or drinking anything besides water for more than 8 hours], or $\geq 200\frac{mg}{dL}$ if non-fasted, and iv) the combined indicator defined as previously (high blood glucose or self-reported diabetes). We measured cigarette smoking and tobacco consumption (any type, including smokeless variants) as binary indicators based on self-report. All other variables are defined as described in the paper.

To assess the contribution of aging, we compare age-standardized estimates (direct age-standardization based on the WHO standard population [1]) with the estimates using the actual time-specific age distribution of the Indian population. To ensure that our data was representative of the age distribution of the Indian population aged 15-49 after applying the exclusion criteria, we adapted the household weights based on the Indian age distribution in the respective survey year according to the United Nations World Population Prospects population estimates (UNWPP). [2] However, mean age among adults aged 15-49 increased by less than 0.4 years between survey rounds. As a result, age standardization based on the WHO standard population only had a minor impact on our estimates and the conclusions regarding the trends in CVD gradients hold true for both crude and age-standardized estimates.

Age-standardized and weighted sample characteristics

	Smoking sample		Overweight sample		Diabetes sample		Hypertension sample	
	**NFHS-4** *N = 769,373*	**NFHS-5** *N = 788,762*	**NFHS-4** *N = 768,881*	**NFHS-5** *N = 770,820*	**NFHS-4** *N = 740,078*	**NFHS-5** *N = 741,096*	**NFHS-4** *N = 757,047*	**NFHS-5** *N = 1,236,733*
**Sex**								
Male	388,960 (51%)	369,217 (47%)	387,513 (50%)	357,005 (46%)	372,290 (50%)	341,803 (46%)	381,198 (50%)	572,334 (46%)
Female	380,413 (49%)	419,545 (53%)	381,368 (50%)	413,815 (54%)	367,699 (50%)	399,278 (54%)	375,849 (50%)	664,269 (54%)
**Age group**								
15-19 years	125,295 (16%)	128,452 (16%)	125,215 (16%)	125,531 (16%)	120,510 (16%)	120,687 (16%)	123,288 (16%)	201,385 (16%)
20-24 years	121,597 (16%)	124,661 (16%)	121,519 (16%)	121,825 (16%)	116,953 (16%)	117,125 (16%)	119,649 (16%)	195,441 (16%)
25-29 years	117,307 (15%)	120,263 (15%)	117,232 (15%)	117,527 (15%)	112,827 (15%)	112,993 (15%)	115,427 (15%)	188,546 (15%)
30-34 years	112,573 (15%)	115,410 (15%)	112,501 (15%)	112,785 (15%)	108,274 (15%)	108,434 (15%)	110,770 (15%)	180,937 (15%)
35-39 years	105,768 (14%)	108,434 (14%)	105,701 (14%)	105,967 (14%)	101,729 (14%)	101,879 (14%)	104,074 (14%)	170,000 (14%)
40-44 years	97,484 (13%)	99,941 (13%)	97,422 (13%)	97,668 (13%)	93,761 (13%)	93,900 (13%)	95,923 (13%)	156,686 (13%)
45-49 years	89,348 (12%)	91,600 (12%)	89,291 (12%)	89,516 (12%)	85,936 (12%)	86,063 (12%)	87,917 (12%)	143,609 (12%)
**Level of education**								
No education	149,087 (19%)	126,440 (16%)	149,908 (19%)	125,129 (16%)	144,395 (20%)	119,956 (16%)	148,165 (20%)	202,226 (16%)
Incomplete primary	50,361 (6.5%)	46,325 (5.9%)	50,598 (6.6%)	45,813 (5.9%)	48,478 (6.6%)	43,837 (5.9%)	49,754 (6.6%)	71,723 (5.8%)
Complete primary	49,271 (6.4%)	50,177 (6.4%)	49,643 (6.5%)	49,376 (6.4%)	47,673 (6.4%)	47,456 (6.4%)	49,182 (6.5%)	79,353 (6.4%)
Incomplete secondary	326,082 (42%)	338,876 (43%)	326,072 (42%)	332,354 (43%)	313,928 (42%)	320,043 (43%)	320,837 (42%)	530,699 (43%)
Complete secondary	77,454 (10%)	88,790 (11%)	77,521 (10%)	86,297 (11%)	74,407 (10%)	83,235 (11%)	76,306 (10%)	139,372 (11%)
Higher than secondary	117,117 (15%)	138,155 (18%)	115,138 (15%)	131,851 (17%)	111,108 (15%)	126,554 (17%)	112,804 (15%)	213,229 (17%)
**Wealth quintile**								
Poorest	125,386 (16%)	140,799 (18%)	127,006 (17%)	139,930 (18%)	121,807 (16%)	133,423 (18%)	125,611 (17%)	224,477 (18%)
Poorer	148,512 (19%)	158,592 (20%)	150,247 (20%)	157,186 (20%)	144,425 (20%)	150,996 (20%)	148,450 (20%)	248,577 (20%)
Middle	161,386 (21%)	163,912 (21%)	161,670 (21%)	161,722 (21%)	155,607 (21%)	156,123 (21%)	159,353 (21%)	260,830 (21%)
Richer	166,149 (22%)	165,700 (21%)	164,990 (21%)	161,800 (21%)	159,154 (22%)	155,800 (21%)	161,975 (21%)	261,963 (21%)
Richest	167,941 (22%)	159,759 (20%)	164,967 (21%)	150,182 (19%)	158,997 (21%)	144,739 (20%)	161,658 (21%)	240,755 (19%)
**Place of residence**								
Urban	273,751 (36%)	258,811 (33%)	268,525 (35%)	245,630 (32%)	258,459 (35%)	236,044 (32%)	263,049 (35%)	396,738 (32%)
Rural	495,622 (64%)	529,951 (67%)	500,356 (65%)	525,190 (68%)	481,530 (65%)	505,037 (68%)	493,998 (65%)	839,865 (68%)

Table S1. Age-standardized (WHO standard population) and weighted sample characteristics

Trends at the national level
Estimates using the time-specific age-distribution

Outcome	NFHS-4	NFHS-5	Absolute change (percentage points)	Relative change (%)
Smoking	12.3 [12; 12.6]	9.3 [9; 9.6]	-3 [-3.5; -2.6]	-24.7 [-27.8; -21.5]
Cigarette smoking	6.9 [6.7; 7.2]	5.9 [5.7; 6.2]	-1 [-1.3; -0.6]	-14.2 [-18.8; -9.5]
Tobacco Consumption	26 [25.5; 26.4]	20.9 [20.5; 21.3]	-5 [-5.6; -4.4]	-19.3 [-21.3; -17.3]
Overweight	19.3 [19; 19.6]	23 [22.8; 23.3]	3.7 [3.3; 4.1]	19.2 [16.8; 21.6]
Obesity	3.9 [3.8; 4]	5.2 [5; 5.3]	1.2 [1.1; 1.4]	31.2 [26.4; 36.2]
BMI $\geq$ 23 kg/m^2	33.5 [33.1; 33.9]	38.5 [38.2; 38.9]	5.0 [4.5; 5.5]	14.9 [13.4; 16.5]
BMI $\geq$ 27.5 kg/m^2	9.0 [8.8; 9.2]	11.1 [10.9; 11.3]	2.1 [1.8; 2.4]	23.4 [19.9; 27.0]
Diabetes	2.6 [2.5; 2.7]	2.9 [2.8; 3]	0.3 [0.1; 0.4]	10.8 [5.2; 16.6]
Diabetes (alternative definition)	2.7 [2.6; 2.8]	3 [2.9; 3.1]	0.2 [0.1; 0.4]	9.4 [3.9; 15.1]
High blood glucose	1.4 [1.3; 1.5]	1.6 [1.5; 1.7]	0.2 [0.1; 0.3]	13.8 [6.6; 21.5]
Self-reported diabetes	1.7 [1.6; 1.8]	1.9 [1.8; 2]	0.2 [0.1; 0.3]	12.9 [5.6; 20.7]
Hypertension	17.1 [16.9; 17.4]	16.3 [16.2; 16.5]	-0.8 [-1.2; -0.5]	-4.8 [-6.7; -3]
High blood pressure	10.9 [10.7; 11.1]	12 [11.9; 12.2]	1.2 [1; 1.4]	10.8 [8.7; 13.1]
Told to have high blood pressure	7.7 [7.4; 8]	5.4 [5.3; 5.5]	-2.3 [-2.6; -2]	-29.7 [-32.5; -26.8]

Table S2. Trends in CVD risk factors at the national level

Age-standardized estimates

Outcome	NFHS-4	NFHS-5	Absolute change (percentage points)	Relative change (%)
Cigarette smoking	7 [6.7; 7.2]	6 [5.7; 6.2]	-1 [-1.4; -0.7]	-14.6 [-19.1; -9.8]
Tobacco consumption	26.5 [26.1; 26.9]	21.1 [20.8; 21.6]	-5.3 [-5.9; -4.8]	-20.1 [-22.1; -18.2]
Obesity	4.1 [4; 4.2]	5.2 [5.1; 5.4]	1.2 [1; 1.3]	28.4 [23.7; 33.1]
BMI $\geq$ 23 kg/m^2	34.4 [34.0; 34.7]	38.9 [38.6; 39.3]	4.6 [4.1; 5.1]	13.3 [11.7; 14.8]
BMI $\geq$ 27.5 kg/m^2	9.3 [9.1; 9.5]	11.3 [11.1; 11.5]	1.9 [1.7; 2.2]	20.7 [17.4; 24.2]
Diabetes (alternative definition)	2.9 [2.8; 3]	3.1 [3; 3.2]	0.2 [0; 0.3]	6.1 [0.8; 11.6]
High blood glucose	1.5 [1.4; 1.6]	1.6 [1.6; 1.7]	0.1 [0; 0.2]	9.8 [3; 17.2]
Self-reported diabetes	1.8 [1.7; 1.9]	2 [1.9; 2]	0.2 [0; 0.3]	9.5 [2.5; 17]
High blood pressure	11.4 [11.2; 11.6]	12.3 [12.2; 12.5]	0.9 [0.7; 1.2]	8.3 [6.2; 10.4]
Told to have high blood pressure	8 [7.7; 8.2]	5.5 [5.4; 5.6]	-2.4 [-2.7; -2.1]	-30.5 [-33.2; -27.7]

Table S3. Age-standardized trends in CVD risk factors at the national level

Results stratified by socioeconomic group or place of residence
Estimates using the time-specific age-distribution
Smoking

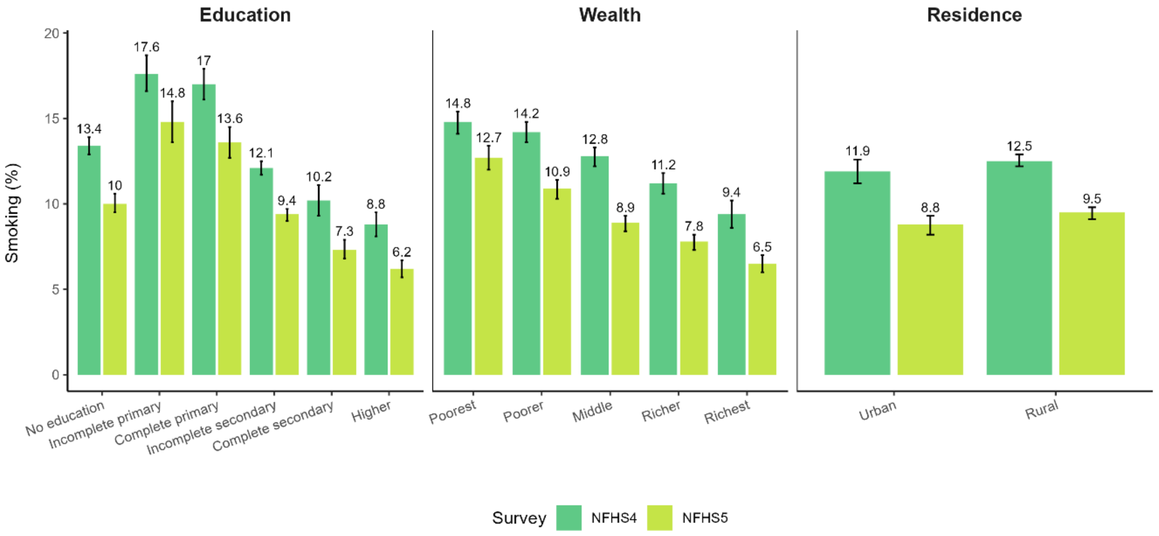

Figure S2. Prevalence of smoking among adults aged 15-49 across subpopulations in each survey round

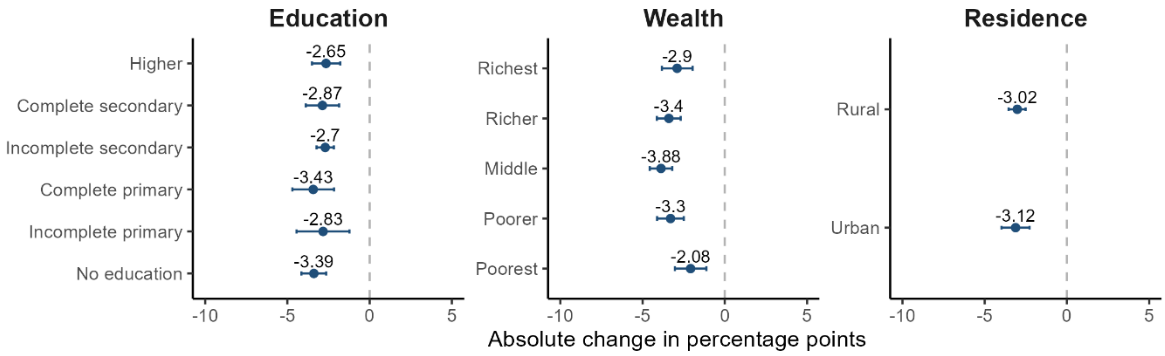

Figure S3. Absolute changes in the prevalence of smoking among adults aged 15-49 across subpopulations

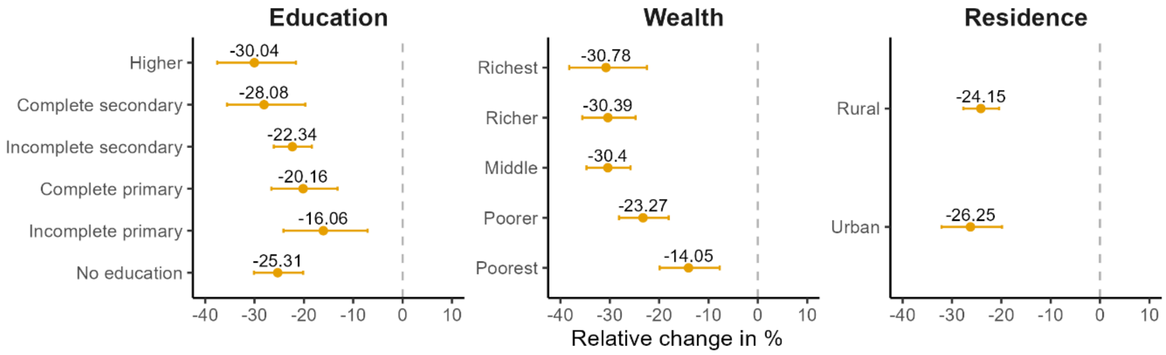

Figure S4. Relative changes in the prevalence of smoking among adults aged 15-49 across subpopulations

Cigarette smoking

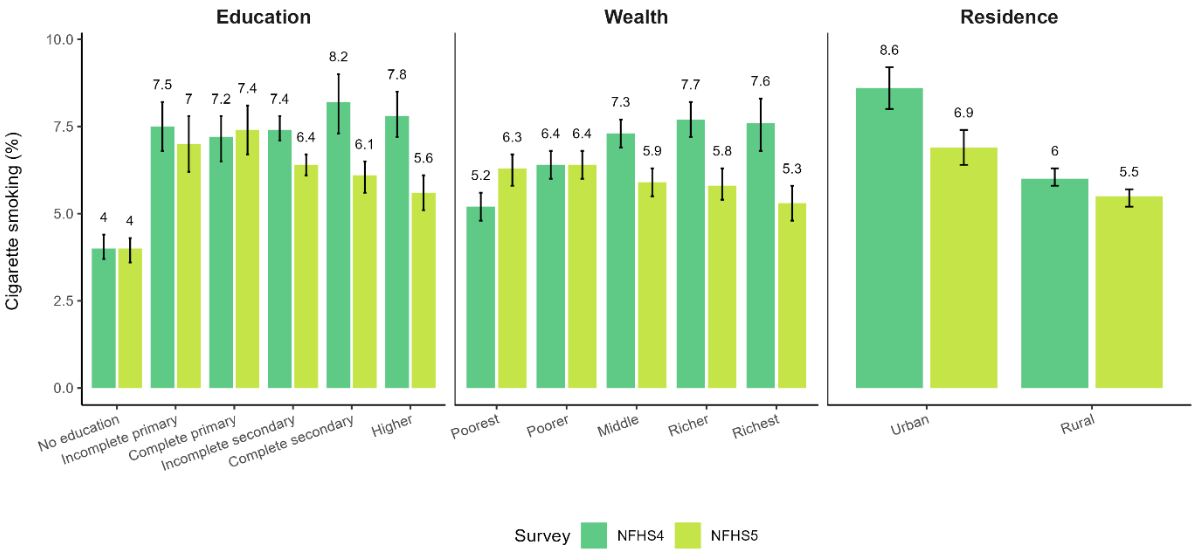

Figure S5. Prevalence of cigarette smoking among adults aged 15-49 across subpopulations in each survey round

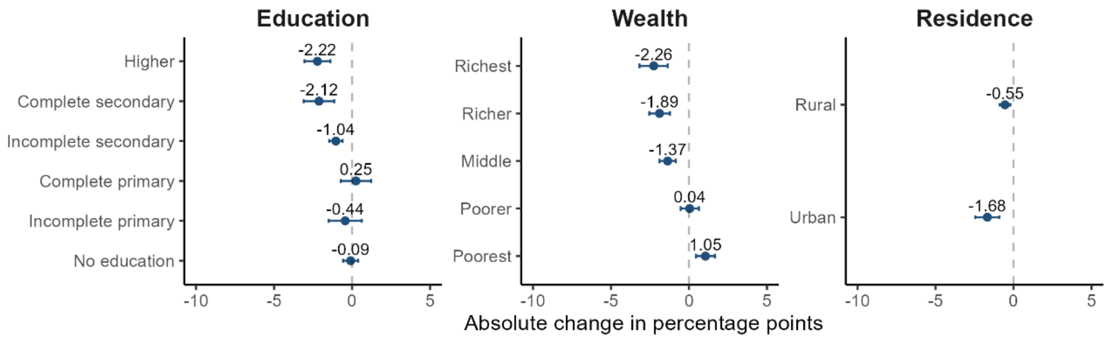

Figure S6. Absolute changes in the prevalence of cigarette smoking among adults aged 15-49 across subpopulations

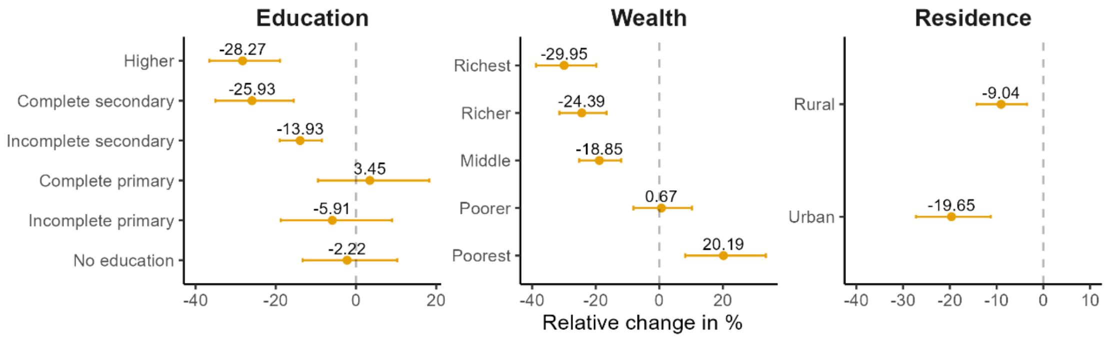

Figure S7. Relative changes in the prevalence of cigarette smoking among adults aged 15-49 across subpopulations

Tobacco consumption

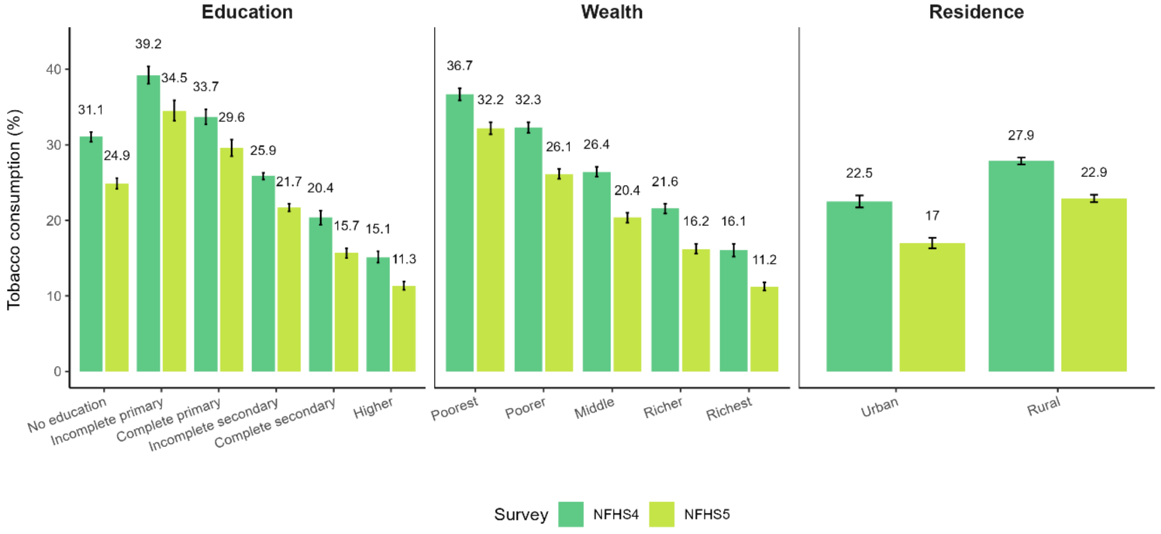

Figure S8. Prevalence of tobacco consumption among adults aged 15-49 across subpopulations in each survey round

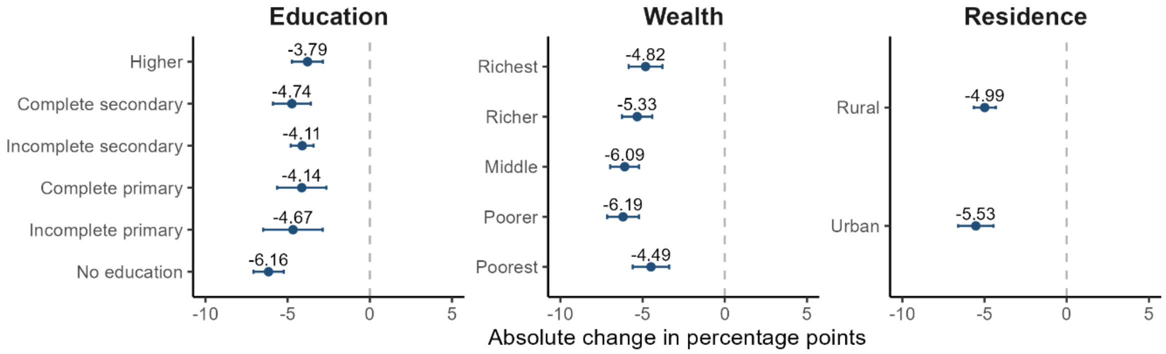

Figure S9. Absolute changes in the prevalence of tobacco consumption among adults aged 15-49 across subpopulations

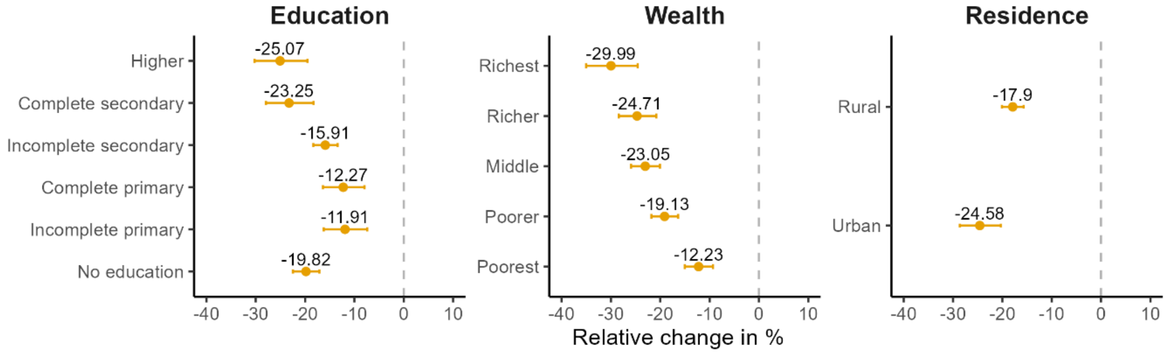

Figure S10. Relative changes in the prevalence of tobacco consumption among adults aged 15-49 across subpopulations

Overweight

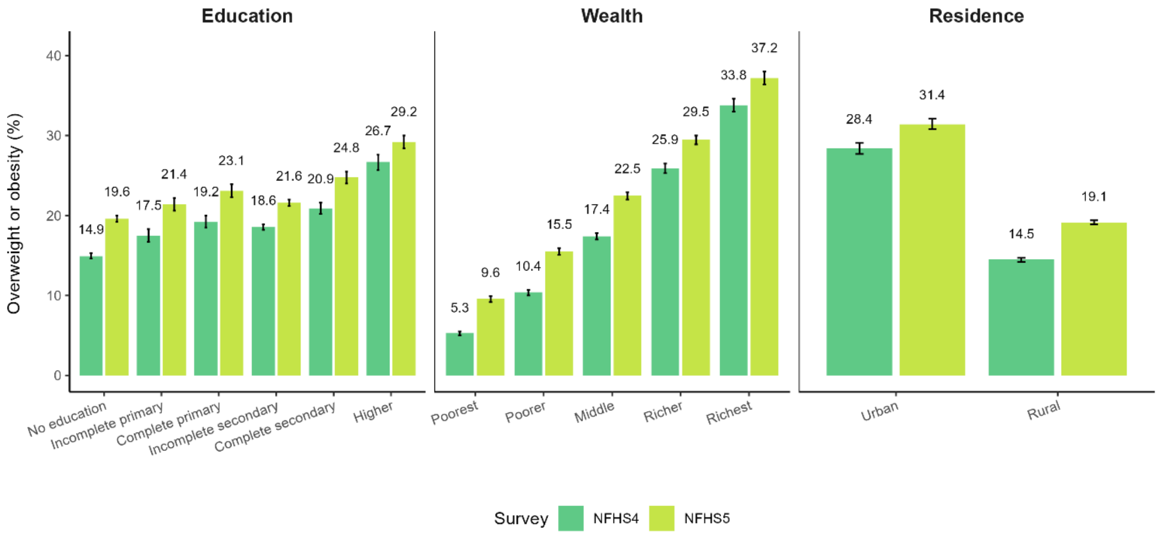

Figure S11. Prevalence of overweight or obesity among adults aged 15-49 across subpopulations in each survey round

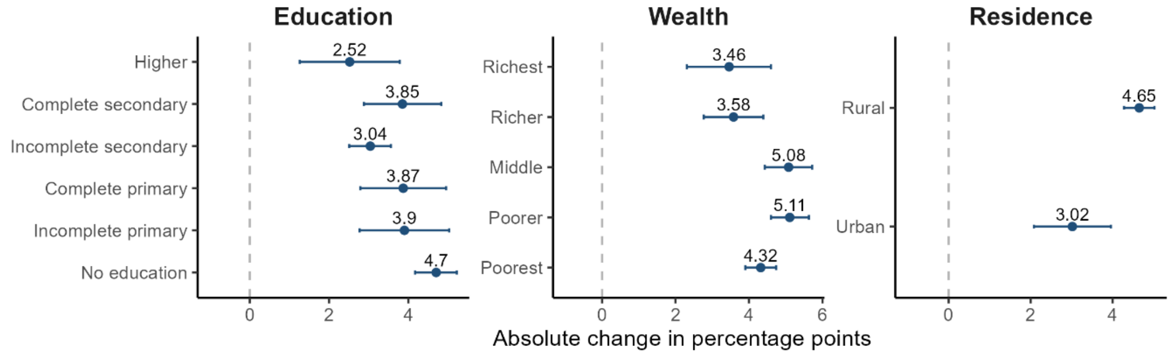

Figure S12. Absolute changes in the prevalence of overweight or obesity among adults aged 15-49 across subpopulations

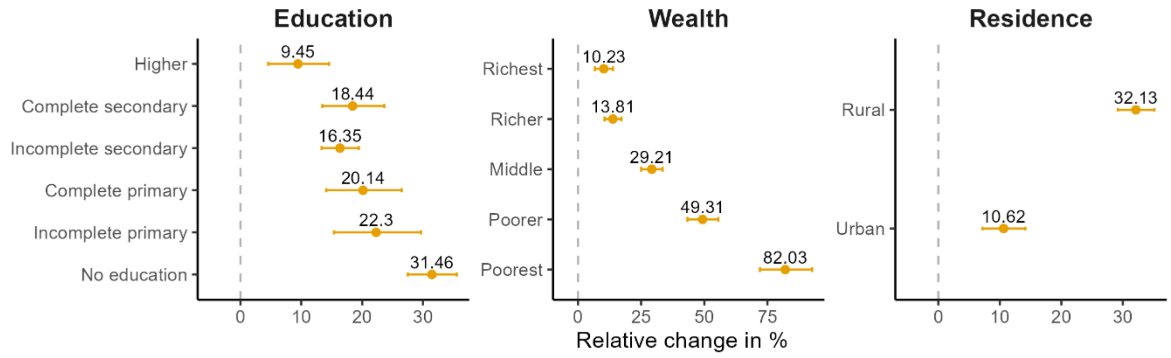

Figure S13. Relative changes in the prevalence of overweight or obesity among adults aged 15-49 across subpopulations

Obesity

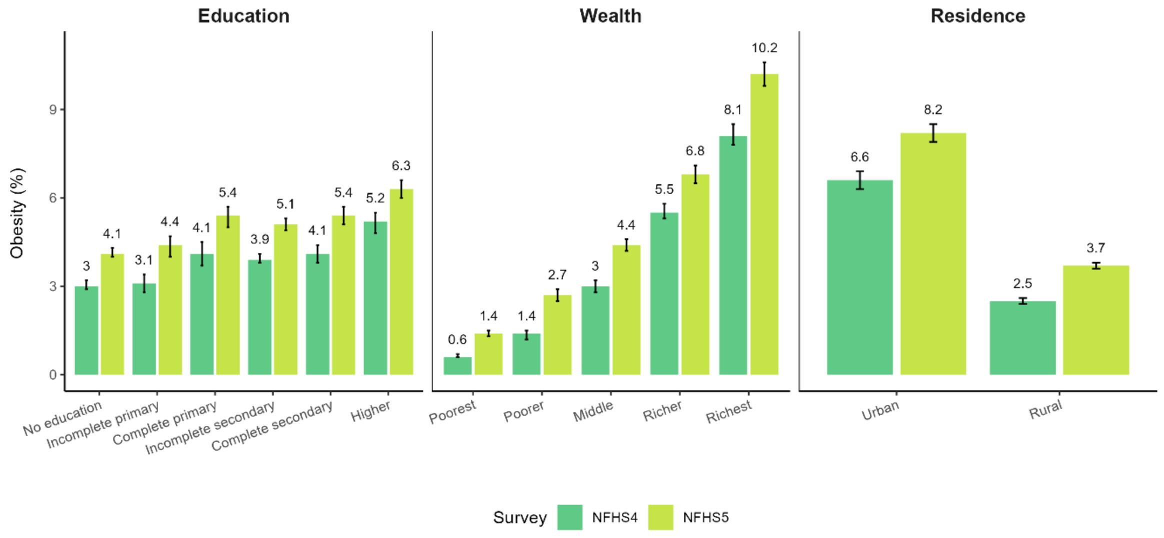

Figure S14. Prevalence of obesity among adults aged 15-49 across subpopulations in each survey round

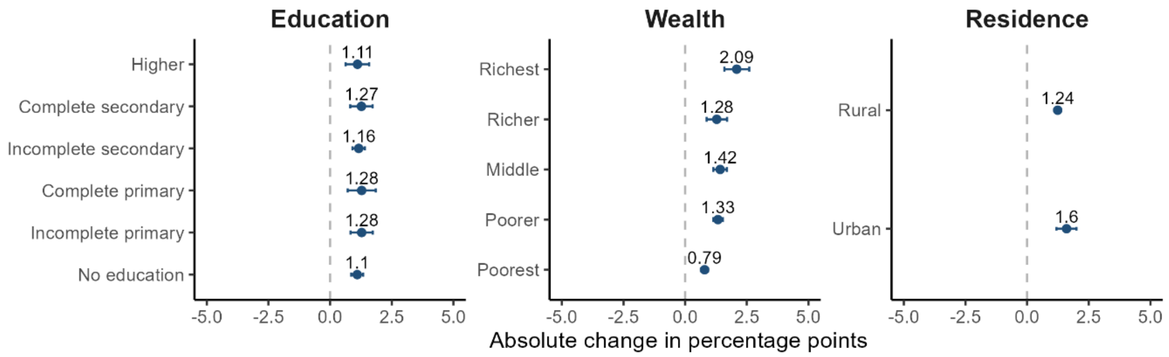

Figure S15. Absolute changes in the prevalence of obesity among adults aged 15-49 across subpopulations

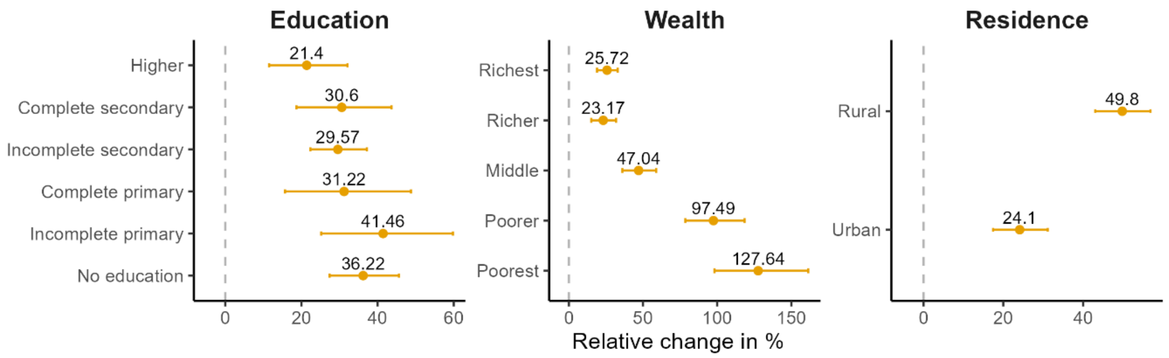

Figure S16. Relative changes in the prevalence of obesity among adults aged 15-49 across subpopulations

BMI ≥ 23 kg/m^2

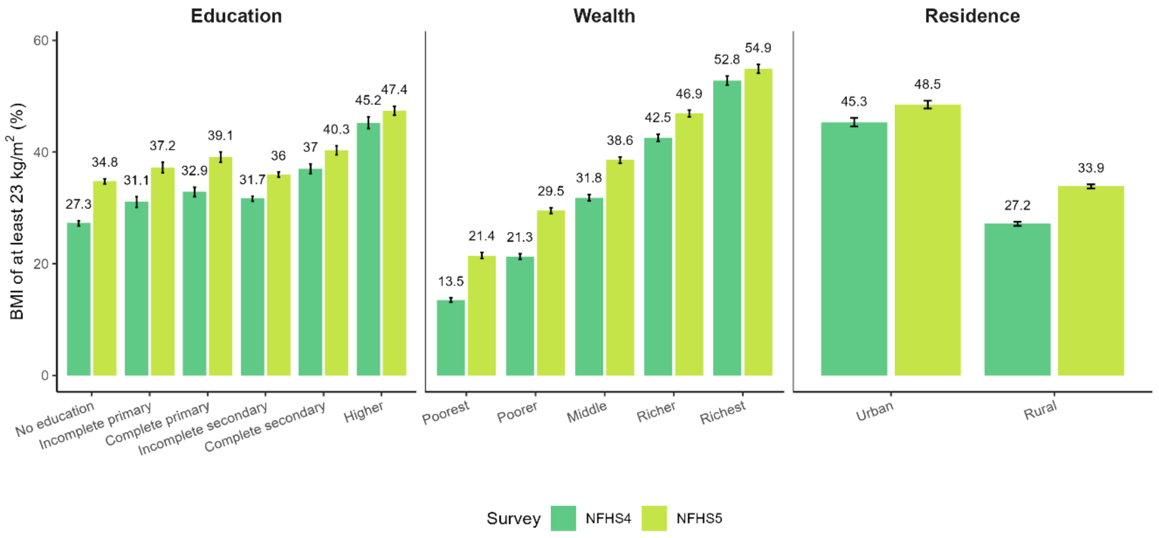

Figure S17. Prevalence of having a BMI ≥ 23 kg/m^2 among adults aged 15-49 across subpopulations in each survey round
The outcome variable is a binary indicator for having unhealthy weight with increased CVD risk based on the ethnicity-specific threshold of 23 kg/m^2 as proposed by the WHO expert consultation on appropriate BMI cutoff points for Asian people in 2002.

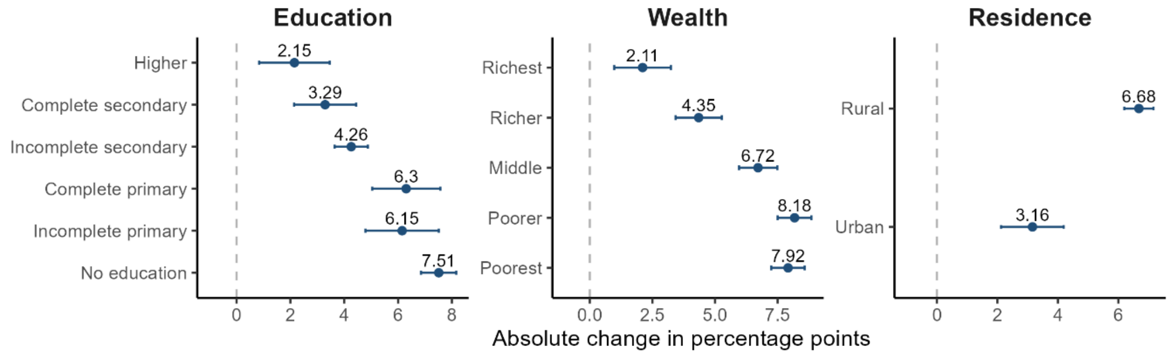

Figure S18. Absolute changes in the prevalence of having a BMI ≥ 23 kg/m^2 among adults aged 15-49 across subpopulations
The outcome variable is a binary indicator for having unhealthy weight with increased CVD risk based on the ethnicity-specific threshold of 23 kg/m^2 as proposed by the WHO expert consultation on appropriate BMI cutoff points for Asian people in 2002.

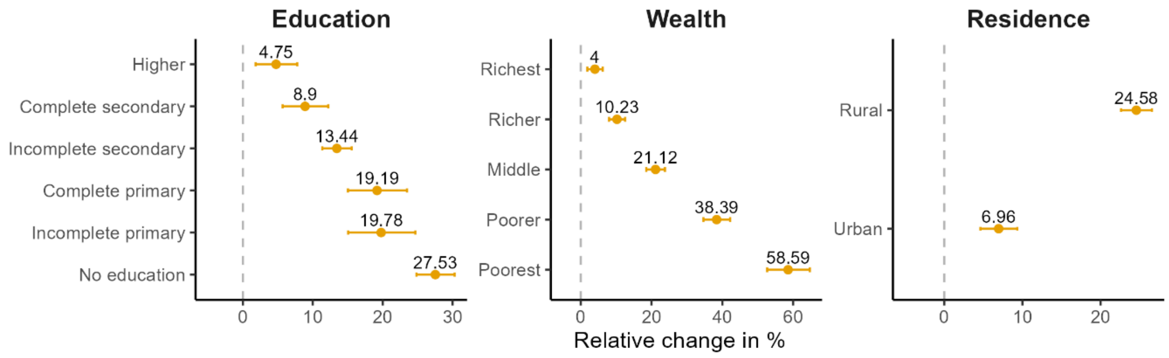

Figure S19. Relative changes in the prevalence of having a BMI ≥ 23 kg/m^2 among adults aged 15-49 across subpopulations

The outcome variable is a binary indicator for having unhealthy weight with increased CVD risk based on the ethnicity-specific threshold of 23 kg/m^2 as proposed by the WHO expert consultation on appropriate BMI cutoff points for Asian people in 2002.

BMI ≥ 27.5 kg/m²

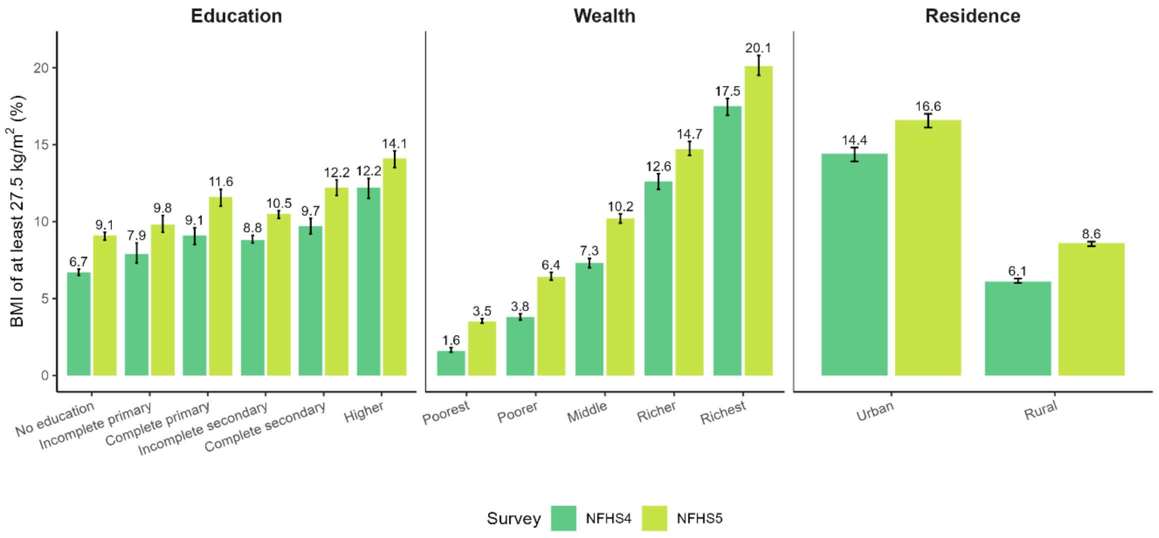

Figure S20. Prevalence of having a BMI ≥ 27.5 kg/m² among adults aged 15-49 across subpopulations in each survey round
The outcome variable is a binary indicator for having unhealthy weight with high CVD risk based on the ethnicity-specific threshold of 27.5 kg/m² as proposed by the WHO expert consultation on appropriate BMI cutoff points for Asian people in 2002.

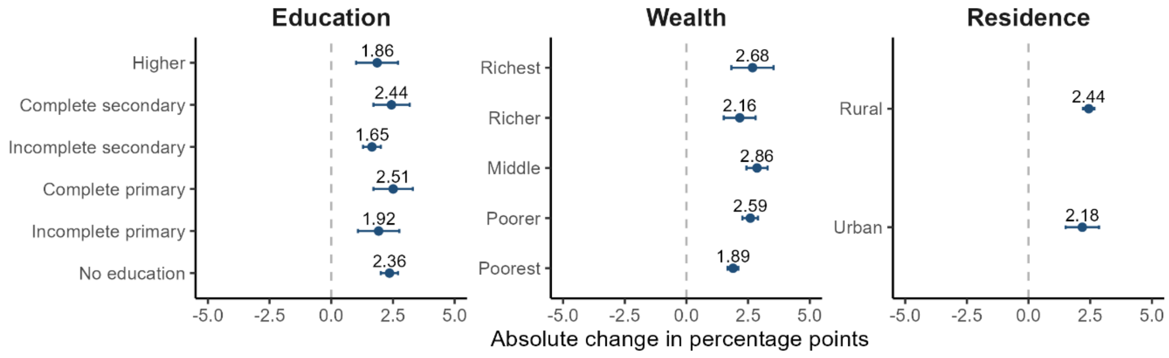

Figure S21. Absolute changes in the prevalence of having a BMI ≥ 27.5 kg/m² among adults aged 15-49 across subpopulations
The outcome variable is a binary indicator for having unhealthy weight with high CVD risk based on the ethnicity-specific threshold of 27.5 kg/m² as proposed by the WHO expert consultation on appropriate BMI cutoff points for Asian people in 2002.

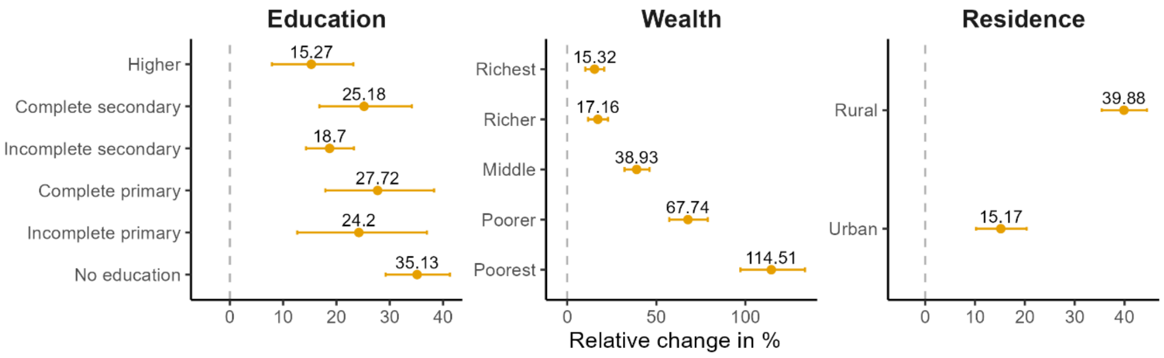

Figure S22. Relative changes in the prevalence of having a BMI ≥ 27.5 kg/m^2 among adults aged 15-49 across subpopulations

The outcome variable is a binary indicator for having unhealthy weight with high CVD risk based on the ethnicity-specific threshold of 27.5 kg/m^2 as proposed by the WHO expert consultation on appropriate BMI cutoff points for Asian people in 2002.

Diabetes

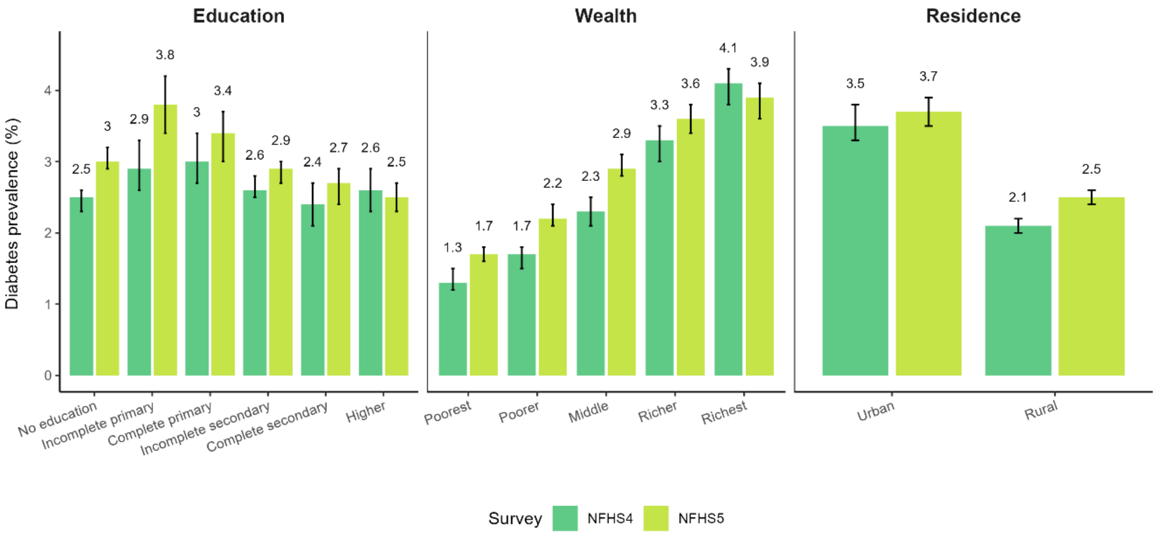

Figure S23. Prevalence of diabetes among adults aged 15-49 across subpopulations in each survey round

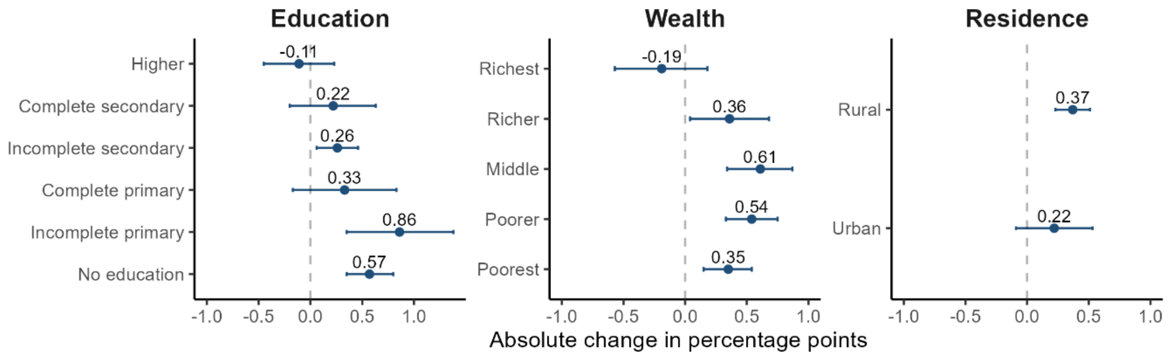

Figure S24. Absolute changes in the prevalence of diabetes among adults aged 15-49 across subpopulations

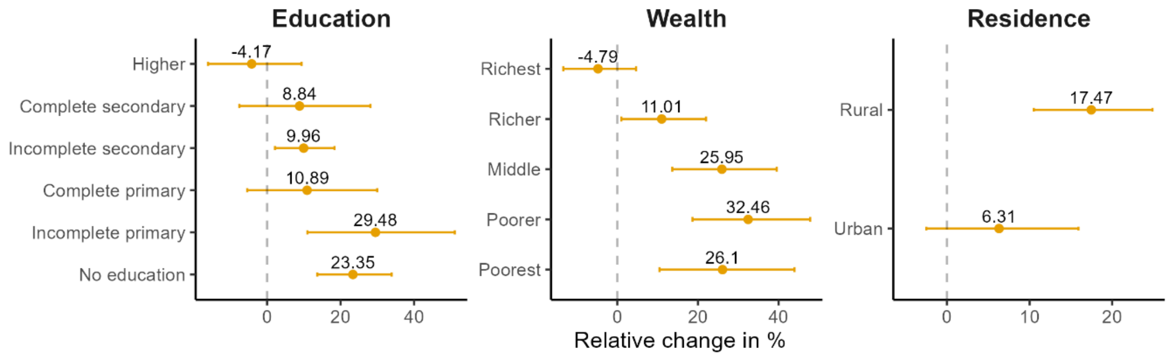

Figure S25. Relative changes in the prevalence of diabetes among adults aged 15-49 across subpopulations

Diabetes (alternative definition)

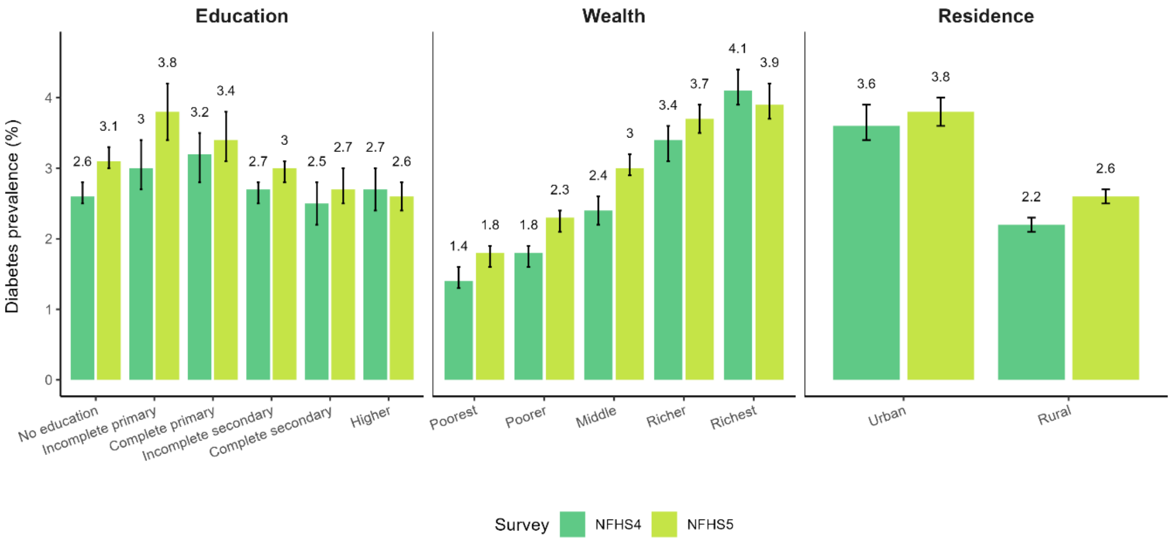

Figure S26. Prevalence of diabetes (based on an alternative definition that takes into account fasting status) among adults aged 15-49 across subpopulations in each survey round

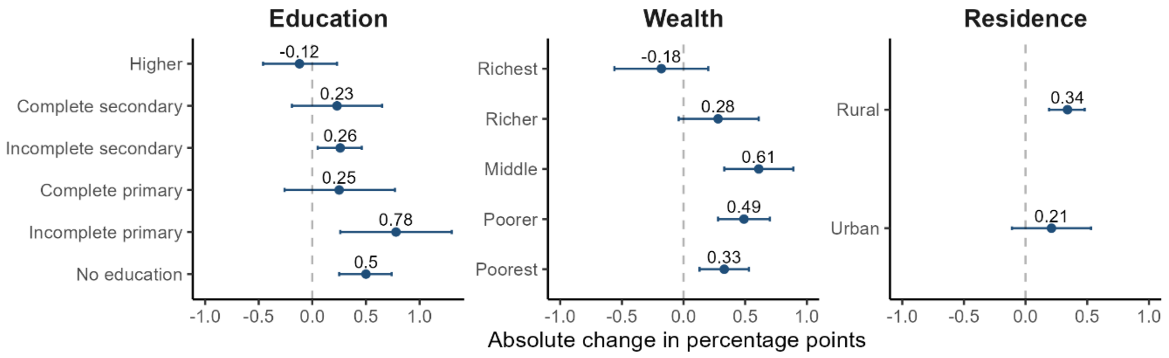

Figure S27. Absolute changes in the prevalence of diabetes (based on an alternative definition that takes into account fasting status) among adults aged 15-49 across subpopulations

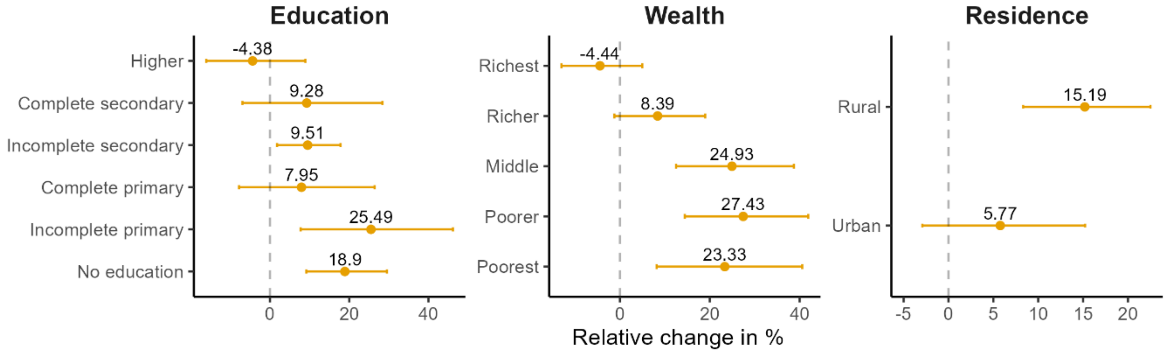

Figure S28. Relative changes in the prevalence of diabetes (based on an alternative definition that takes into account fasting status) among adults aged 15-49 across subpopulations

High blood glucose

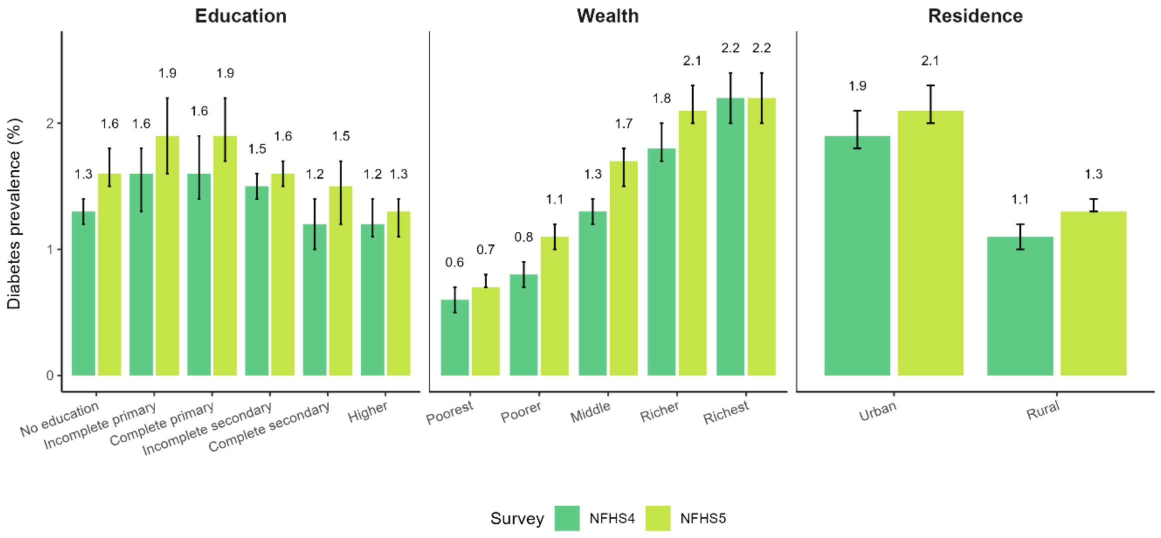

Figure S29. Prevalence of high blood glucose among adults aged 15-49 across subpopulations in each survey round

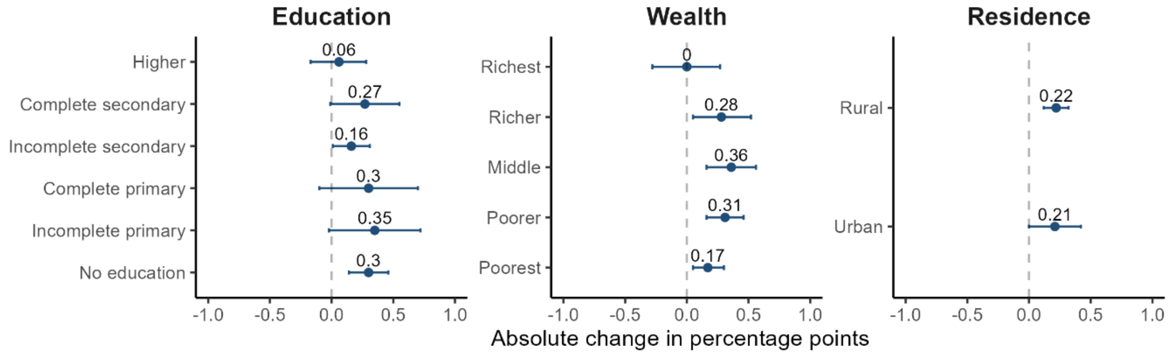

Figure S30. Absolute changes in the prevalence of high blood glucose among adults aged 15-49 across subpopulations

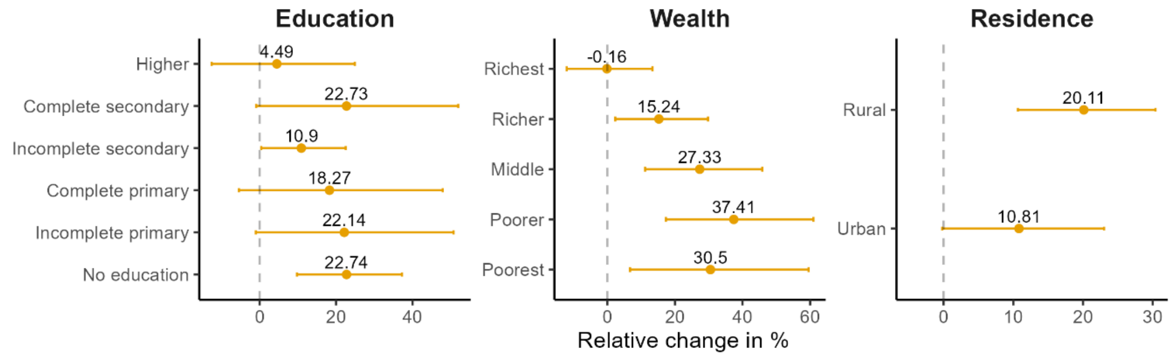

Figure S31. Relative changes in the prevalence of high blood glucose among adults aged 15-49 across subpopulations

Self-reported diabetes

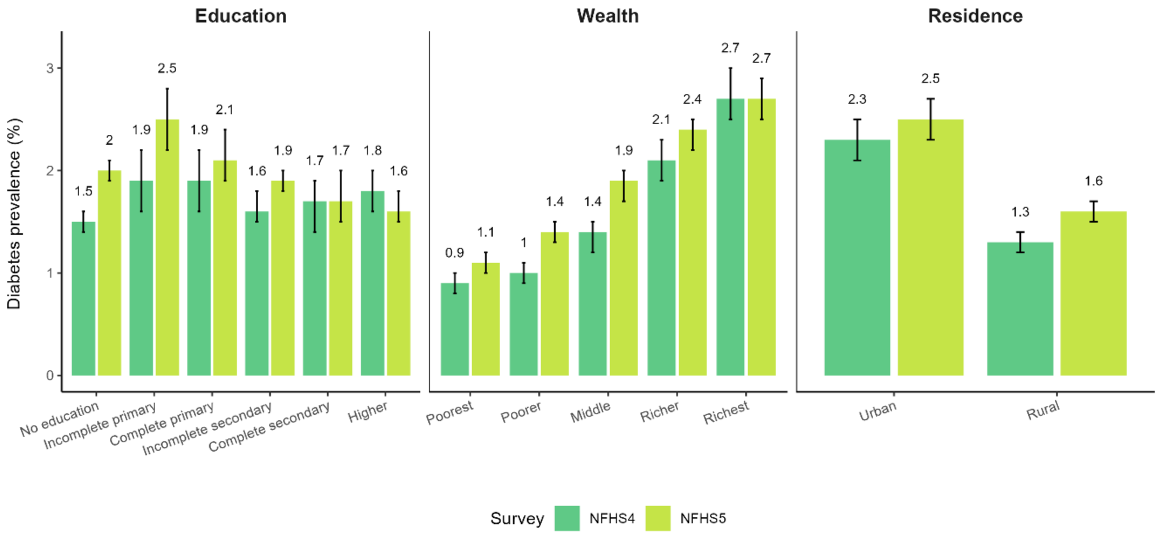

Figure S32. Prevalence of diabetes among adults aged 15-49 across subpopulations in each survey round

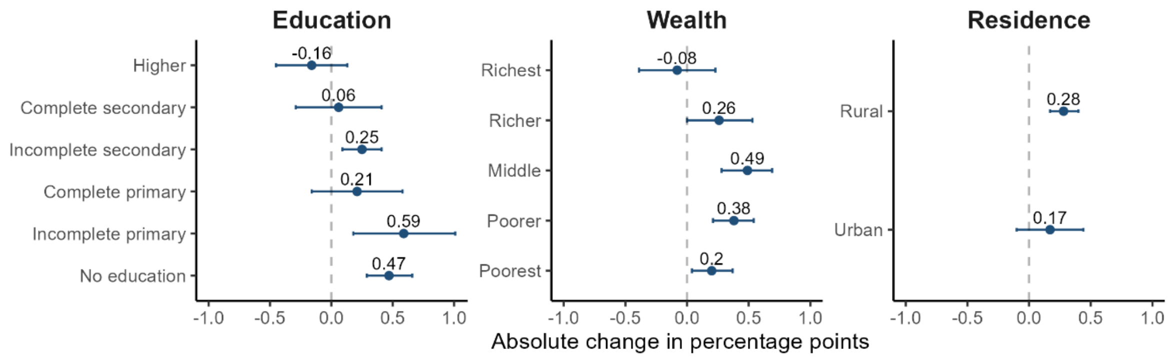

Figure S33. Absolute changes in the prevalence of self-reported diabetes among adults aged 15-49 across subpopulations

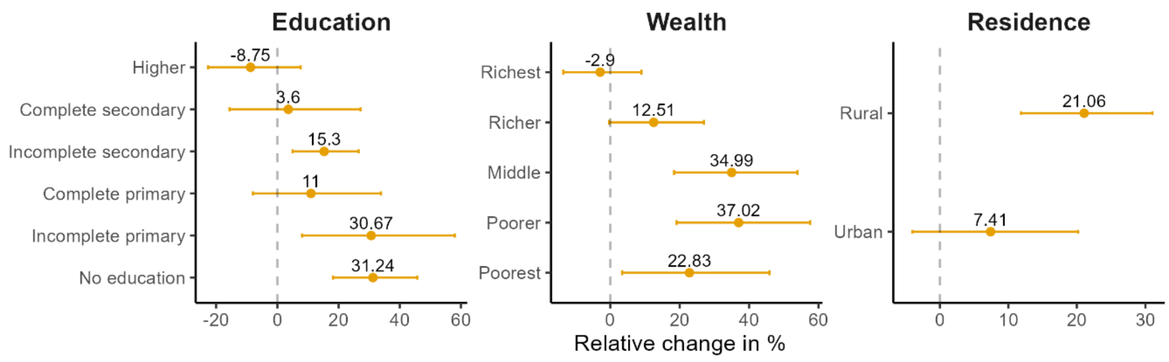

Figure S34. Relative changes in the prevalence of self-reported diabetes among adults aged 15-49 across subpopulations

Hypertension

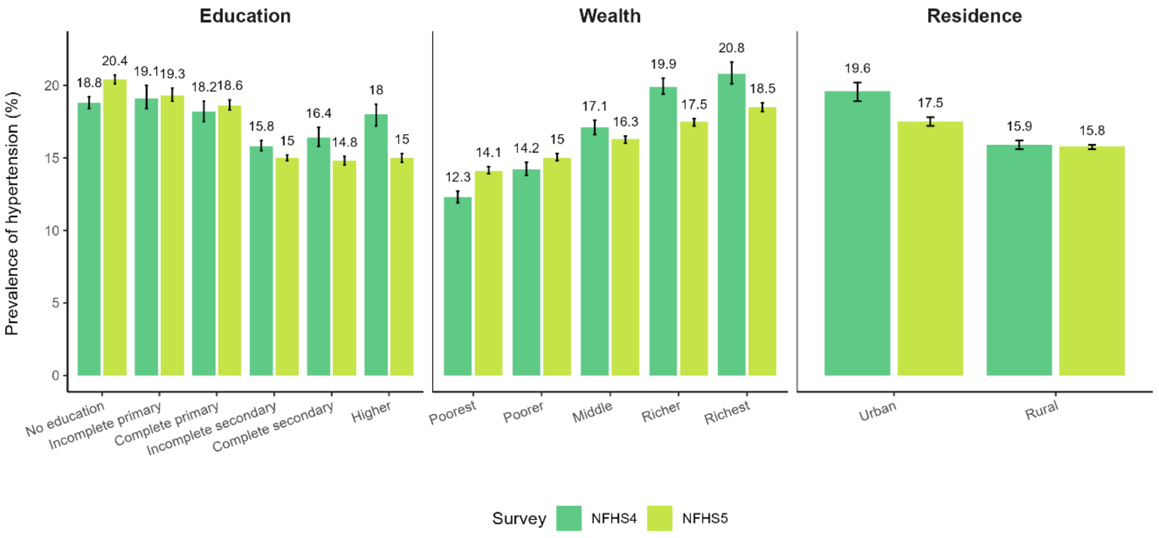

Figure S35. Prevalence of hypertension among adults aged 15-49 across subpopulations in each survey round

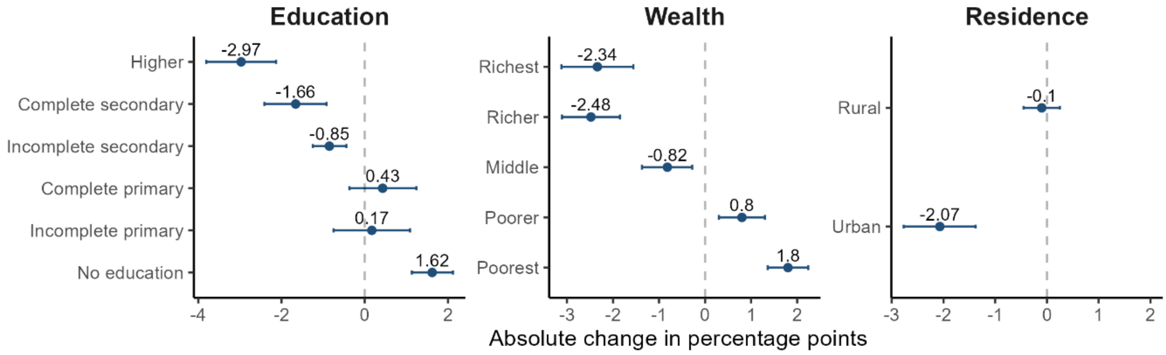

Figure S36. Absolute changes in the prevalence of hypertension among adults aged 15-49 across subpopulations

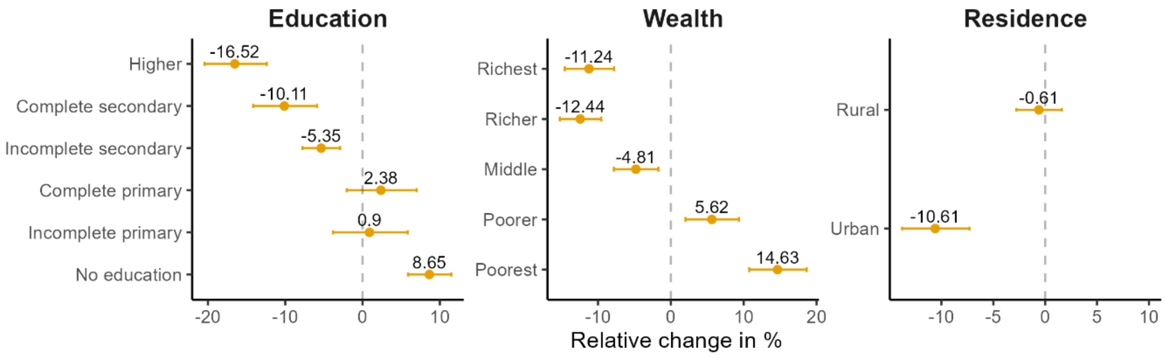

Figure S37. Relative changes in the prevalence of hypertension among adults aged 15-49 across subpopulations

High blood pressure

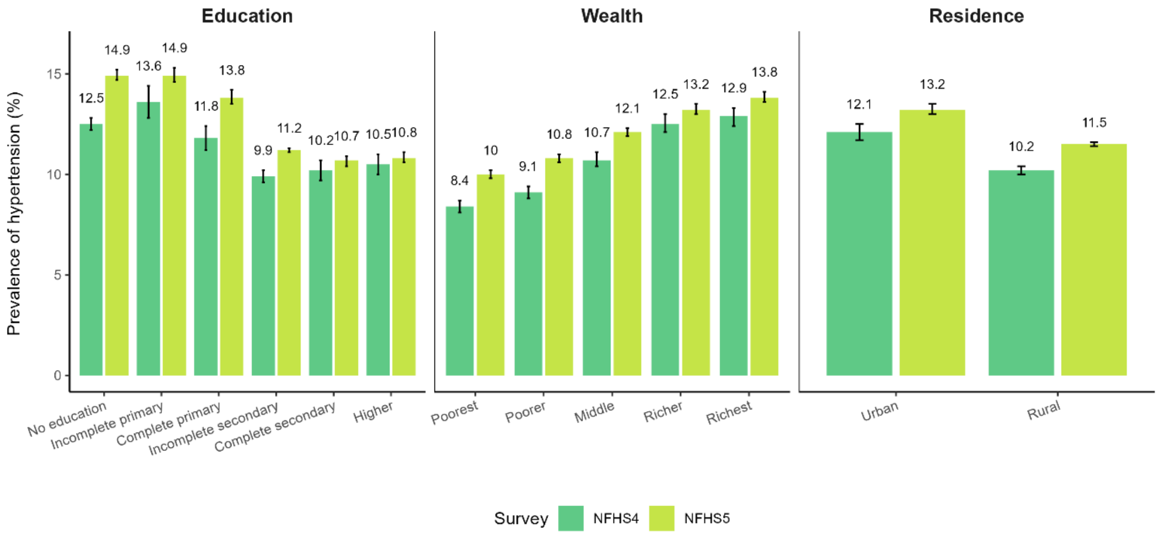

Figure S38. Prevalence of high blood pressure among adults aged 15-49 across subpopulations in each survey round

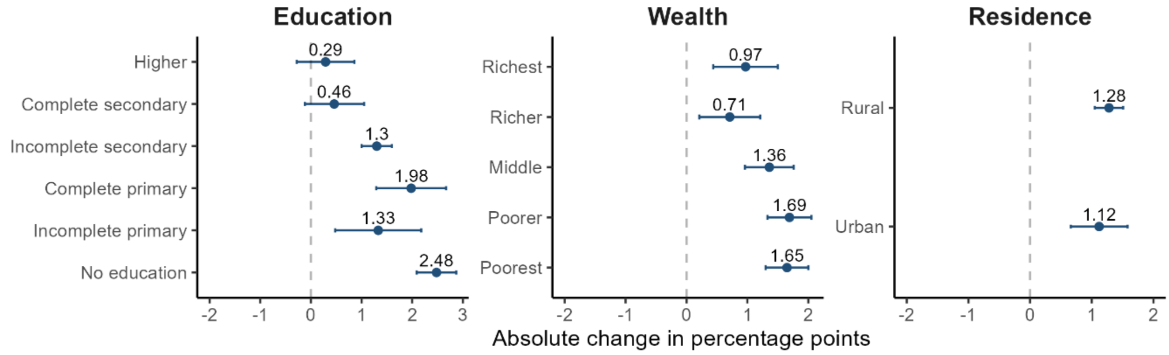

Figure S39. Absolute changes in the prevalence of high blood pressure among adults aged 15-49 across subpopulations

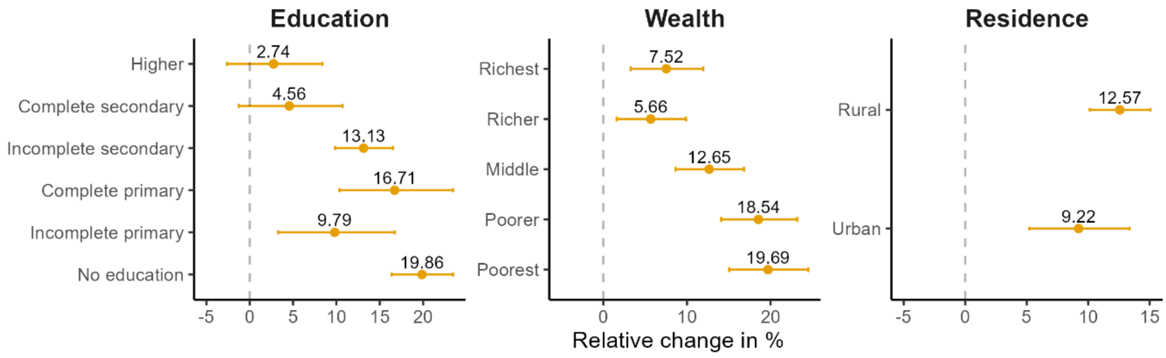

Figure S40. Relative changes in the prevalence of high blood pressure among adults aged 15-49 across subpopulations

Told to have high blood pressure

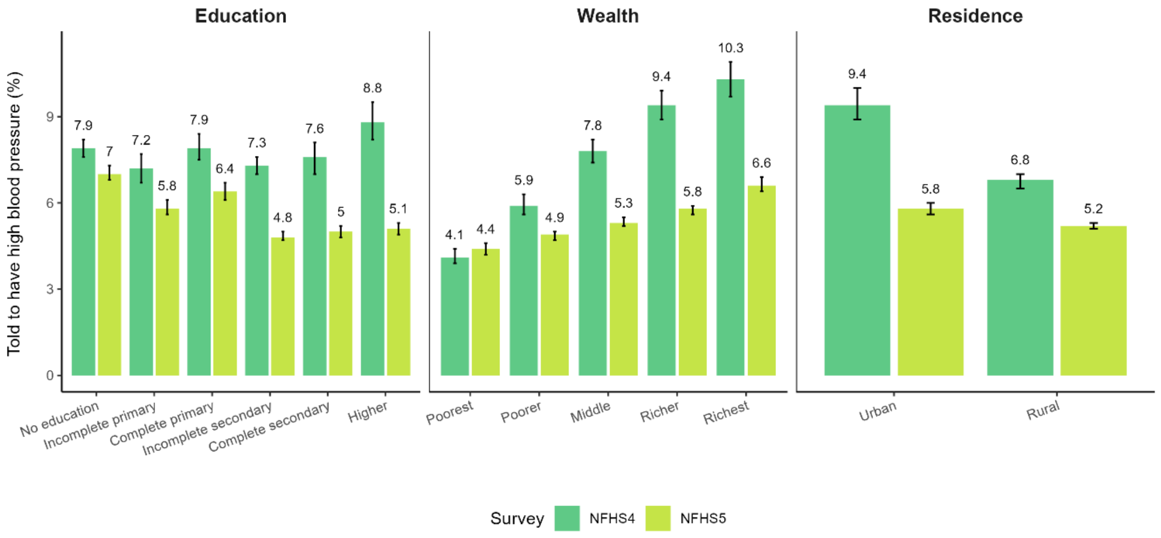

Figure S41. Prevalence of adults aged 15-49 diagnosed with hypertension across subpopulations in each survey round

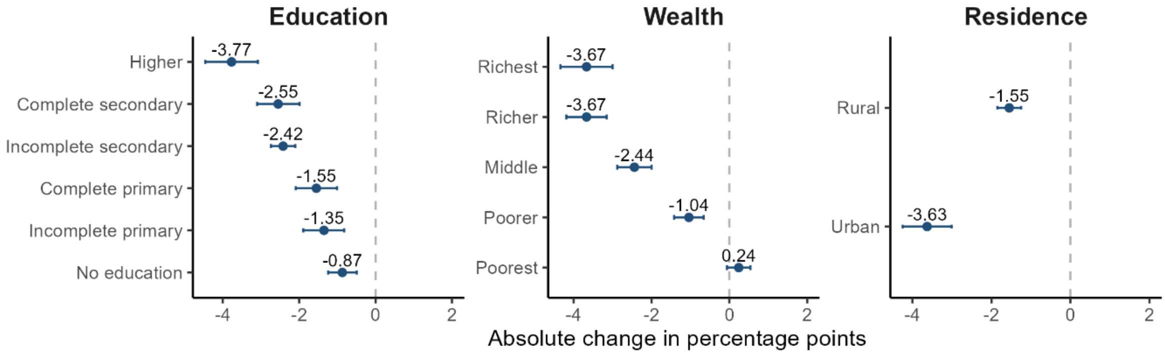

Figure S42. Absolute changes in the prevalence of adults aged 15-49 diagnosed with hypertension across subpopulations

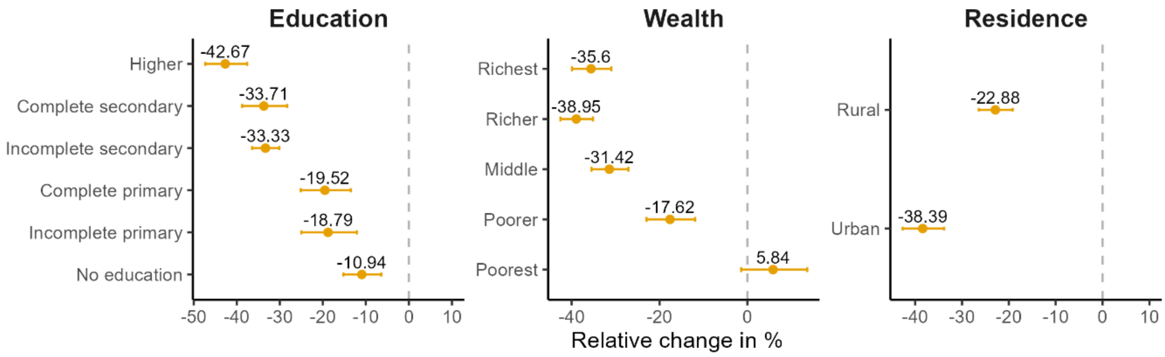

Figure S43. Relative changes in the prevalence of adults aged 15-49 diagnosed with hypertension across subpopulations

Age-standardized
Smoking

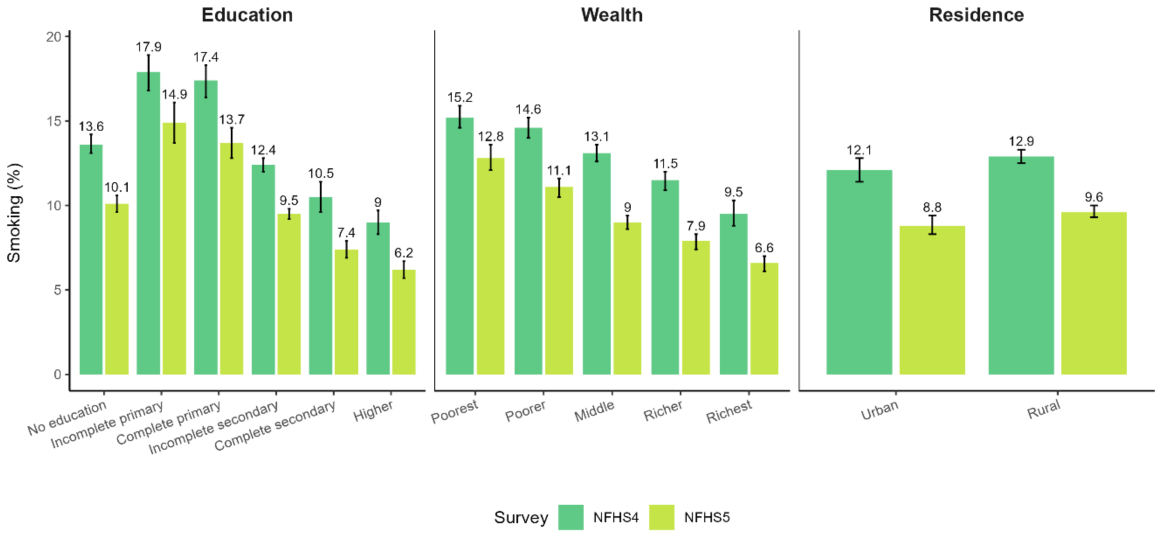

Figure S44. Age-standardized prevalence of tobacco consumption among adults aged 15-49 across subpopulations in each survey round

Cigarette smoking

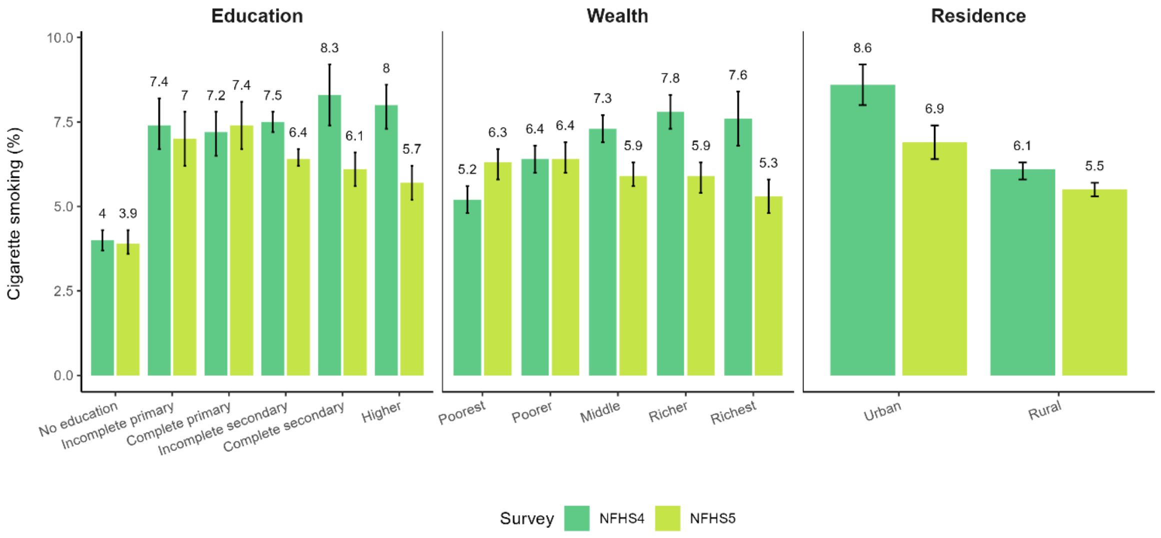

Figure S45. Age-standardized prevalence of cigarette smoking among adults aged 15-49 across subpopulations in each survey round

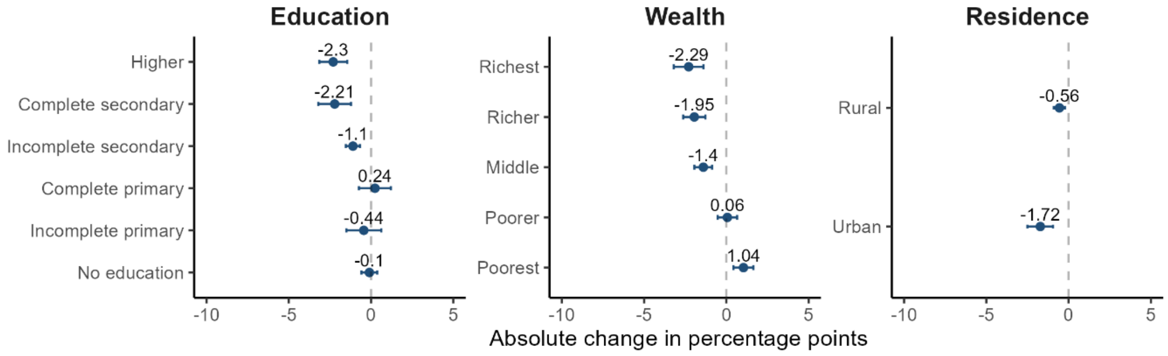

Figure S46. Age-standardized absolute changes in the prevalence of cigarette smoking among adults aged 15-49 across subpopulations

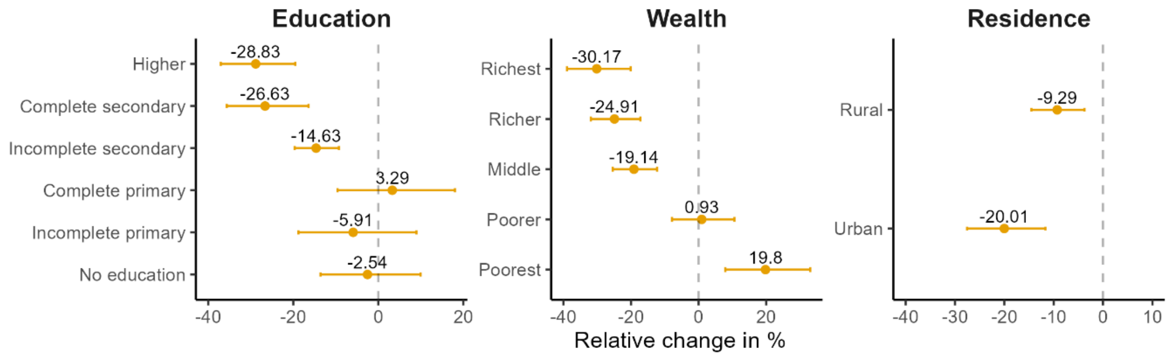

Figure S47. Age-standardized relative changes in the prevalence of cigarette smoking among adults aged 15-49 across subpopulations

Tobacco consumption

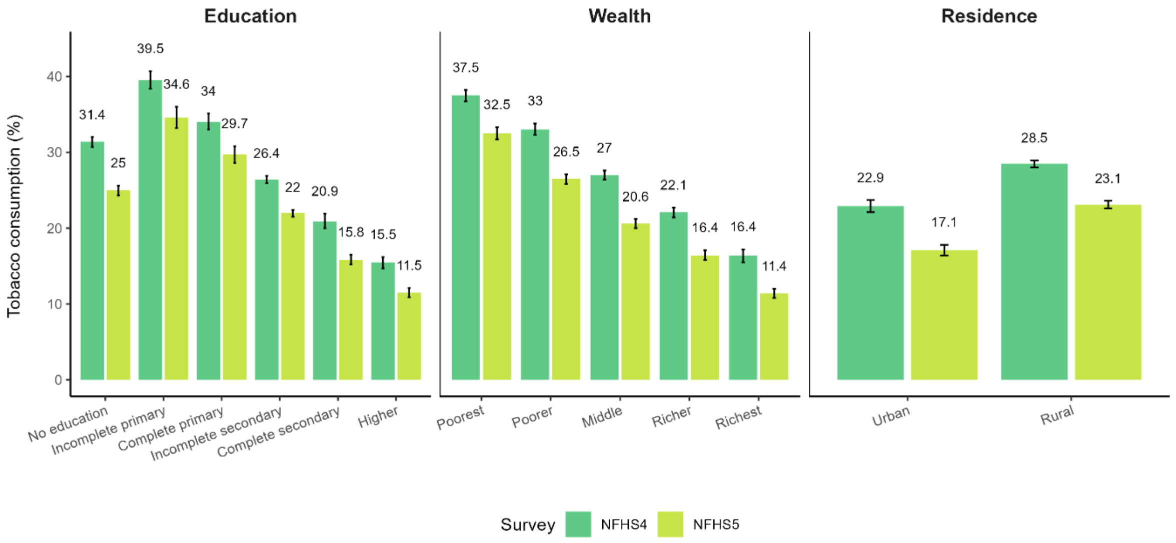

Figure S48. Age-adjusted prevalence of tobacco consumption among adults aged 15-49 across subpopulations in each survey round

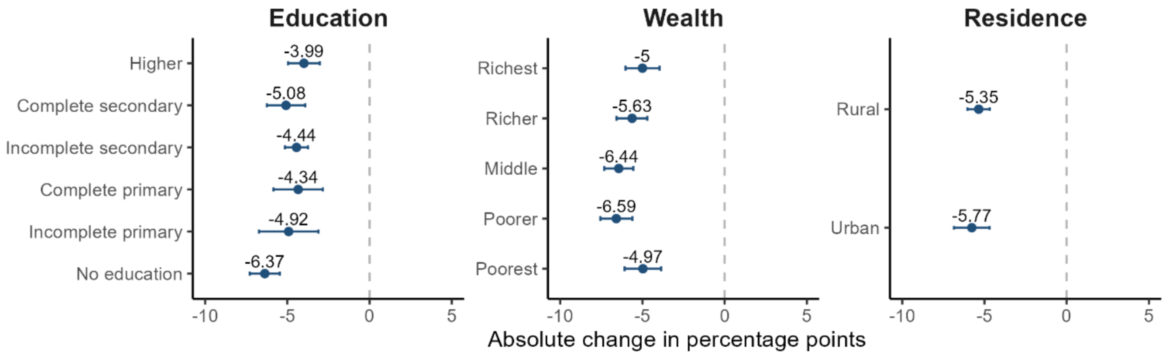

Figure S49. Age-standardized absolute changes in the prevalence of tobacco consumption among adults aged 15-49 across subpopulations

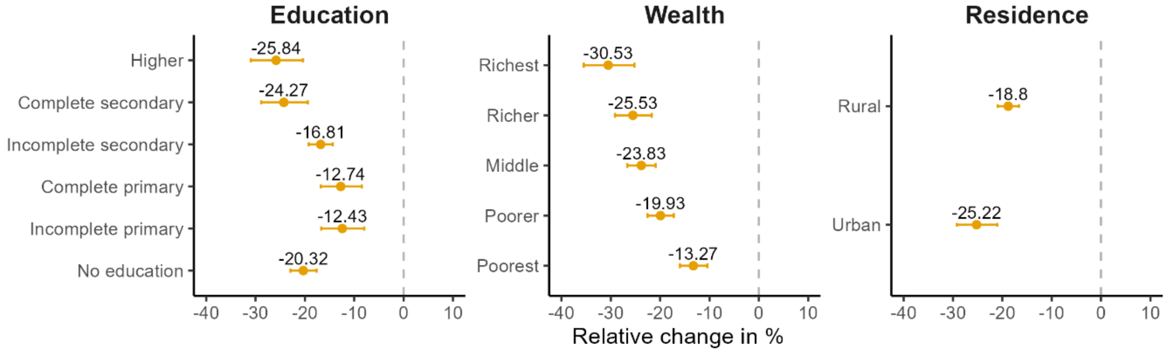

Figure S50. Age-standardized relative changes in the prevalence of tobacco consumption among adults aged 15-49 across subpopulations

Overweight

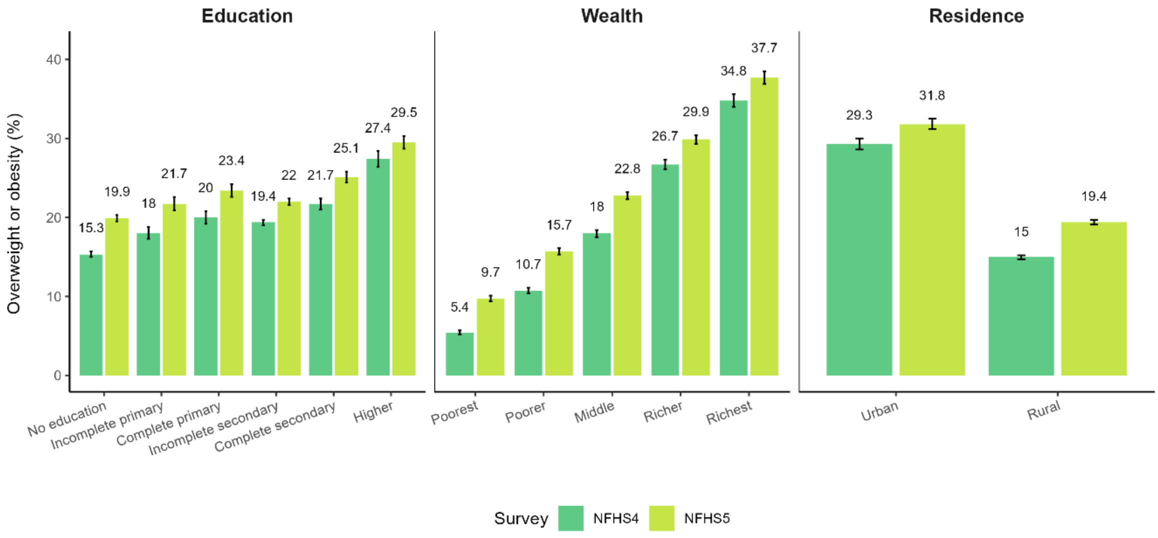

Figure S51. Age-standardized prevalence of overweight or obesity among adults aged 15-49 across subpopulations in each survey round

Obesity

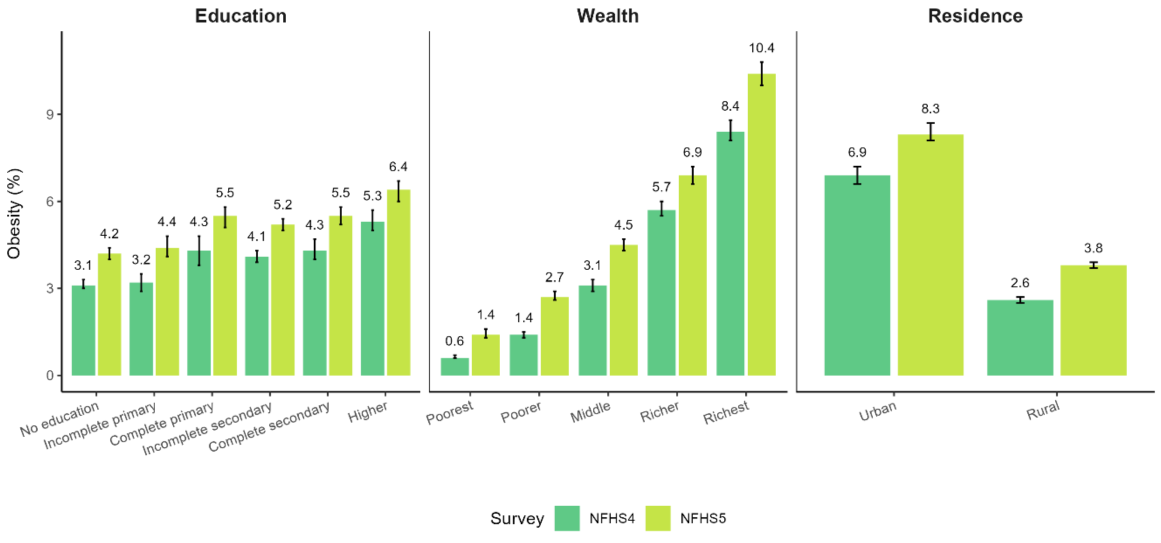

Figure S52. Age-standardized prevalence of obesity among adults aged 15-49 across subpopulations in each survey round

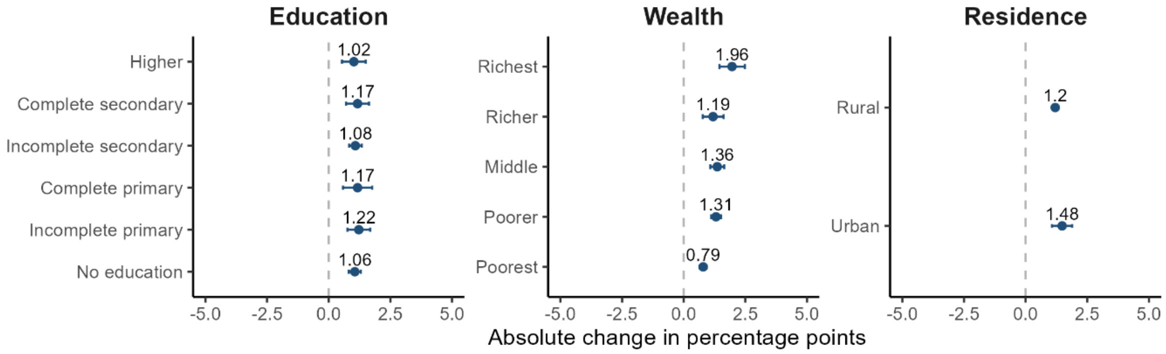

Figure S53. Age-standardized absolute changes in the prevalence of obesity among adults aged 15-49 across subpopulations

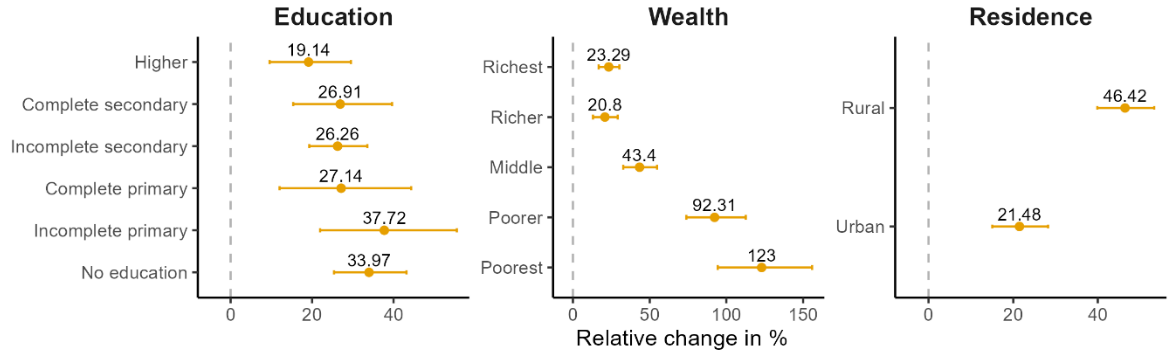

Figure S54. Age-standardized relative changes in the prevalence of obesity among adults aged 15-49 across subpopulations

BMI ≥ 23 kg/m^2

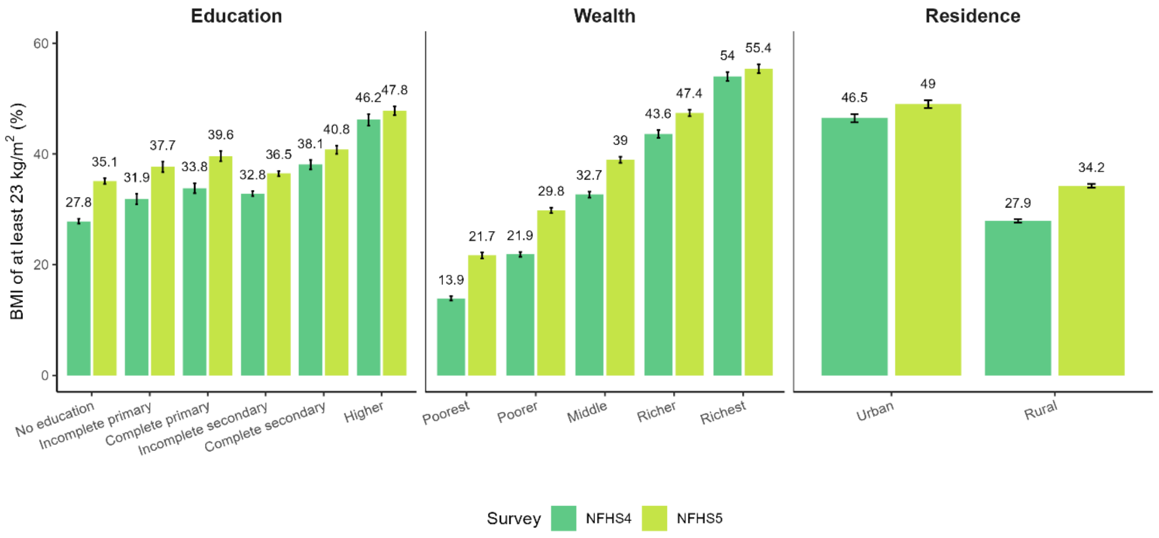

Figure S55. Age-standardized prevalence of having a BMI ≥ 23 kg/m^2 among adults aged 15-49 across subpopulations in each survey round

The outcome variable is a binary indicator for having unhealthy weight with increased CVD risk based on the ethnicity-specific threshold of 23 kg/m^2 as proposed by the WHO expert consultation on appropriate BMI cutoff points for Asian people in 2002.

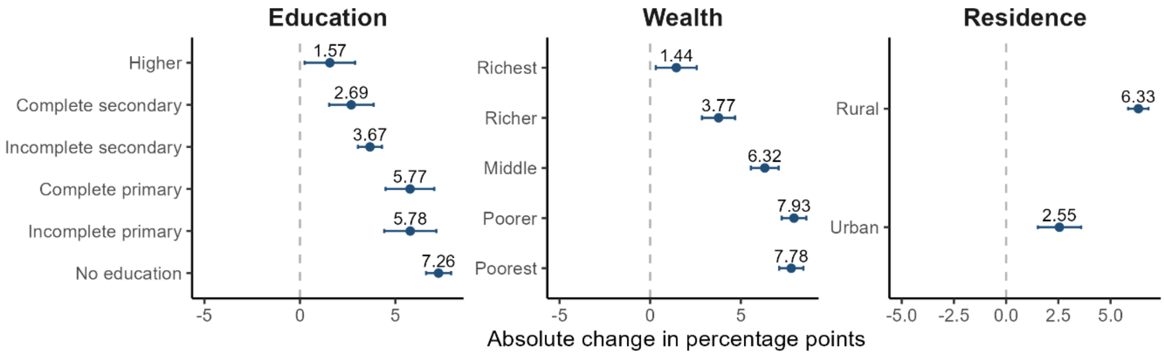

Figure S56. Age-standardized absolute changes in the prevalence of having a BMI ≥ 23 kg/m^2 among adults aged 15-49 across subpopulations

The outcome variable is a binary indicator for having unhealthy weight with increased CVD risk based on the ethnicity-specific threshold of 23 kg/m2 as proposed by the WHO expert consultation on appropriate BMI cutoff points for Asian people in 2002.

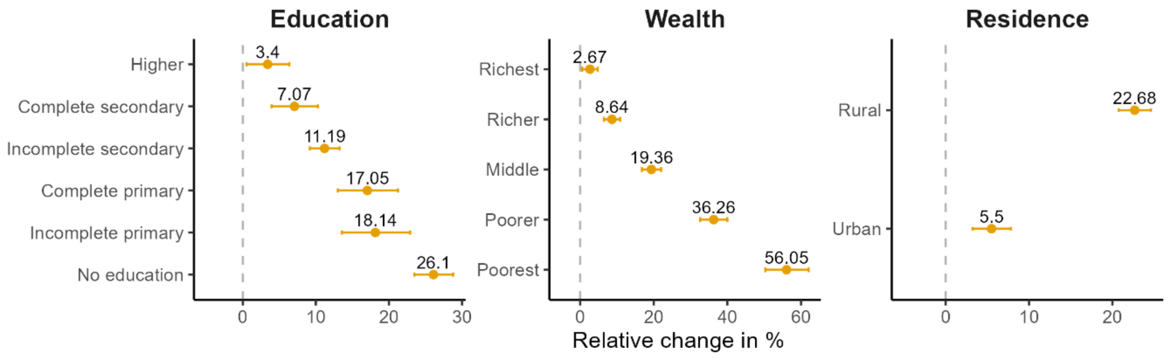

Figure S57. Age-standardized relative changes in the prevalence of having a BMI ≥ 23 kg/m^2 among adults aged 15-49 across subpopulations

The outcome variable is a binary indicator for having unhealthy weight with increased CVD risk based on the ethnicity-specific threshold of 23 kg/m^2 as proposed by the WHO expert consultation on appropriate BMI cutoff points for Asian people in 2002.

BMI ≥ 27.5 kg/m^2

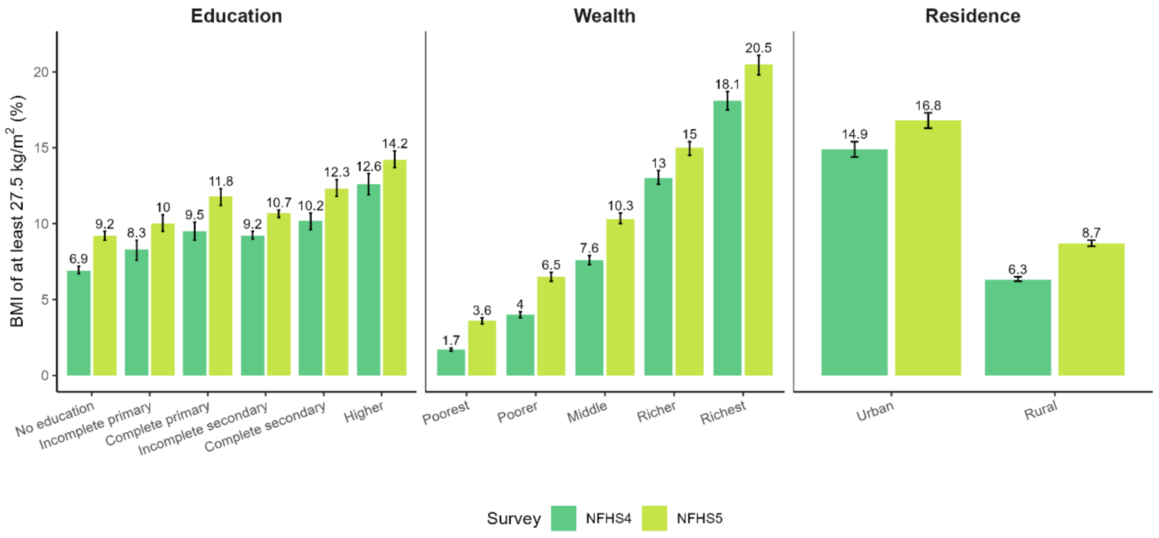

Figure S58. Age-standardized prevalence of having a BMI ≥ 27.5 kg/m^2 among adults aged 15-49 across subpopulations in each survey round

The outcome variable is a binary indicator for having unhealthy weight with high CVD risk based on the ethnicity-specific threshold of 27.5 kg/m^2 as proposed by the WHO expert consultation on appropriate BMI cutoff points for Asian people in 2002.

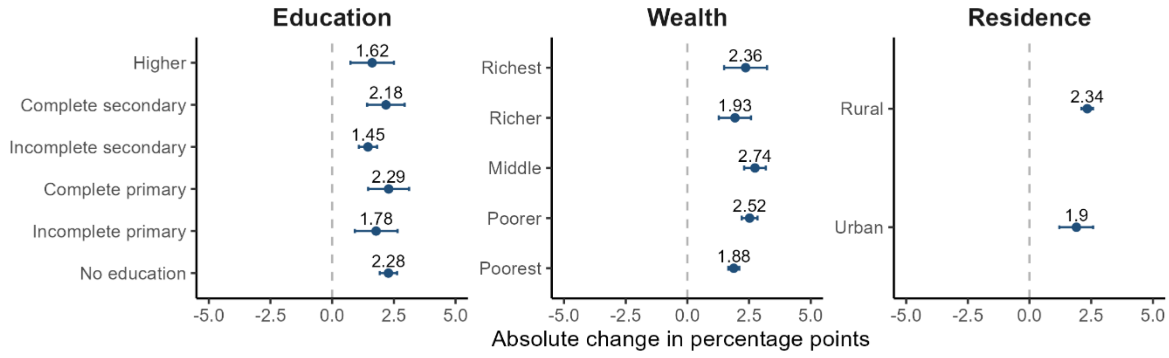

Figure S59. Age-standardized absolute changes in the prevalence of having a BMI ≥ 27.5 kg/m^2 among adults aged 15-49 across subpopulations

The outcome variable is a binary indicator for having unhealthy weight with high CVD risk based on the ethnicity-specific threshold of 27.5 kg/m^2 as proposed by the WHO expert consultation on appropriate BMI cutoff points for Asian people in 2002.

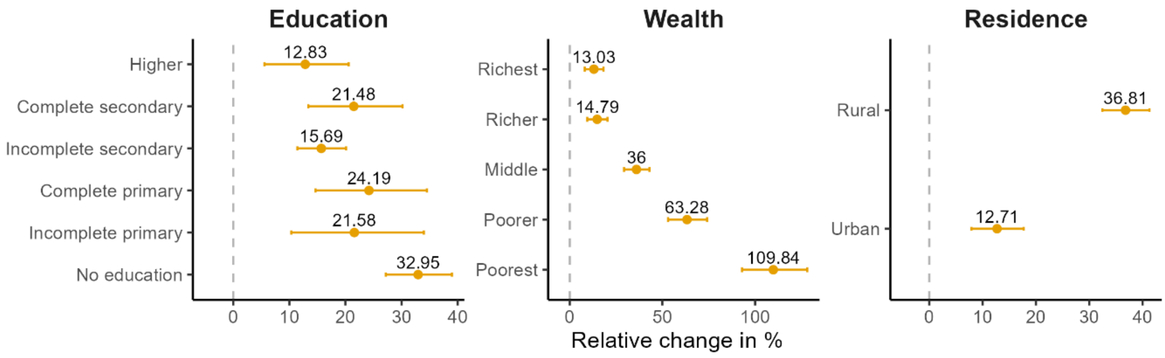

Figure S60. Age-standardized relative changes in the prevalence of having a BMI ≥ 27.5 kg/m^2 among adults aged 15-49 across subpopulations

The outcome variable is a binary indicator for having unhealthy weight with high CVD risk based on the ethnicity-specific threshold of 27.5 kg/m^2 as proposed by the WHO expert consultation on appropriate BMI cutoff points for Asian people in 2002.

Diabetes

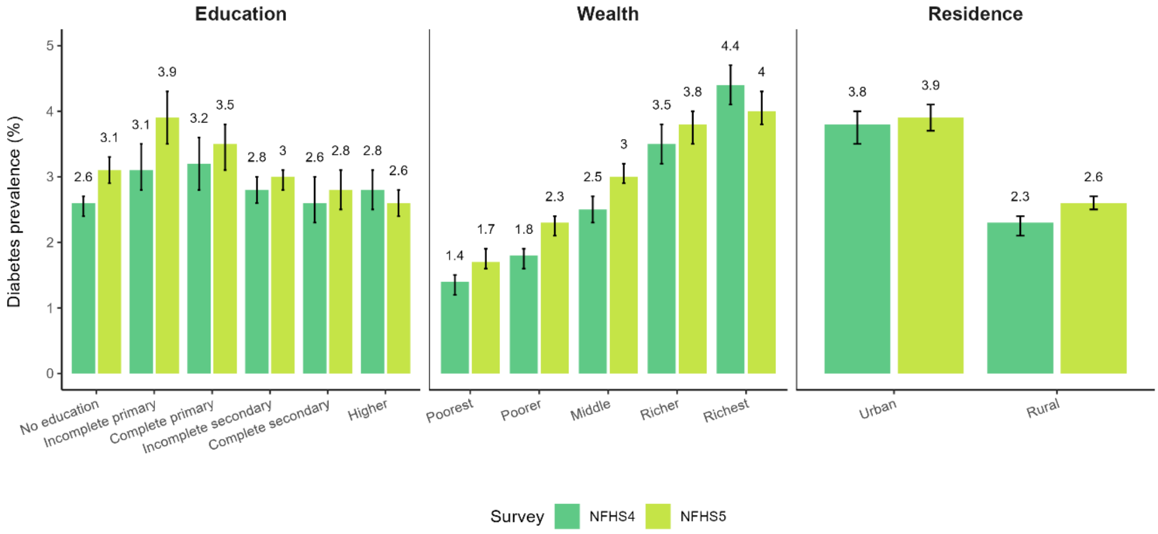

Figure S61. Age-standardized prevalence of diabetes among adults aged 15-49 across subpopulations in each survey round

Diabetes (alternative definition)

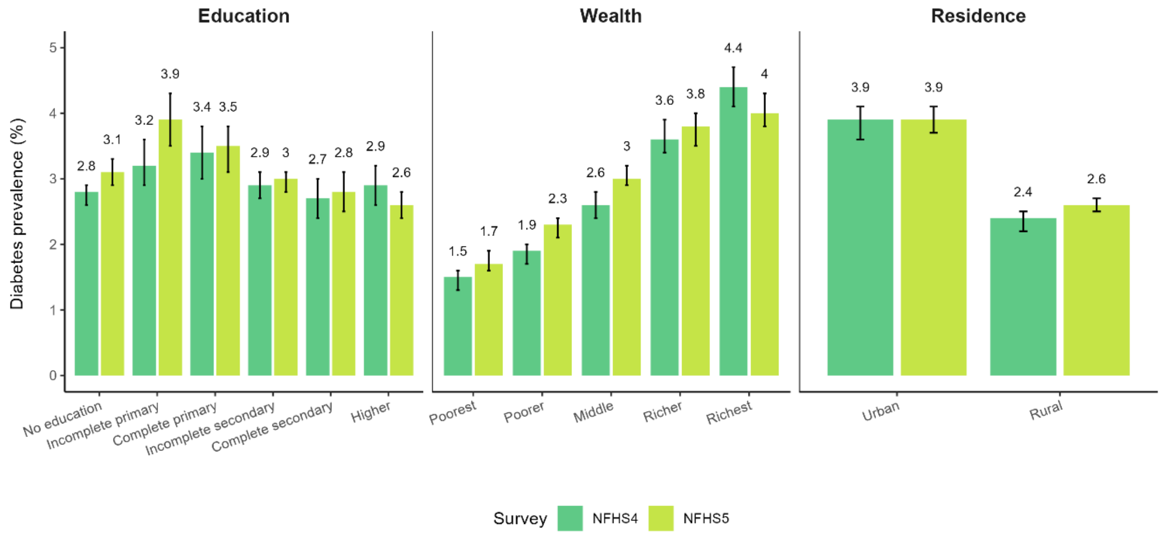

Figure S62. Age-standardized prevalence of diabetes (based on an alternative definition that takes into account fasting status) among adults aged 15-49 across subpopulations in each survey round

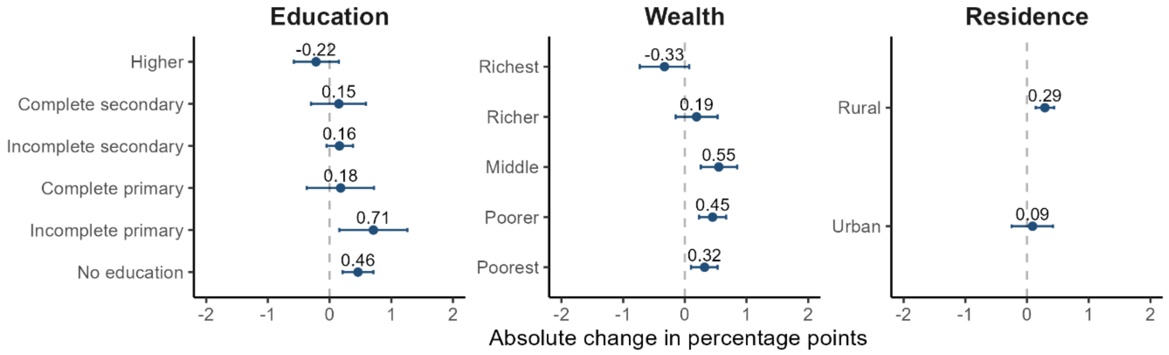

Figure S63. Age-standardized absolute changes in the prevalence of diabetes (based on an alternative definition that takes into account fasting status) among adults aged 15-49 across subpopulations

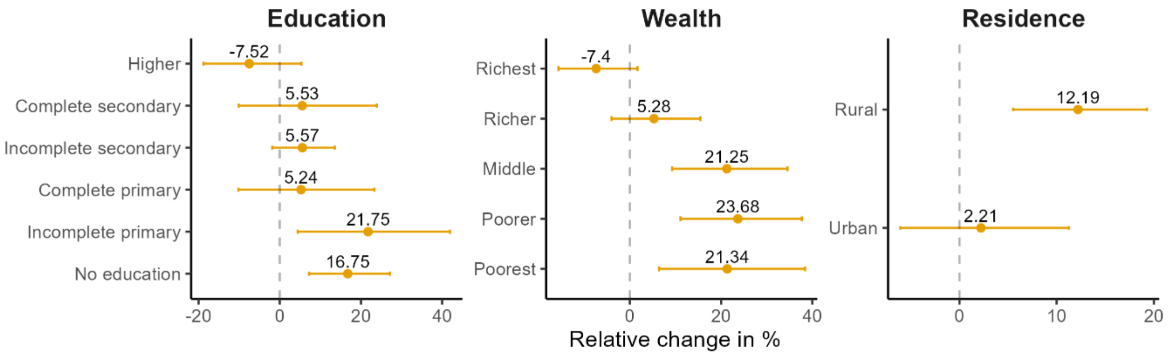

Figure S64. Age-standardized relative changes in the prevalence of diabetes (based on an alternative definition that takes into account fasting status) among adults aged 15-49 across subpopulations

High blood glucose

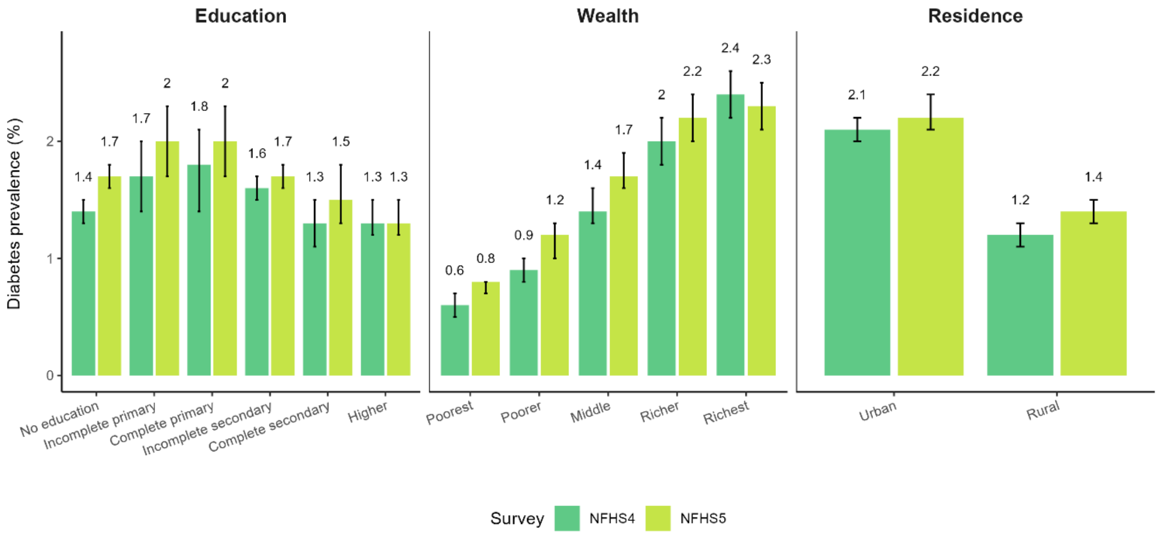

Figure S65. Age-standardized prevalence of high blood glucose among adults aged 15-49 across subpopulations in each survey round

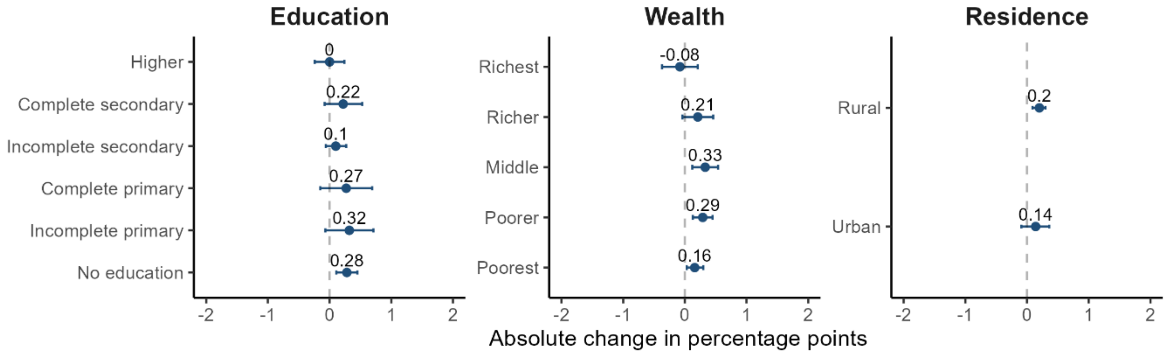

Figure S66. Age-standardized absolute changes in the prevalence of high blood glucose among adults aged 15-49 across subpopulations

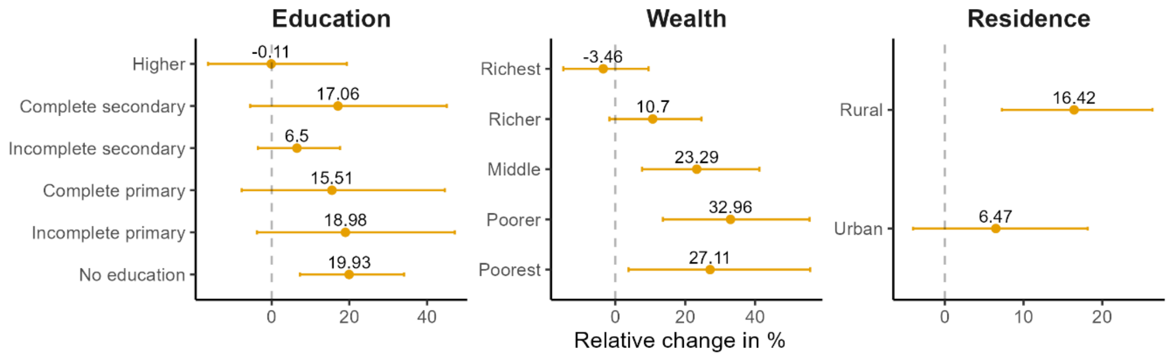

Figure S67. Age-standardized relative changes in the prevalence of high blood glucose among adults aged 15-49 across subpopulations

Self-reported diabetes

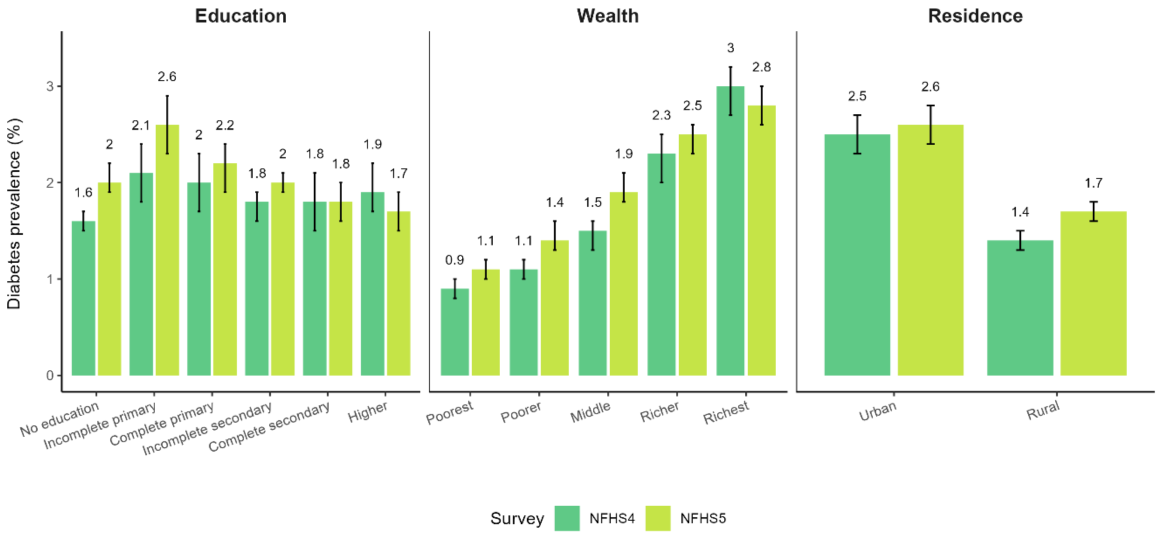

Figure S68. Age-standardized prevalence of self-reported diabetes among adults aged 15-49 across subpopulations in each survey round

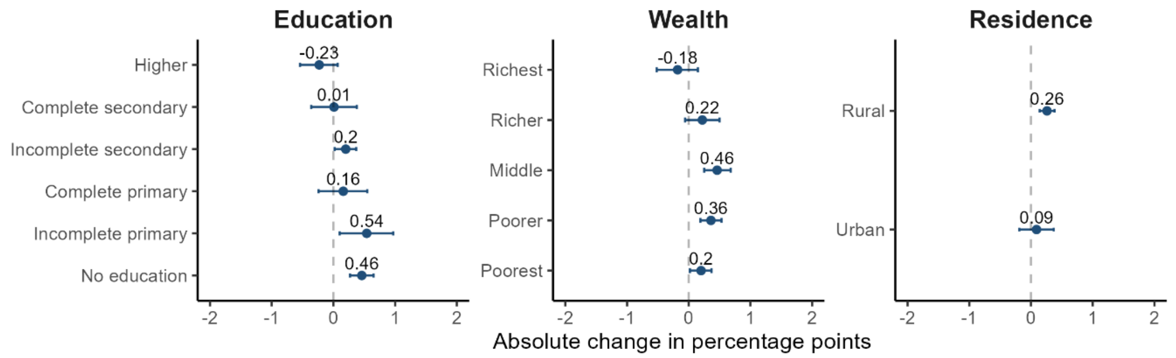

Figure S69. Age-standardized absolute changes in the prevalence of self-reported diabetes among adults aged 15-49 across subpopulations

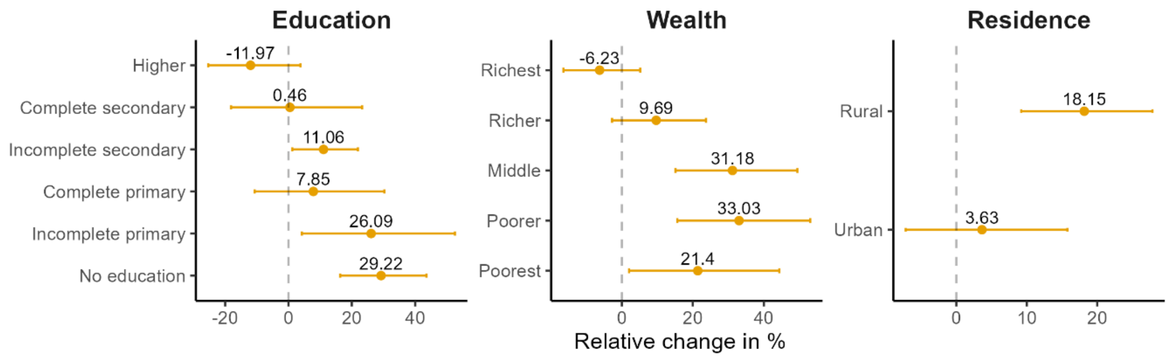

Figure S70. Age-standardized relative changes in the prevalence of self-reported diabetes among adults aged 15-49 across subpopulations

Hypertension

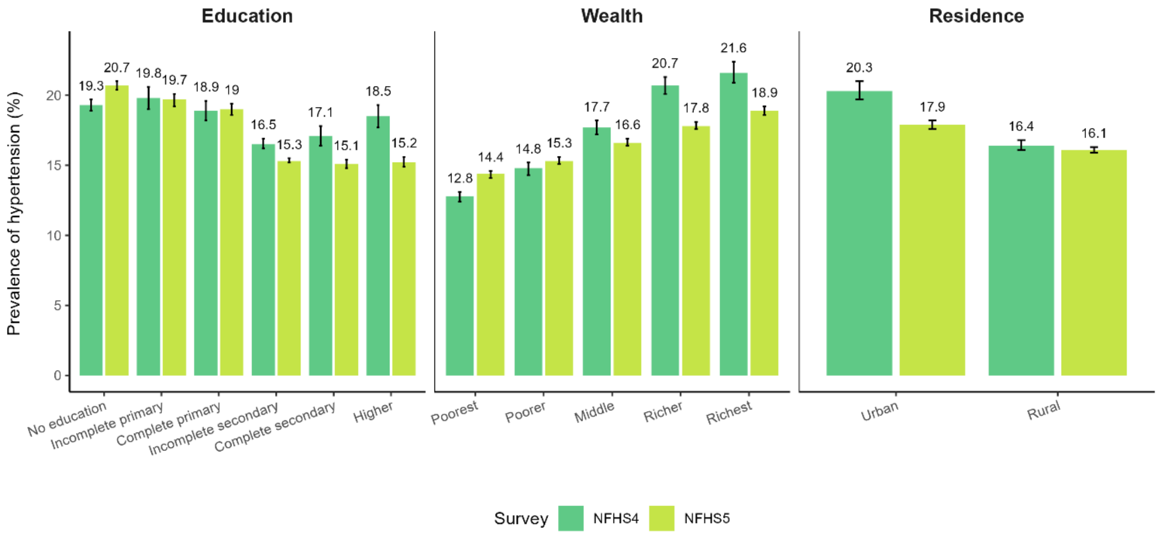

Figure S71. Age-standardized prevalence of hypertension among adults aged 15-49 across subpopulations in each survey round

High blood pressure

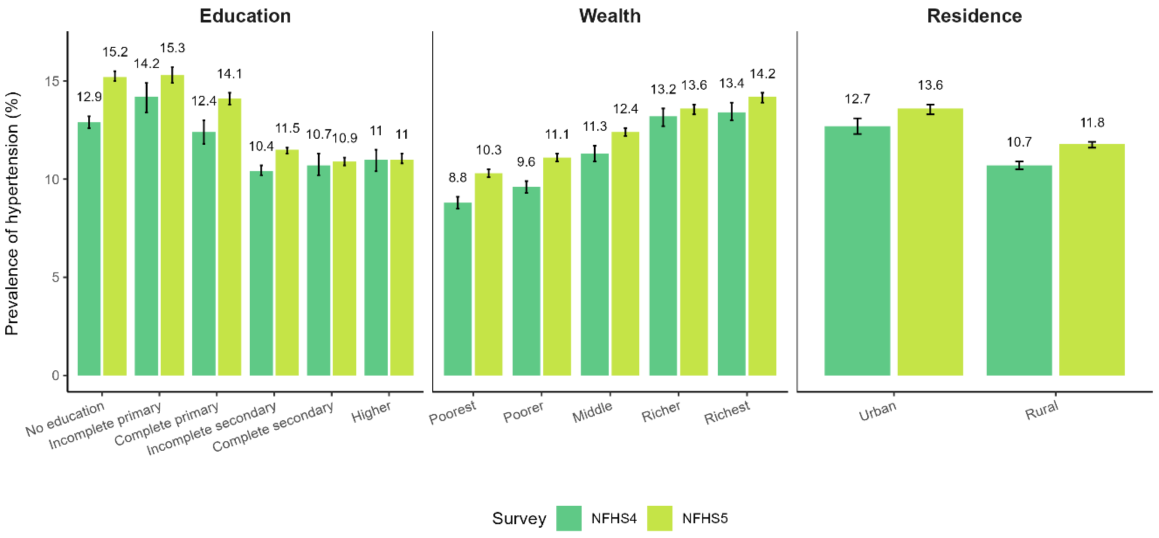

Figure S72. Age-standardized prevalence of high blood pressure among adults aged 15-49 across subpopulations in each survey round

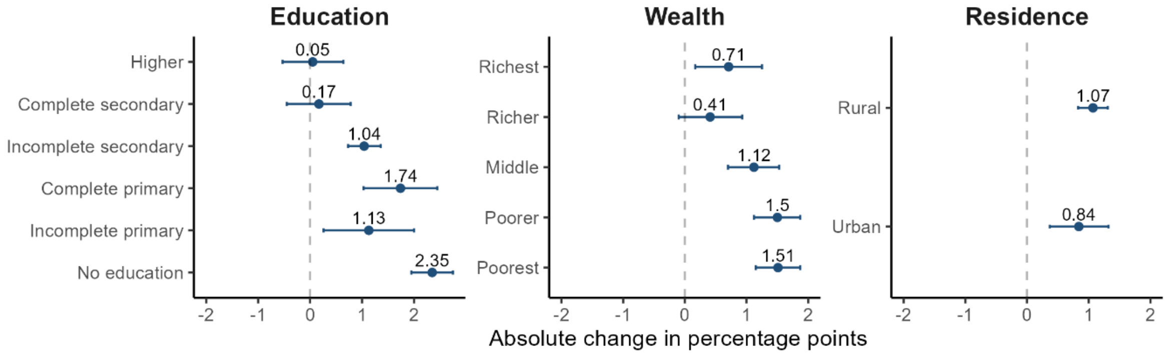

Figure S73. Age-standardized absolute changes in the prevalence of high blood pressure among adults aged 15-49 across subpopulations

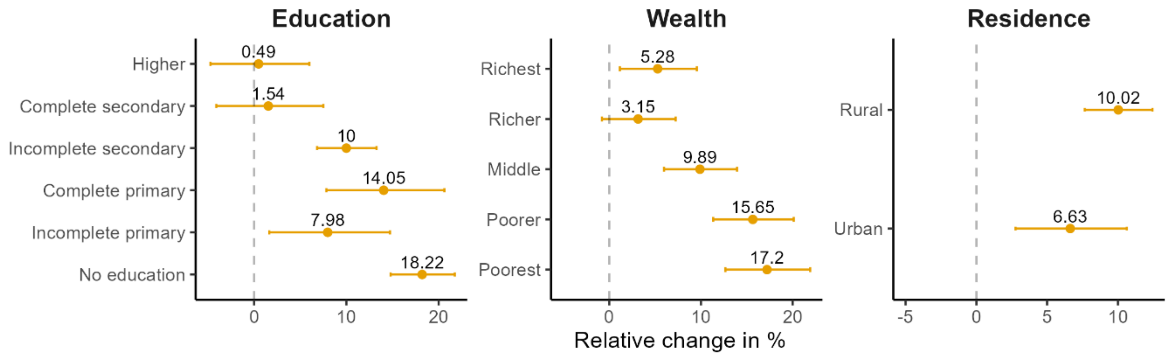

Figure S74. Age-standardized relative changes in the prevalence of high blood pressure among adults aged 15-49 across subpopulations

Told to have high blood pressure

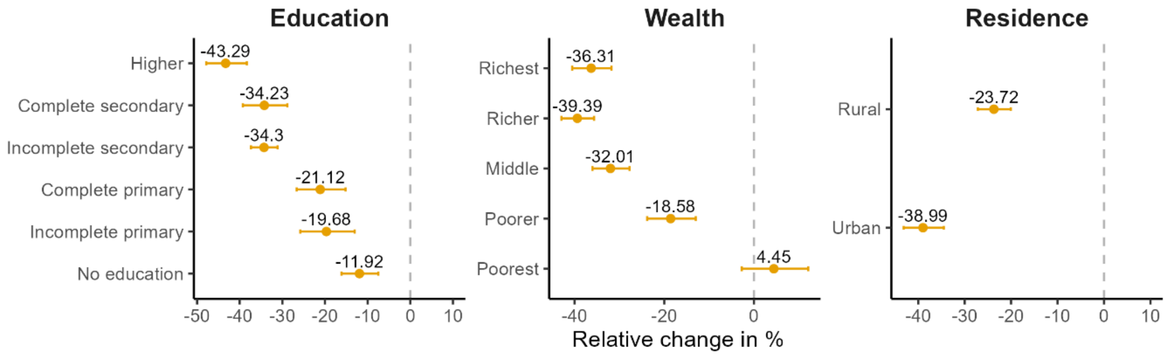

Figure S75. Age-standardized prevalence of adults aged 15-49 diagnosed with hypertension across subpopulations in each survey round

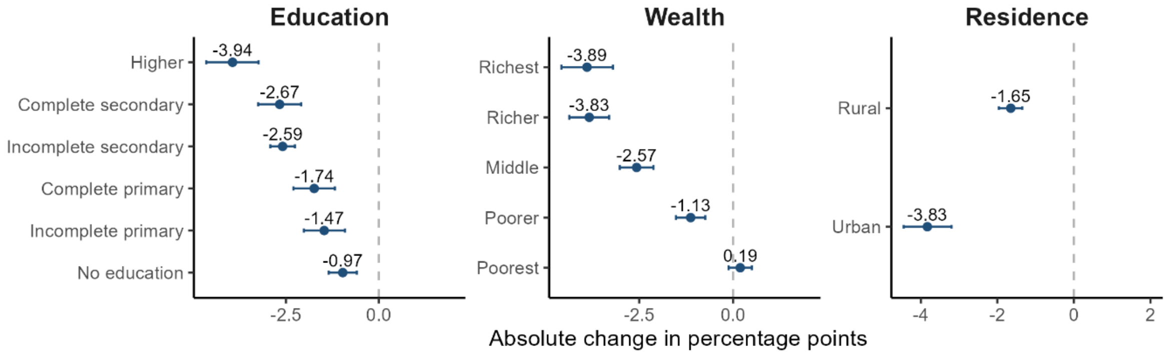

Figure S76. Age-standardized absolute changes in the prevalence of adults aged 15-49 diagnosed with hypertension across subpopulations

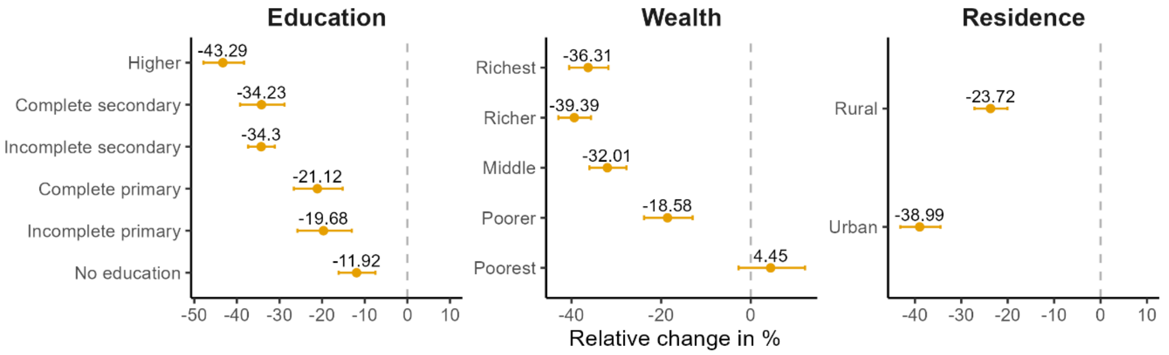

Figure S77. Age-standardized relative changes in the prevalence of adults aged 15-49 diagnosed with hypertension across subpopulations

Results stratified by region

NFHS-4

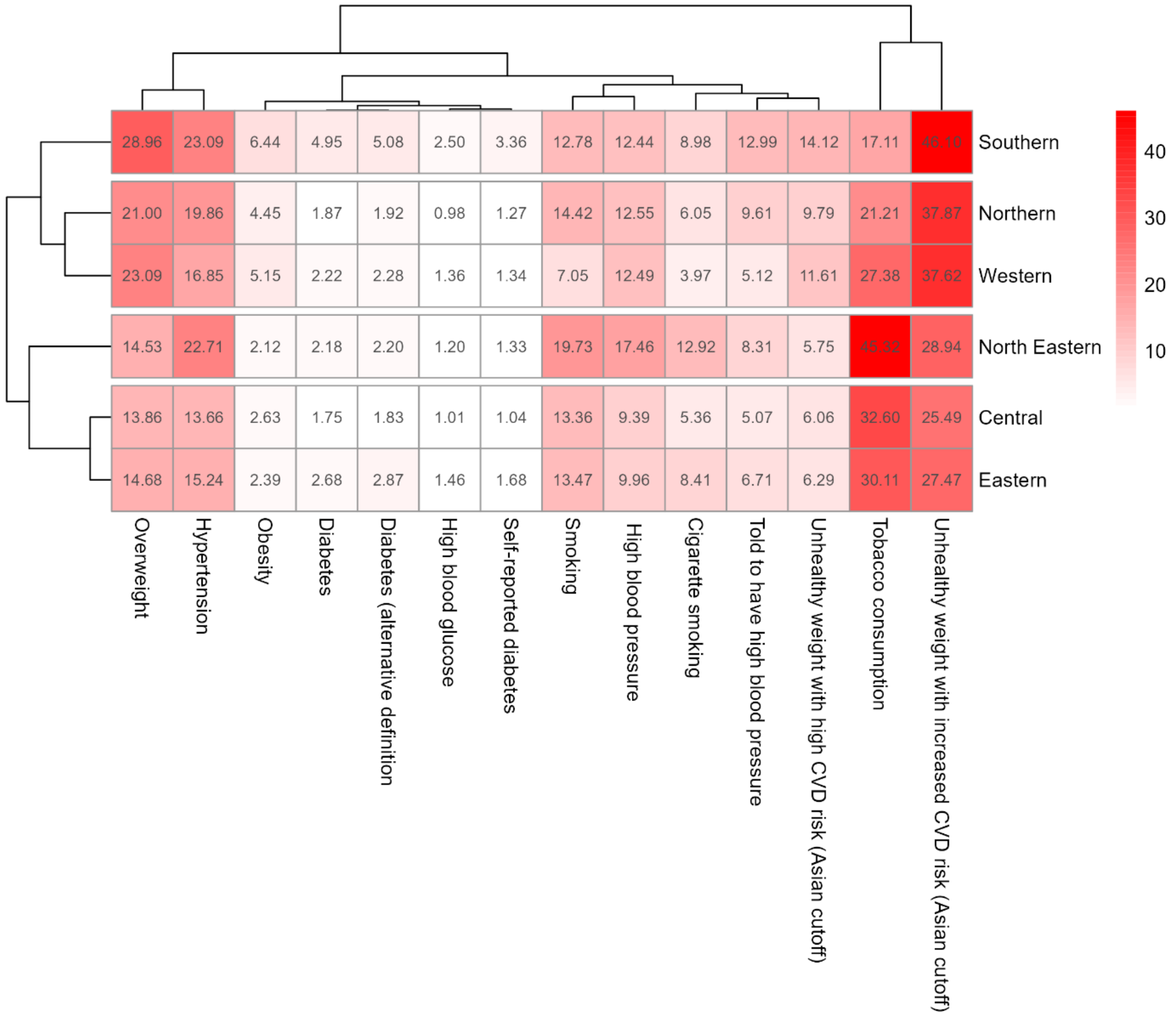

Figure S78. Age-standardized prevalence for each CVD risk factor across regions at the time of the NFHS-4

The heatmap is based on hierarchical cluster analysis with average linkage and Euclidean distance.

Absolute changes

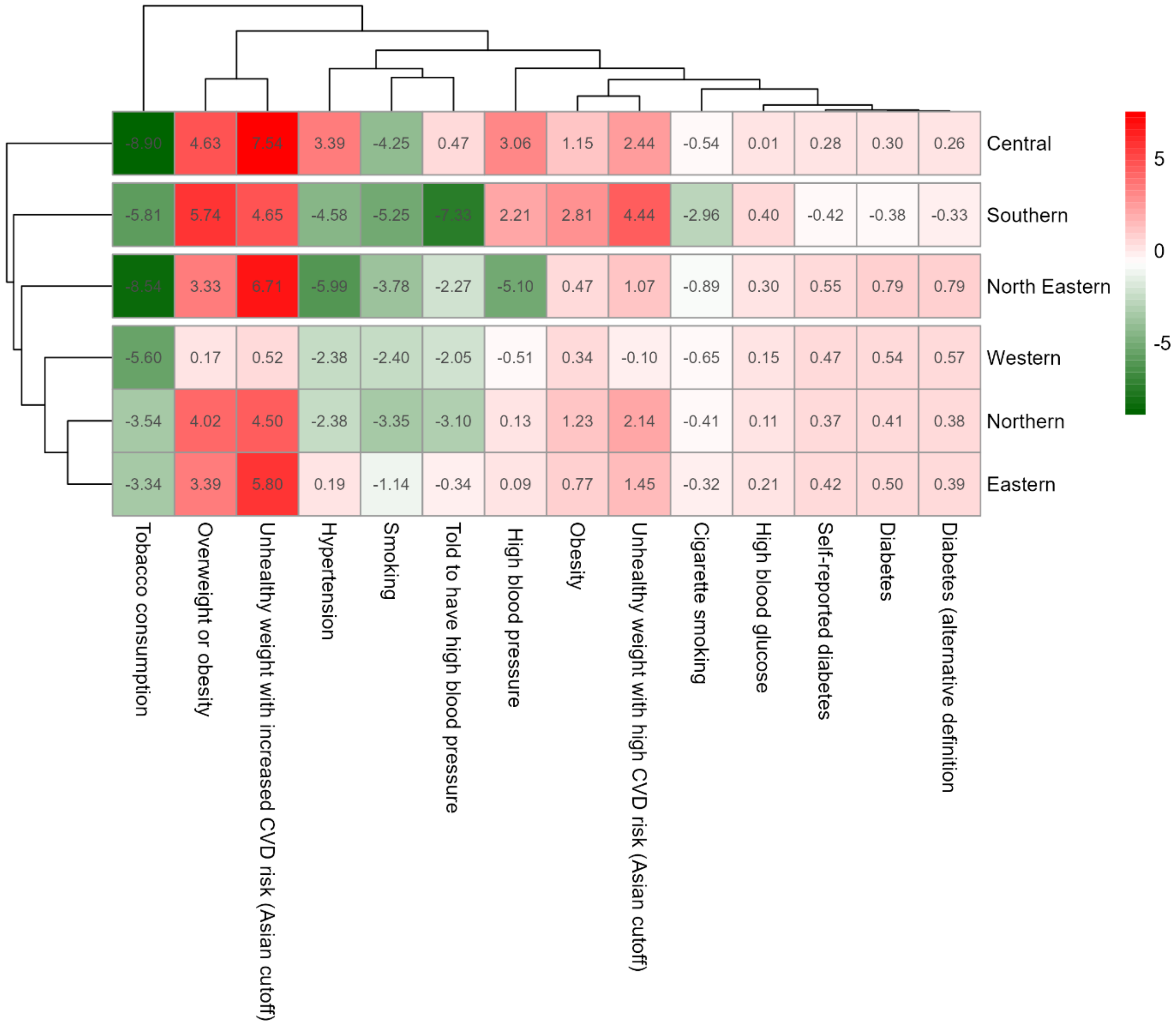

Figure S79. Age-standardized absolute change in the prevalence of each CVD risk factor across regions between the NFHS-4 and the NFHS-5
The heatmap is based on hierarchical cluster analysis with average linkage and Euclidean distance.

Relative changes

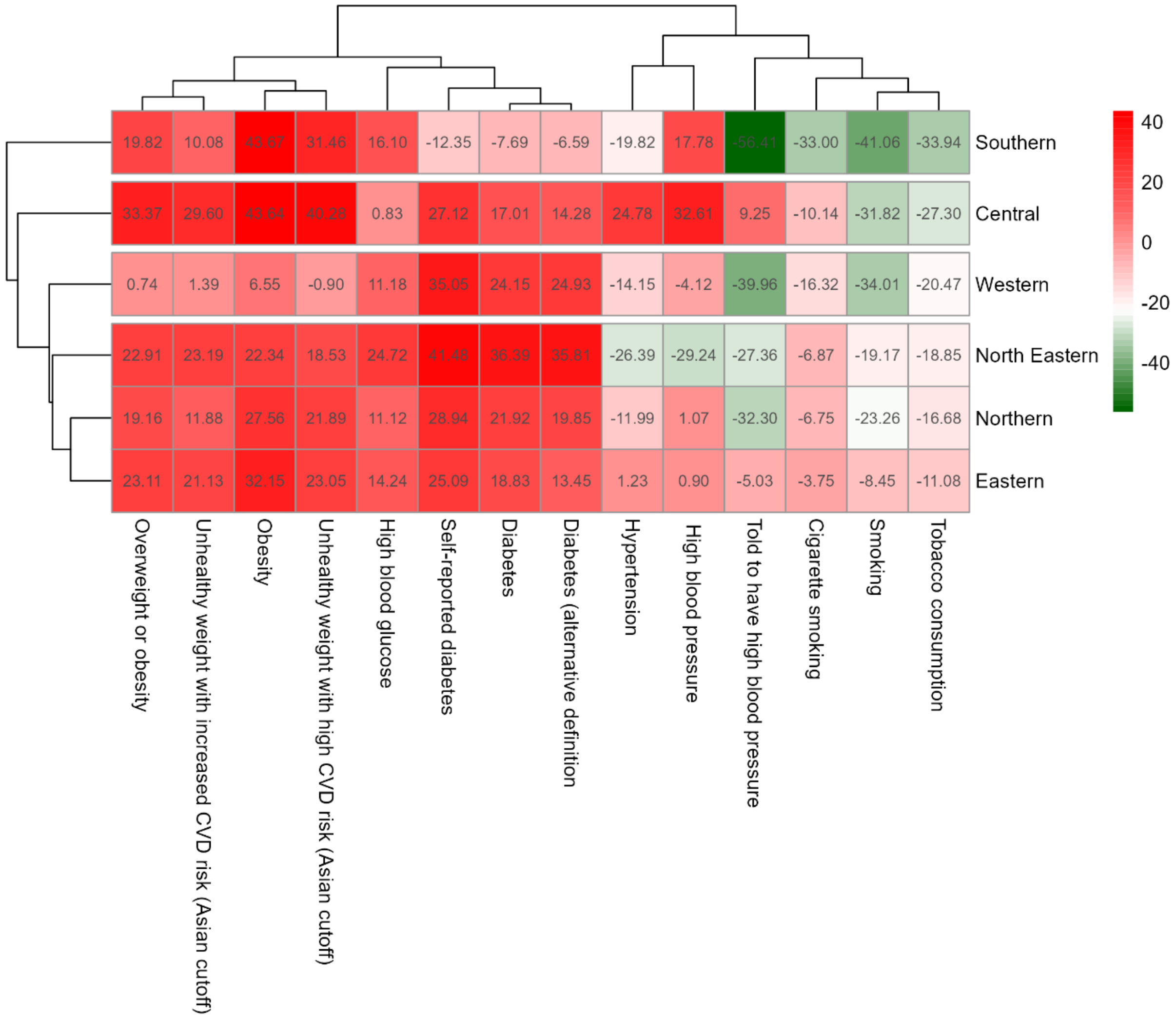

Figure S80. Age-standardized relative change in the prevalence of each CVD risk factor across regions between the NFHS-4 and the NFHS-5

The heatmap is based on hierarchical cluster analysis with average linkage and Euclidean distance.

Results stratified by regional level of development

NFHS-4

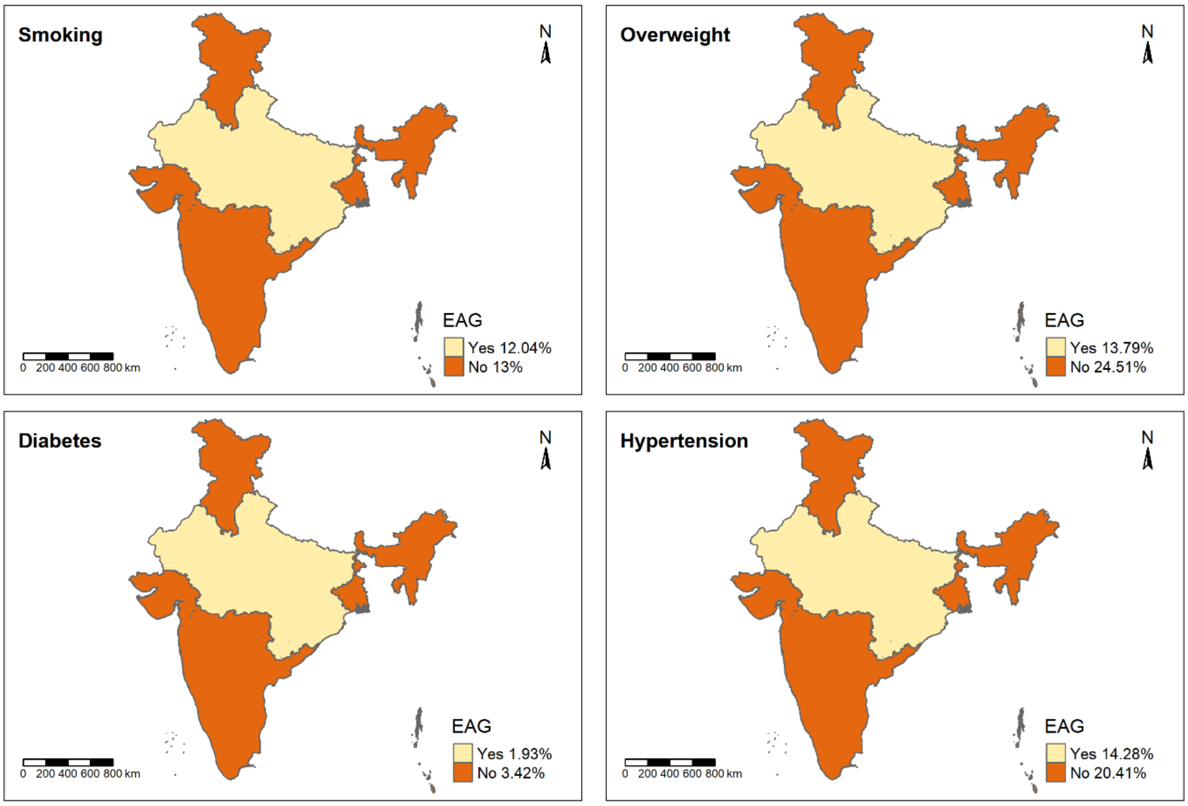

Figure S81. Age-standardized prevalence of CVD risk factors in the Empowered Action Group (EAG) states compared to other states and union territories at the time of the NFHS-4.

Absolute changes

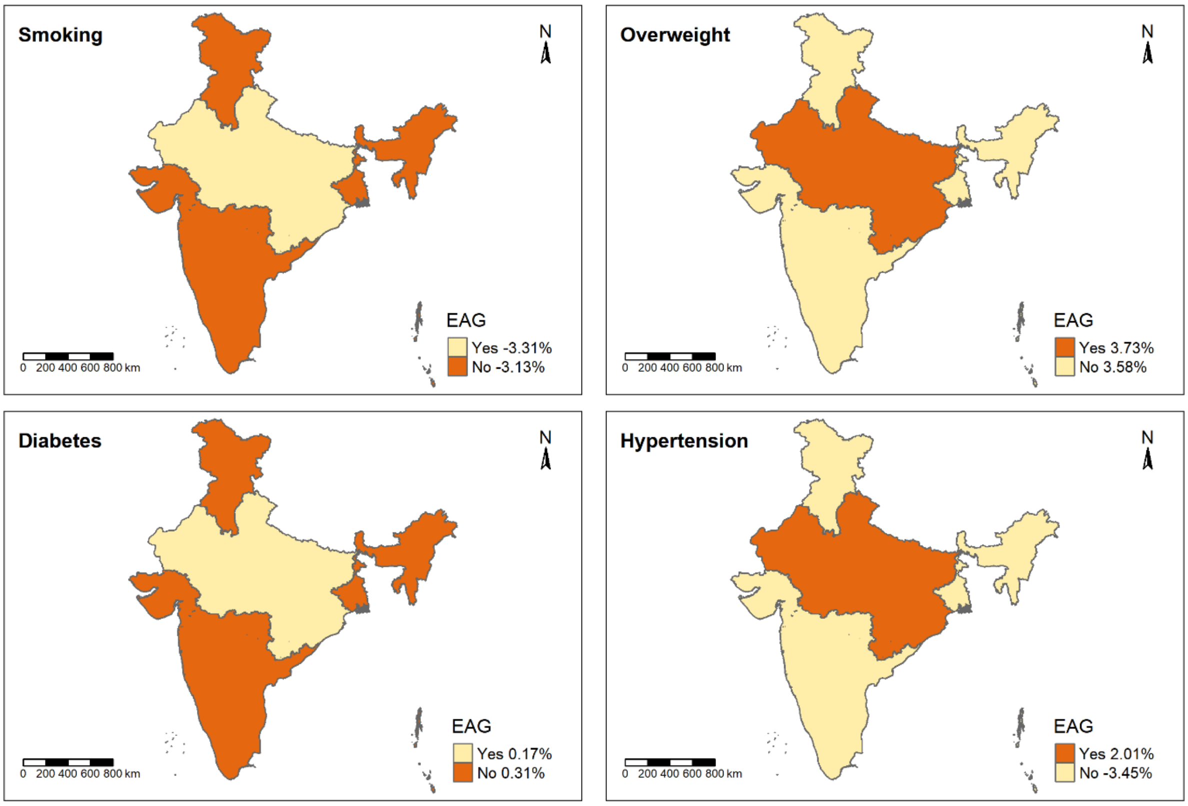

Figure S82. Age-standardized absolute changes (percentage points) in the prevalence of CVD risk factors in the Empowered Action Group (EAG) states compared to other states and union territories

Relative changes

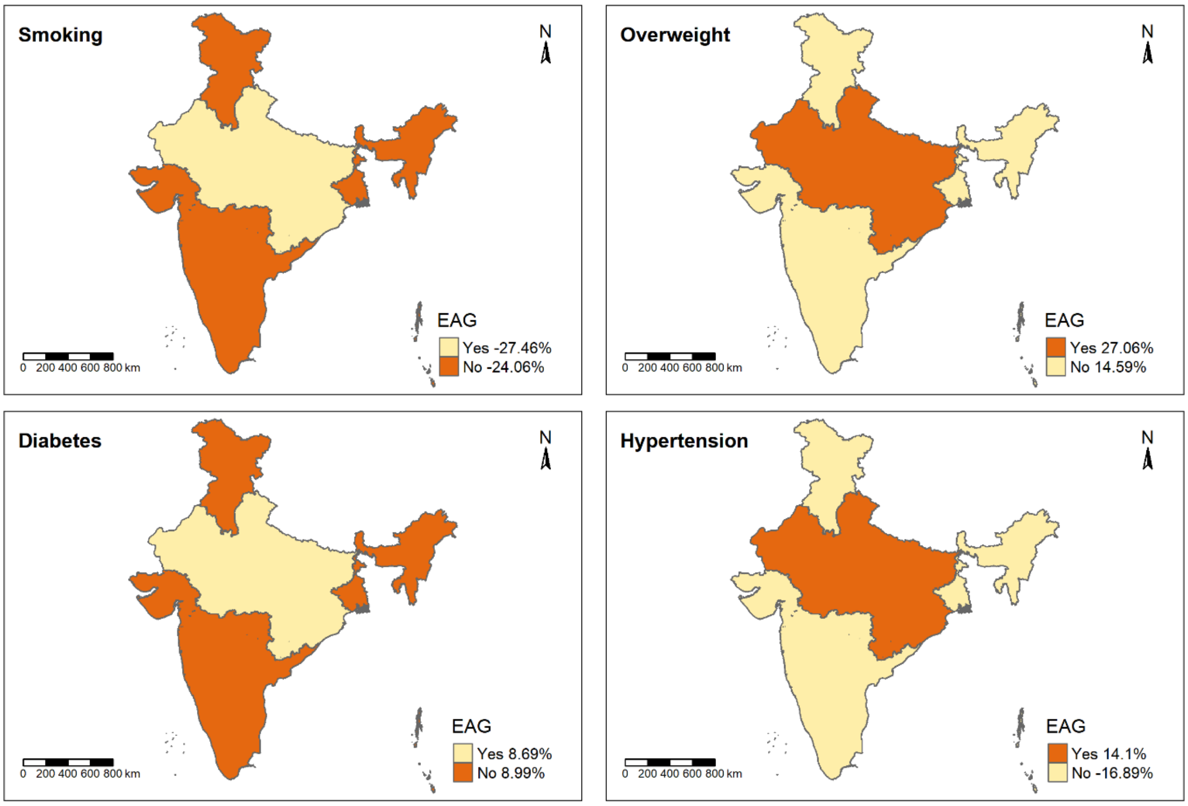

Figure S83. Age-standardized relative changes (%) in the prevalence of CVD risk factors in the Empowered Action Group (EAG) states compared to other states and union territories

Results stratified by sex and socioeconomic status or place of residence

Women

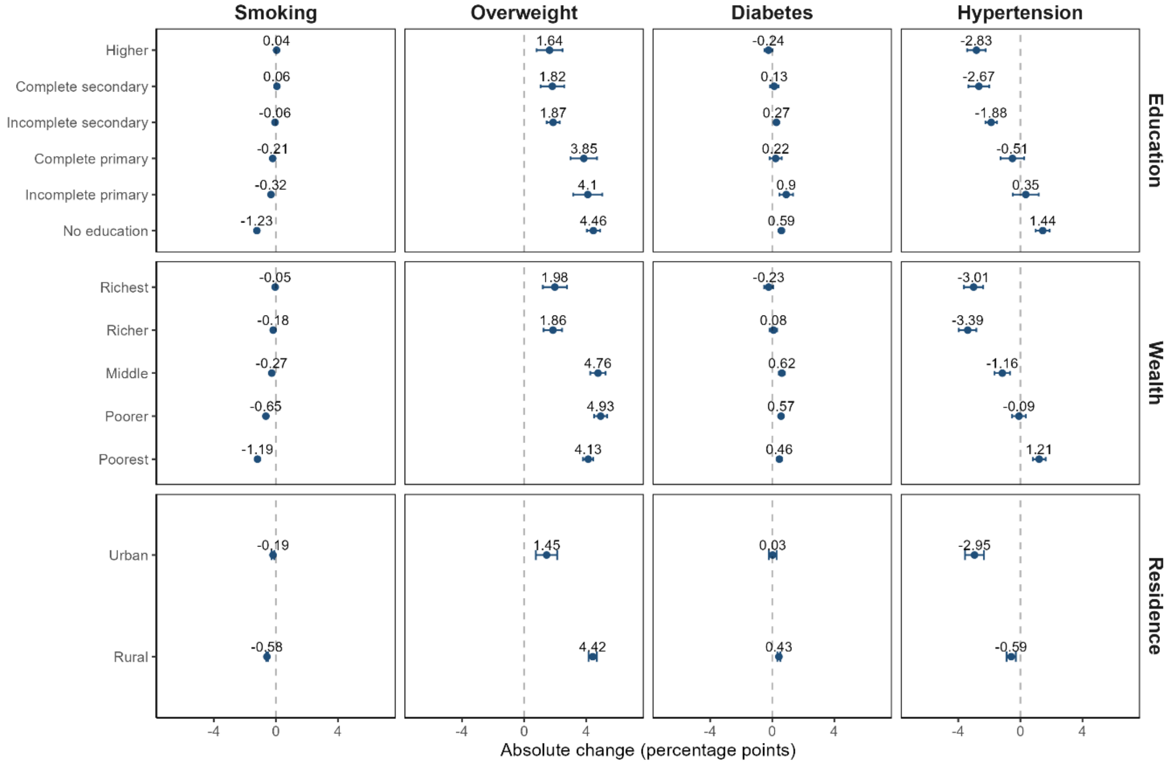

Figure S84. Age-standardized absolute change in each CVD risk factor among women aged 15-49 by wealth quintile, level of education, and place of residence.

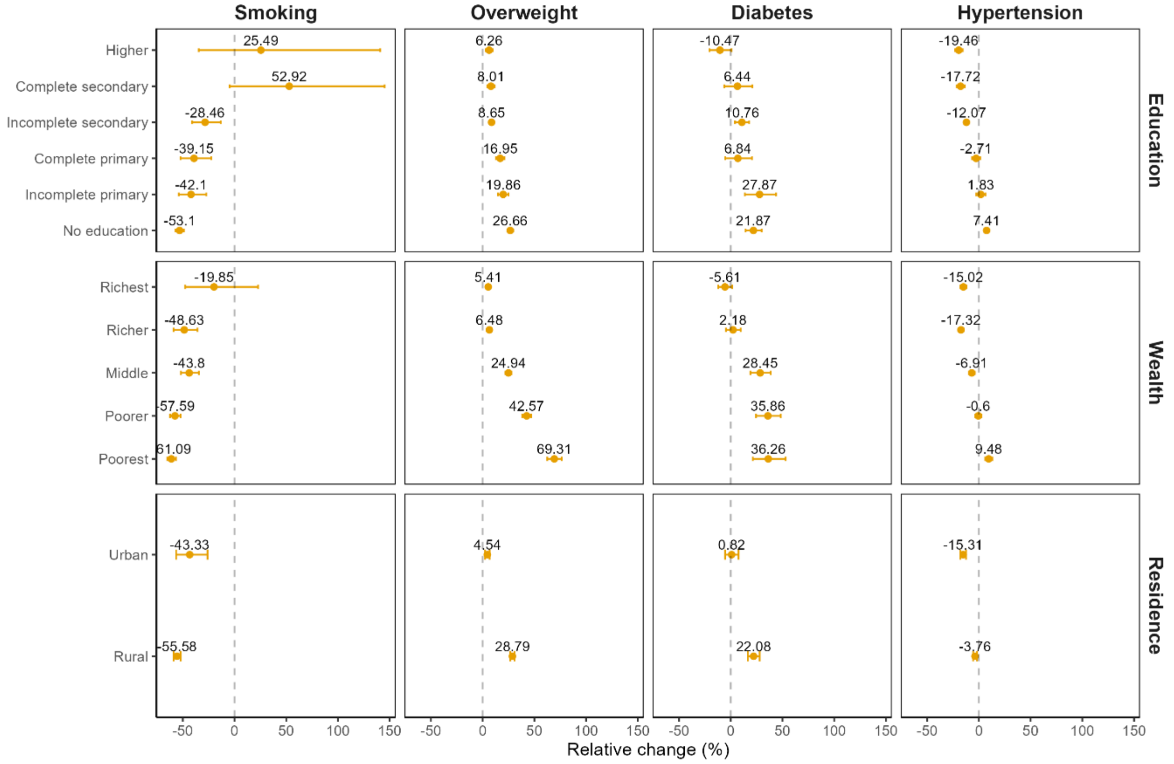

Figure S85. Age-standardized relative change in each CVD risk factor among women aged 15-49 by wealth quintile, level of education, and place of residence.

Men

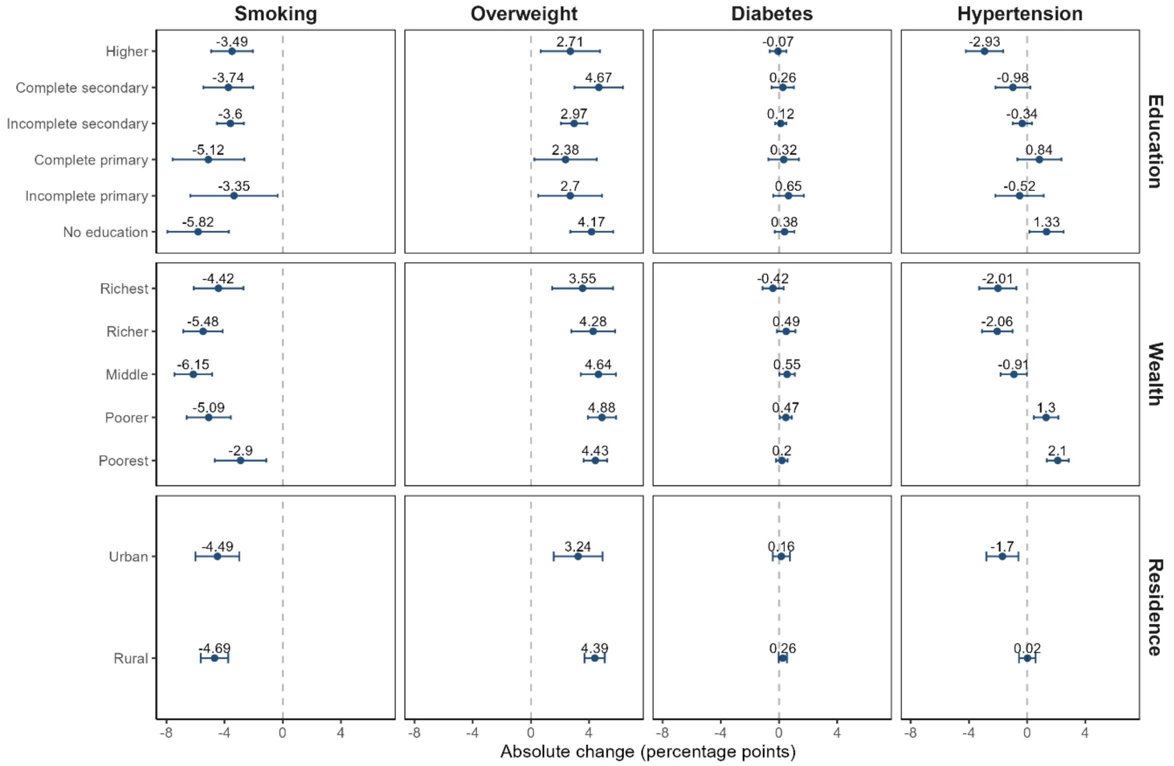

Figure S86. Age-standardized absolute change in each CVD risk factor among men aged 15-49 by wealth quintile, level of education, and place of residence.

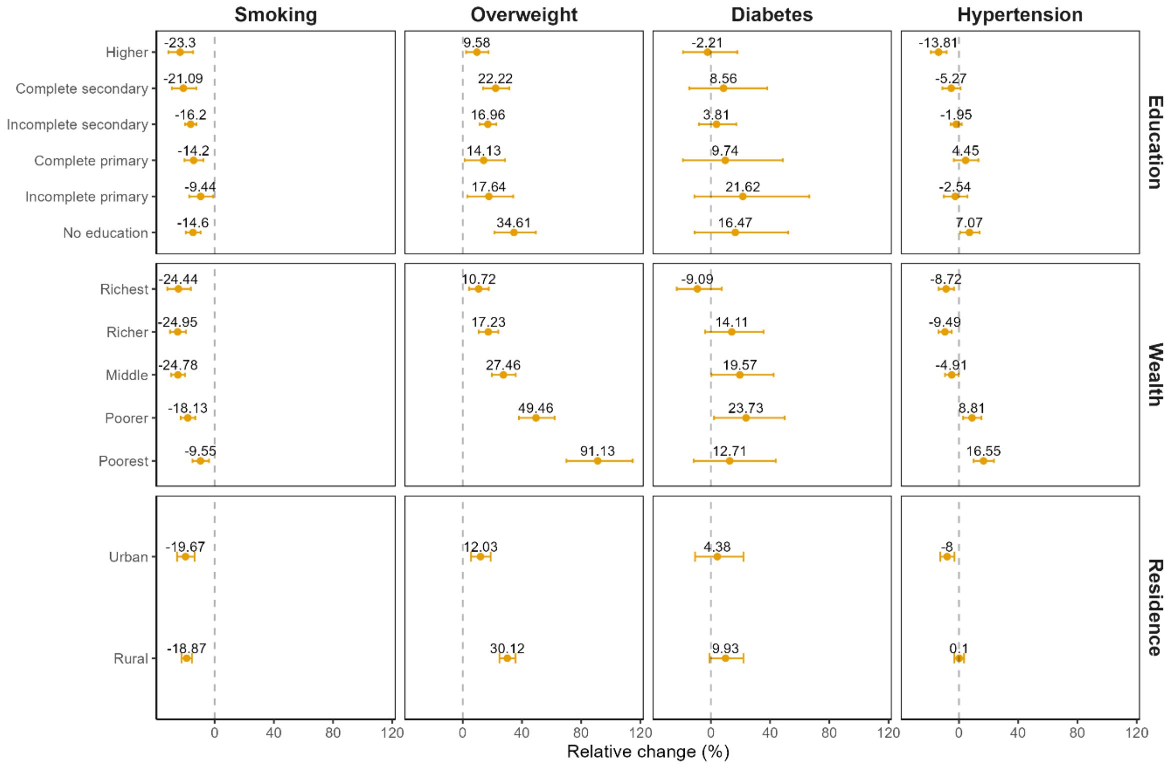

Figure S87. Age-standardized relative change in each CVD risk factor among men aged 15-49 by wealth quintile, level of education, and place of residence.

Results within the subsample of states with pre-pandemic fieldwork

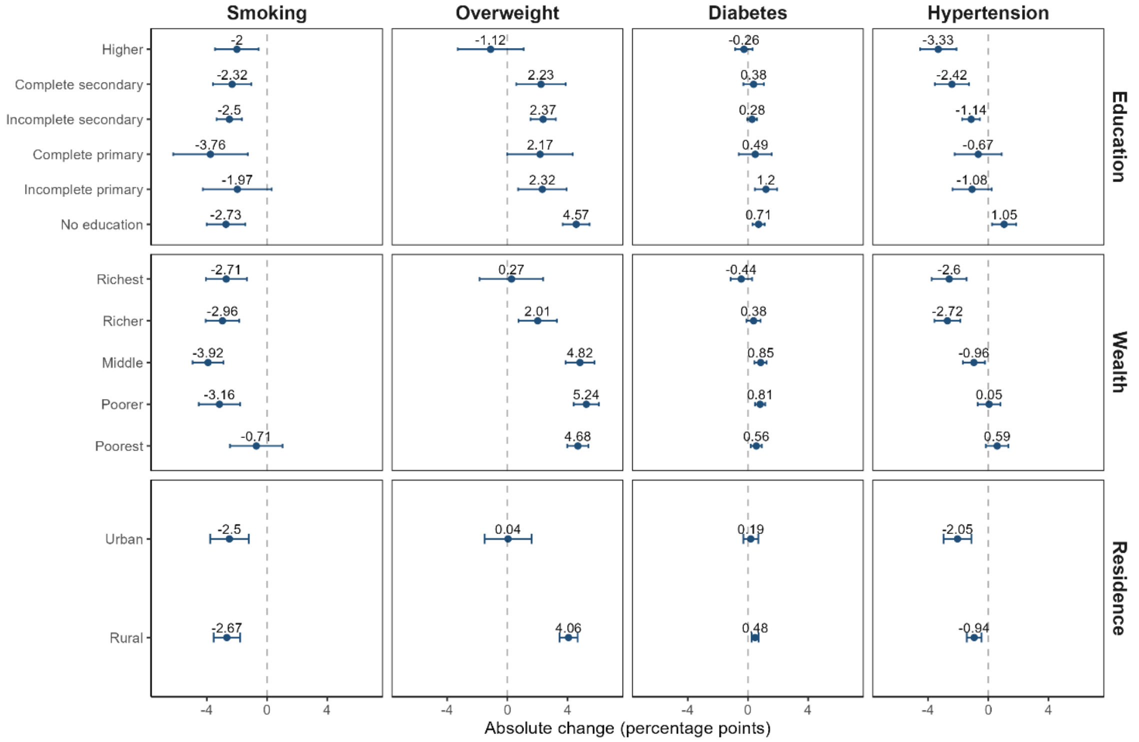

Figure S88. Age-standardized absolute change in each CVD risk factor in the subsample of states with NFHS-5 fieldwork before the beginning of the first COVID-19 wave in India by wealth quintile, level of education, and place of residence

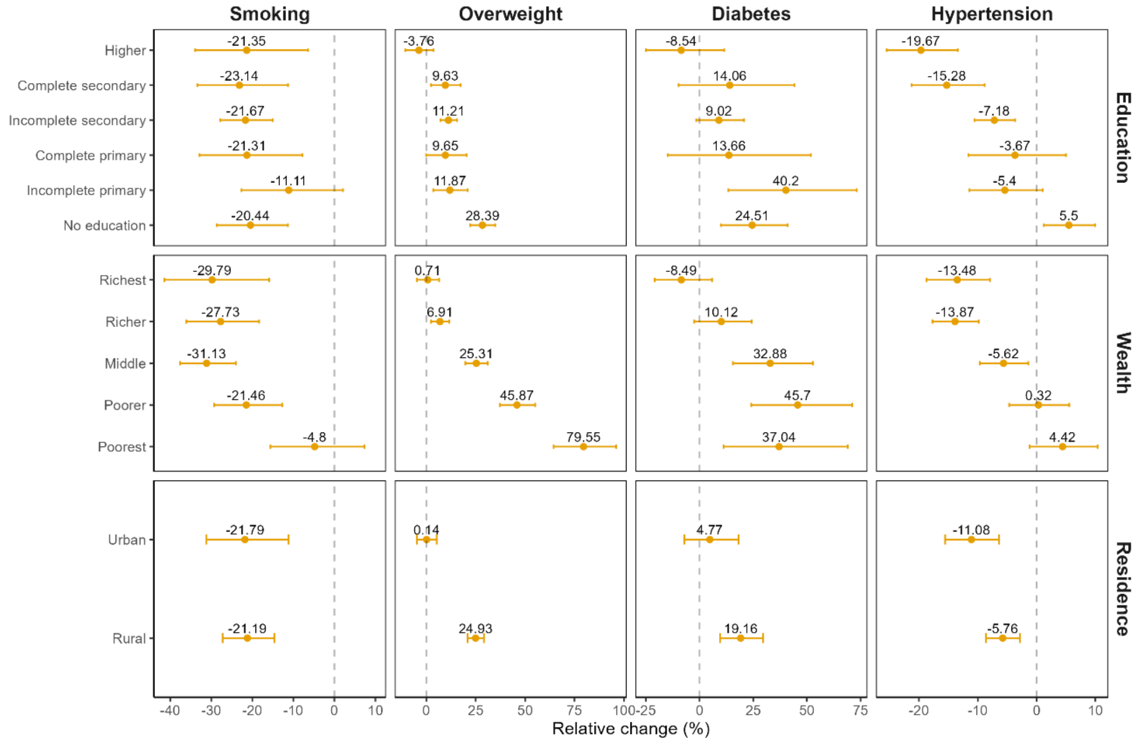

Figure S89. Age-standardized relative change in each CVD risk factor in the subsample of states with NFHS-5 fieldwork before the beginning of the first COVID-19 wave in India by wealth quintile, level of education, and place of residence

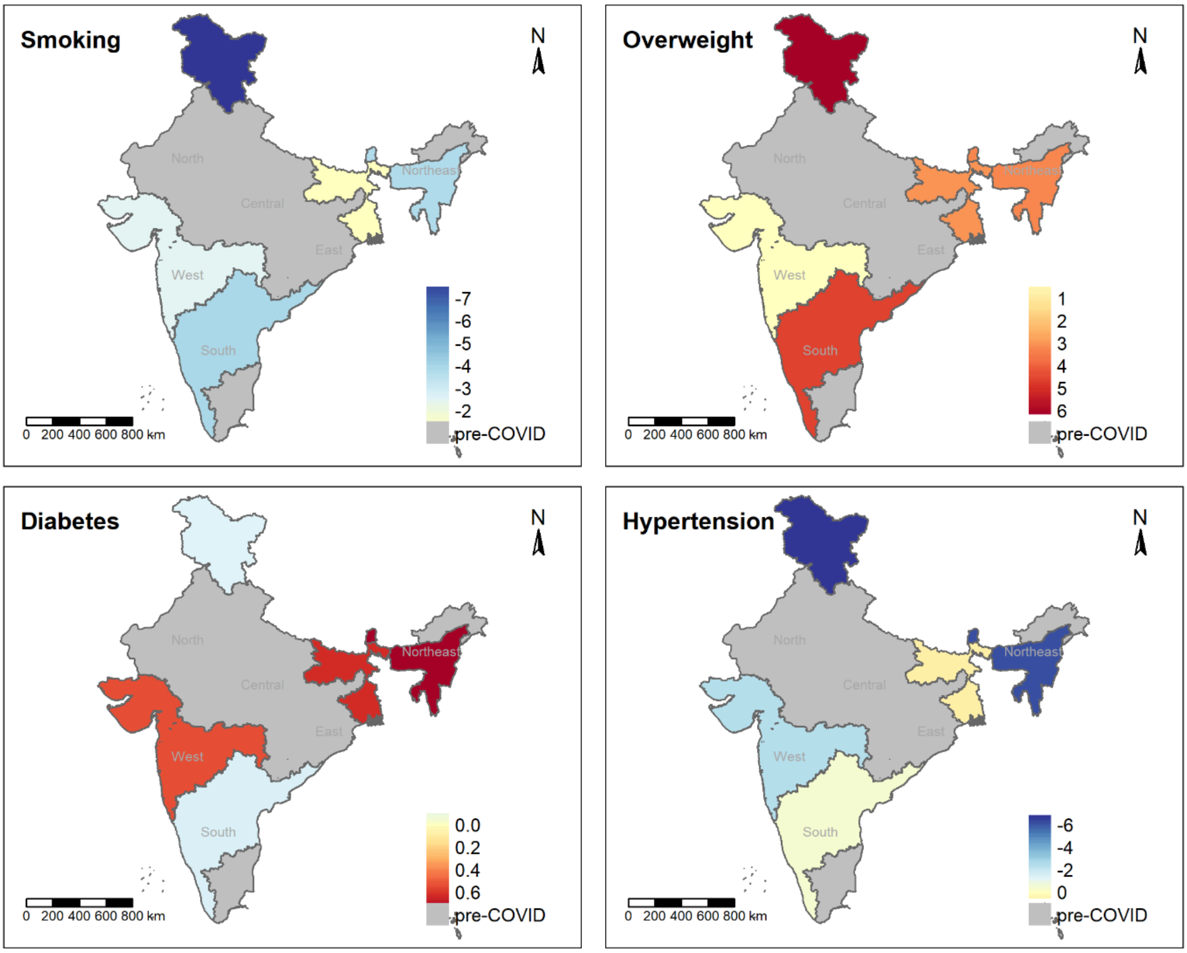

Figure S90. Age-standardized absolute changes (percentage points) in the prevalence of CVD risk factors across regions in the subsample of states with NFHS-5 fieldwork before the beginning of the first COVID-19 wave in India

Regression coefficients

Models to estimate national trends

	Smoking<br>*PR [CI]*	Overweight<br>*PR [CI]*	Diabetes<br>*PR [CI]*	Hypertension<br>*PR [CI]*
NFHS5	0.74<br>[0.71,0.77]	1.17<br>[1.15,1.19]	1.07<br>[1.02,1.13]	0.94<br>[0.92,0.95]

Table S4. Regression results for models used to assess national trends
Abbreviations: PR= prevalence ratio, CI=95% confidence interval, NFHS5=National Family Health Survey number 5. The prevalence ratios and confidence intervals were obtained by exponentiating the results from log binomial regression models with the survey year as predictor. Base level: NFHS-4. The complex survey design, including the survey weights, was taken into account. Before estimation, survey weights were adjusted to achieve age-standardization.

Models to estimate group-specific trends

Level of education

	Smoking<br>*PR [CI]*	Overweight<br>*PR [CI]*	Diabetes<br>*PR [CI]*	Hypertension<br>*PR [CI]*
NFHS5	0.74<br>[0.69,0.79]	1.30<br>[1.26,1.34]	1.21<br>[1.12,1.32]	1.07<br>[1.05,1.10]
Incomplete primary	1.31<br>[1.23,1.40]	1.18<br>[1.12,1.23]	1.22<br>[1.07,1.38]	1.03<br>[0.98,1.07]
Complete primary	1.27<br>[1.20,1.35]	1.30<br>[1.25,1.36]	1.26<br>[1.10,1.44]	0.98<br>[0.94,1.02]
Incomplete secondary	0.91<br>[0.87,0.95]	1.26<br>[1.23,1.30]	1.09<br>[1.01,1.18]	0.86<br>[0.83,0.88]
Complete secondary	0.77<br>[0.70,0.84]	1.41<br>[1.36,1.47]	1.03<br>[0.90,1.17]	0.89<br>[0.85,0.93]
Higher	0.66<br>[0.60,0.72]	1.79<br>[1.71,1.87]	1.08<br>[0.96,1.22]	0.96<br>[0.92,1.01]
Incomplete primary # NFHS5	1.12<br>[1.01,1.25]	0.93<br>[0.87,0.99]	1.04<br>[0.87,1.23]	0.93<br>[0.88,0.98]
Complete primary # NFHS5	1.07<br>[0.97,1.18]	0.91<br>[0.86,0.96]	0.89<br>[0.75,1.06]	0.93<br>[0.89,0.98]
Incomplete secondary # NFHS5	1.03<br>[0.96,1.11]	0.88<br>[0.84,0.91]	0.87<br>[0.79,0.97]	0.86<br>[0.84,0.89]
Complete secondary # NFHS5	0.96<br>[0.85,1.08]	0.89<br>[0.85,0.94]	0.87<br>[0.73,1.04]	0.82<br>[0.78,0.86]
Higher # NFHS5	0.94<br>[0.82,1.07]	0.83<br>[0.79,0.88]	0.76<br>[0.66,0.89]	0.77<br>[0.73,0.81]

Table S5. Regression results for models used to assess trends across levels of education
Abbreviations: PR= prevalence ratio, CI=95% confidence interval, NFHS5=National Family Health Survey number 5. The prevalence ratios and confidence intervals were obtained by exponentiating the results from log binomial regression models with the survey year, education, and an interaction term between education and the survey year as predictors. Base level: NFHS-4#No education. The complex survey design, including the survey weights, was taken into account. Before estimation, survey weights were adjusted to achieve age-standardization.

Wealth quintile

	Smoking *PR [CI]*	Overweight *PR [CI]*	Diabetes *PR [CI]*	Hypertension *PR [CI]*
NFHS5	0.84 [0.79,0.91]	1.79 [1.69,1.89]	1.24 [1.09,1.42]	1.13 [1.09,1.17]
Poorer	0.96 [0.91,1.01]	1.97 [1.87,2.07]	1.27 [1.11,1.45]	1.16 [1.11,1.20]
Middle	0.86 [0.82,0.91]	3.30 [3.14,3.46]	1.79 [1.57,2.04]	1.39 [1.34,1.44]
Richer	0.75 [0.71,0.80]	4.91 [4.67,5.15]	2.51 [2.21,2.84]	1.62 [1.56,1.69]
Richest	0.63 [0.57,0.69]	6.40 [6.09,6.72]	3.13 [2.77,3.54]	1.69 [1.62,1.77]
Poorer # NFHS5	0.90 [0.83,0.97]	0.82 [0.77,0.87]	1.04 [0.88,1.22]	0.92 [0.88,0.96]
Middle # NFHS5	0.81 [0.74,0.89]	0.71 [0.67,0.76]	0.98 [0.83,1.16]	0.83 [0.80,0.87]
Richer # NFHS5	0.81 [0.73,0.90]	0.63 [0.59,0.67]	0.87 [0.74,1.02]	0.77 [0.73,0.80]
Richest # NFHS5	0.81 [0.71,0.93]	0.61 [0.57,0.65]	0.74 [0.63,0.87]	0.78 [0.74,0.82]

Table S6. Regression results for models used to assess trends across wealth quintiles
Abbreviations: PR= prevalence ratio, CI=95% confidence interval, NFHS5=National Family Health Survey number 5. The prevalence ratios and confidence intervals were obtained by exponentiating the results from log binomial regression models with the survey year, wealth, and an interaction term between wealth and the survey year as predictors. Base level: NFHS-4#No education. The complex survey design, including the survey weights, was taken into account. Before estimation, survey weights were adjusted to achieve age-standardization.

Place of residence

	Smoking *PR [CI]*	Overweight *PR [CI]*	Diabetes *PR [CI]*	Hypertension *PR [CI]*
NFHS5	0.73 [0.67,0.79]	1.09 [1.05,1.12]	1.03 [0.94,1.12]	0.88 [0.85,0.91]
Rural	1.06 [1.00,1.13]	0.51 [0.50,0.53]	0.60 [0.55,0.65]	0.81 [0.78,0.84]
Rural # NFHS5	1.02 [0.93,1.12]	1.19 [1.15,1.24]	1.11 [1.00,1.24]	1.11 [1.07,1.16]

Table S7. Regression results for models used to assess stratified by place of residence
Abbreviations: PR= prevalence ratio, CI=95% confidence interval, NFHS5=National Family Health Survey number 5. The prevalence ratios and confidence intervals were obtained by exponentiating the results from log binomial regression models with the survey year, place of residence, and an interaction term between place of residence and the survey year as predictors. Base level: NFHS-4#Poor. The complex survey design, including the survey weights, was taken into account. Before estimation, survey weights were adjusted to achieve age-standardization.

Regions

	Smoking *PR [CI]*	Overweight *PR [CI]*	Diabetes *PR [CI]*	Hypertension *PR [CI]*
NFHS5	0.77 [0.72,0.82]	1.19 [1.15,1.23]	1.22 [1.10,1.35]	0.88 [0.85,0.91]
North Eastern	1.37 [1.28,1.47]	0.69 [0.66,0.73]	1.16 [1.01,1.33]	1.14 [1.09,1.20]
Central	0.93 [0.88,0.98]	0.66 [0.64,0.68]	0.94 [0.85,1.03]	0.69 [0.66,0.72]
Eastern	0.93 [0.86,1.02]	0.70 [0.67,0.73]	1.43 [1.28,1.60]	0.77 [0.73,0.80]
Western	0.49 [0.44,0.55]	1.10 [1.04,1.16]	1.18 [1.04,1.35]	0.85 [0.80,0.90]
Southern	0.89 [0.82,0.96]	1.38 [1.33,1.43]	2.64 [2.38,2.94]	1.16 [1.10,1.22]
North Eastern # NFHS5	1.05 [0.94,1.18]	1.03 [0.96,1.11]	1.12 [0.93,1.35]	0.84 [0.79,0.88]
Central # NFHS5	0.89 [0.81,0.97]	1.12 [1.07,1.17]	0.96 [0.84,1.10]	1.42 [1.35,1.49]
Eastern # NFHS5	1.19 [1.05,1.35]	1.03 [0.97,1.10]	0.97 [0.84,1.13]	1.15 [1.09,1.22]
Western # NFHS5	0.86 [0.72,1.03]	0.85 [0.79,0.91]	1.02 [0.85,1.22]	0.98 [0.91,1.04]
Southern # NFHS5	0.77 [0.69,0.86]	1.01 [0.96,1.05]	0.76 [0.66,0.87]	0.91 [0.86,0.97]

Table S8. Regression results for models used to assess trends across regions
Abbreviations: PR= prevalence ratio, CI=95% confidence interval, NFHS5=National Family Health Survey number 5. The prevalence ratios and confidence intervals were obtained by exponentiating the results from log binomial regression models with the survey year, region, and an interaction term between region and the survey year as predictors. Base level: NFHS-4#Northern. The complex survey design, including the survey weights, was taken into account. Before estimation, survey weights were adjusted to achieve age-standardization.

EAG membership

	Smoking *PR [CI]*	Overweight *PR [CI]*	Diabetes *PR [CI]*	Hypertension *PR [CI]*
NFHS5	0.76 [0.71,0.81]	1.15 [1.12,1.18]	1.09 [1.02,1.17]	0.83 [0.81,0.85]
EAG	0.93 [0.88,0.97]	0.56 [0.55,0.58]	0.56 [0.53,0.61]	0.70 [0.68,0.72]
EAG # NFHS5	0.96 [0.88,1.03]	1.11 [1.07,1.15]	1.00 [0.91,1.10]	1.37 [1.33,1.42]

Table S9. Regression results for models used to assess trends stratified by EAG status
Abbreviations: PR= prevalence ratio, CI=95% confidence interval, NFHS5=National Family Health Survey number 5, EAG=Empowered Action Group states. The prevalence ratios and confidence intervals were obtained by exponentiating the results from log binomial regression models with the survey year, EAG, and an interaction term between EAG and the survey year as predictors. Base level: NFHS-4#No EAG memberstate. The complex survey design, including the survey weights, was taken into account. Before estimation, survey weights were adjusted to achieve age-standardization.

References

1 Ahmad OB, Boschi Pinto C, Lopez A, Murray C, Lozano R, Inoue M. Age Standardization of Rates: A New WHO Standard. World Health Organization, 2001.

2 United Nations, Department of Economic and Social Affairs, Population Division. World Population Prospects 2019. Annual total population by five-year age groups. https://population.un.org/wpp/Download/Standard/Population/ (accessed Nov 15, 2021).
